# The Debiased Spatial Whittle likelihood

**DOI:** 10.1111/rssb.12539

**Published:** 2022-07-20

**Authors:** Arthur P. Guillaumin, Adam M. Sykulski, Sofia C. Olhede, Frederik J. Simons

**Affiliations:** ^1^ Queen Mary University of London London UK; ^2^ Lancaster University Lancaster UK; ^3^ École Polytechnique Fédérale de Lausanne Lausanne Switzerland; ^4^ University College London London UK; ^5^ Princeton University Princeton New Jersey USA

**Keywords:** aliasing, irregular boundaries, missing data, random fields, Whittle likelihood

## Abstract

We provide a computationally and statistically efficient method for estimating the parameters of a stochastic covariance model observed on a regular spatial grid in any number of dimensions. Our proposed method, which we call the Debiased Spatial Whittle likelihood, makes important corrections to the well‐known Whittle likelihood to account for large sources of bias caused by boundary effects and aliasing. We generalize the approach to flexibly allow for significant volumes of missing data including those with lower‐dimensional substructure, and for irregular sampling boundaries. We build a theoretical framework under relatively weak assumptions which ensures consistency and asymptotic normality in numerous practical settings including missing data and non‐Gaussian processes. We also extend our consistency results to multivariate processes. We provide detailed implementation guidelines which ensure the estimation procedure can be conducted in O(nlogn) operations, where *n* is the number of points of the encapsulating rectangular grid, thus keeping the computational scalability of Fourier and Whittle‐based methods for large data sets. We validate our procedure over a range of simulated and realworld settings, and compare with state‐of‐the‐art alternatives, demonstrating the enduring practical appeal of Fourier‐based methods, provided they are corrected by the procedures developed in this paper.

## 1 INTRODUCTION

Among the challenges of modern data analysis is making sense of large volumes of spatial and spatiotemporal data. State‐of‐the‐art parameter estimation methods are based on various likelihood approximation methods designed to combine statistical and computational efficiency. Such methods are primarily reliant on spatial/pixel models (Anitescu et al., 2017; Guinness & Fuentes, 2017; Katzfuss, 2017; Stroud et al., 2017), spectral/Fourier understanding (Guinness, 2019; Kaufman et al., 2008; Matsuda & Yajima, 2009; Shaby & Ruppert, 2012) or other methods of likelihood approximation (Banerjee et al., 2008; Lee & Mitchell, 2013; Sang & Huang, 2012; Stein et al., 2004). Fourier methods, typically based on the Whittle likelihood, are fast and scale well to massive data sets. Fourier‐based methods, on the other hand, are known to engender large sources of bias, particularly in dimensions greater than one (Dahlhaus & Künsch, 1987), in the presence of missing data, or under irregular sampling (Fuentes, 2007; Matsuda & Yajima, 2009). In this paper we propose a novel methodology that simultaneously addresses these challenges for spatial data observed on a regular grid, with potentially missing data and irregular sampling boundaries, and in any number of dimensions.

The bias which we remove is due to finite‐domain effects, the multidimensional boundary and aliasing. Much of the literature on Whittle estimation has focused on modifications to the periodogram to reduce bias, such as tapering (Dahlhaus & Künsch, 1987), edge effect estimation (Robinson & Sanz, 2006) or accounting for non‐standard sampling scenarios (Fuentes, 2007; Matsuda & Yajima, 2009; Rao, 2018). The solution we propose is simple yet effective: determine the true expectation of the periodogram under the proposed model and sampling regime, and construct a quasi‐likelihood using this quantity rather than the true spectrum—further developing and generalizing a procedure recently proposed by Sykulski et al. (2019) for one‐dimensional completely observed time series. We shall show that the Debiased Spatial Whittle likelihood almost completely removes estimation bias in spatial inference, even in the presence of significant amounts of missing data, while leaving estimation variance essentially unaffected. We also establish a convergence rate under very general sampling and model assumptions.

Debiasing Whittle estimates using the expected periodogram has been notionally investigated in various more restrictive frameworks by Fernández‐Casal and Crujeiras (2010), Simons and Olhede (2013) and Deb et al. (2017). This article, however, is the first to formalize the estimation procedure by providing theoretical guarantees that apply in any number of dimensions, allow for missing and/or non‐Gaussian data, and account for aliasing and irregular sampling boundaries. To achieve this we introduce the concept of *significant correlation contribution*, which provides weak conditions on sampling regimes that allow for asymptotically consistent parameter estimation—leveraging ideas from modulated time series proposed by Guillaumin et al. (2017). Boundary effects play a significant role as *d*, the dimensionality of the sampling domain, increases: the bias for a *d*‐dimensional cube with side *l* scales like 1/*l* while the standard deviation scales like $1/l^{d/2}$. Thus for *d* > 2 the bias is of primary significance, and it is important even for *d* = 2. This paper is also the first to provide fast O(nlogn) computational implementation, including for missing data and higher dimensions. We also prove consistency for multivariate processes which may exhibit different missingness patterns across components.

We establish the choice of notation and assumptions in Section 2. We propose our spatial quasi‐likelihood in Section 3. In Section 4 we introduce significant correlation contribution (SCC), with conditions guaranteeing consistent estimation under a wide range of sampling schemes. Section 5 develops our theoretical results which include consistency, convergence rates, and asymptotic normality of parameter estimates in a wide range of settings. Section 6 shows the improved performance on simulated data, and on actual data of Venus' topography. We conclude with discussion in Section 7.

## 2 NOTATION AND ASSUMPTIONS

Consider a finite‐variance and zero‐mean random field *X*(**s**), for $s\in R^{d}$, where *d* ≥ 1 is a positive integer. Under the assumption of homogeneity, we denote the covariance function of *X*(**s**) by $c_{X}\left(u\right)$, $u\in R^{d}$, and assume the existence of a positive piecewise continuous Riemann‐integrable spectral density function $f_{X}\left(\omega \right)$, such that $\forall u,s\in R^{d},$

(1) $$c_{X}\left(u\right)=E\left\{X(s)X(s+u)\right\}=\int_{R^{d}}f_{X}\left(\omega \right)\exp\left(i\omega \cdot u\right)d\omega ,$$

and $f_{X}(\omega )={\left(2\pi \right)}^{-d}\int_{R^{d}}c_{X}(u)\exp(-i\omega \cdot u)du$. We shall assume the spectral density belongs to a parametric family indexed by the parameter ***γ*** ∈ Θ, with $f_{X}\left(\omega \right)=f\left(\omega ;\theta \right)$, denoting the true parameter value by ***θ*** ∈ Θ. The random field *X*(**s**) is taken to be homogeneous but not necessarily isometric. We denote $n=\left(n_{1},\ldots ,n_{d}\right)\in {\left(N^{+}\right)}^{d}$, with $N^{+}$ the set of positive integers, the dimensions of an orthogonal regular and rectangular *bounding grid*, defined by

(2) $$J_{n}=\{\delta \;\circ \;{\left[x_{1},\ldots ,x_{d}\right]}^{T}:\left(x_{1},\ldots ,x_{d}\right)\in N^{d},\;0\leq x_{i}\leq n_{i}-1,i=1,\ldots ,d\},$$

and denote by $\left|n\right|=\prod_{i=1}^{d}n_{i}$ the total number of points of this grid. We denote by $X_{s},\;s\in J_{n}$ the values of the process on the grid. In Equation (2), the quantity $\delta \in {\left(R^{+}\right)}^{d}$ indicates the regular spacing along each axis, with $R^{+}$ the set of positive real numbers, and ∘ denotes the pointwise Hadamard product between two vectors. We always take $\delta ={\left[1,\ldots ,1\right]}^{T}$ for simplicity, yet without loss of generality. We write $f_{X,\delta }\left(\omega \right)$ for the spectral density of the sampled process, the *aliased* spectral density, defined by

(3) $$f_{X,\delta }\left(\omega \right)=\sum_{u\in Z^{d}}f_{X}\left(\omega +2\pi u\right),\;\omega \in R^{d},$$

which is a Fourier pair with $c_{X}\left(u\right)=\int_{T^{d}}f_{X,\delta }\left(\omega \right)\exp\left(i\omega \cdot u\right)d\omega$, $\forall u\in Z^{d}$, and T=[0,2π), with Z the set of integers.

To account for irregular domain shapes and missing data, we define a deterministic modulation value $g_{s}$ at each location of the grid $J_{n}$. If a point on the regular grid is missing then $g_{s}=0$, otherwise $g_{s}=1$. By convention, $g_{s}$ is extended to the whole set $Z^{d}$, defining $g_{s}=0$ if $s\notin J_{n}$. Using this notation, the periodogram of the observed data takes the form of

(4) $$I_{n}\left(\omega \right)=\frac{{\left(2\pi \right)}^{-d}}{\sum_{s\in J_{n}}{g_{s}}^{2}}{\left|\sum_{s\in J_{n}}g_{s}X_{s}\exp(-i\omega \cdot s)\right|}^{2},\;\;\omega \in R^{d},$$

where normalizing by $\sum_{s\in J_{n}}{g_{s}}^{2}$ rescales the periodogram for missing data, as performed by Fuentes (2007). Note that, despite this similarity, our approach is fundamentally different to that of Fuentes (2007), where this extended definition of the periodogram is used in the Whittle procedure to address missing data. While this uniform rescaling is central to the method proposed by Fuentes (2007), it is merely a convention in our case. In practice, this rescaling is not actually required in our implementation as it will be cancelled out by the rescaling in the expected periodogram, as we shall shortly see. Evaluating the periodogram on the multidimensional Fourier grid

$$\prod_{j=1}^{d}\{2\pi kn_{j}^{-1}:k=0,\ldots ,n_{j}-1\}$$

associated with the spatial grid $J_{n}$ requires O(|n|log|n|) elementary operations using the Fast Fourier Transform (FFT). If a taper is used in the spectral estimate of Equation (4), then the values of the taper are directly incorporated into $g_{s}$, such that $g_{s}$ is proportional to the taper at locations where data are observed (and still set to zero otherwise). We shall assume that $g_{s}$ takes values in the interval [0, 1] as would be the case when using the periodogram, however, this condition could be relaxed to assuming an upper bound for the absolute value.

## 3 METHODOLOGY

We shall now introduce the Debiased Spatial Whittle likelihood and an algorithm for its computation that only requires FFTs, even in the scenario of missing data and general boundaries. Thus our estimation method retains the O(|n|log|n|) computational cost of frequency‐domain approaches for regular grids.

### 3.1 Estimation procedure

Exact likelihood has optimal statistical properties in the framework of an increasing domain (Mardia & Marshall, 1984), however it is computationally inadequate for large data sets of spatial observations due to the determinant calculation and linear system that needs to be solved. A common approach is to trade off computational cost with statistical efficiency by using approximations of the likelihood function (Fuentes, 2007; Guinness & Fuentes, 2017; Varin et al., 2011). Such functions are commonly called quasi‐likelihood methods. Our proposed estimation method uses the following quasi‐likelihood, which we call the Debiased Spatial Whittle Likelihood,

(5) $$\ell \left(\gamma \right)={\left|n\right|}^{-1}\sum_{\omega \in \Omega_{n}}\left\{\log{\bar{I}}_{n}\left(\omega ;\gamma \right)+\frac{I_{n}\left(\omega \right)}{{\bar{I}}_{n}\left(\omega ;\gamma \right)}\right\}$$

where, for all ***γ*** ∈ Θ,

(6) $${\bar{I}}_{n}\left(\omega ;\gamma \right)=E_{\gamma }\left\{I_{n}\left(\omega \right)\right\},\;\forall \omega \in T^{d},$$

is the expected periodogram given the modulation values $g_{s}$, under the mean‐zero distribution of $X_{s}$ with covariance structure specified by the parameter vector ***γ***—see also Fernández‐Casal and Crujeiras (2010). In Section 4.3.4 we describe the multivariate extension to Equation (22). Note that in Equation (5) the summation is over $\Omega_{n}\subset T^{d}$. It is common to use the *natural* set of Fourier frequencies $\Omega_{n}^{\left(1\right)}\equiv \prod_{j=1}^{d}\{2k\pi n_{j}^{-1}:k=0,\ldots ,n_{j}-1\}$ for $\Omega_{n}$ in Whittle estimation, or a subset of these for semi‐parametric modelling. To ensure identifiability in degenerate sampling scenarios, when one or more of the dimensions of the domain are not growing to infinity, we shall set $\Omega_{n}$ to be the set of Fourier frequencies $\Omega_{n}^{\left(2\right)}\equiv \prod_{j=1}^{d}\{k\pi n_{j}^{-1}:k=0,\ldots ,2n_{j}-1\}$ in our theoretical developments. In practice, we shall use the natural set of Fourier frequencies $\Omega_{n}^{\left(1\right)}$ in our simulations and real‐data example, as this is computationally faster and the practical difference was found to be insignificant.

Replacing ${\bar{I}}_{n}\left(\omega ;\gamma \right)$ with $f_{X}\left(\omega ;\gamma \right)$ in Equation (5) yields the discretized form of the standard Whittle likelihood. Note, however, that unlike the spectral density $f_{X}\left(\omega \right)$, the expected periodogram ${\bar{I}}_{n}\left(\omega ;\gamma \right)$ directly accounts for the sampling, as it depends on the dimensions of the lattice **n** and on the modulation values $g_{s}$ that account for missing data. We minimize Equation (5) over Θ to obtain our estimate,

(7) $$\hat{\theta }=\arg\;\min_{\gamma \in \Theta }\left\{\ell \left(\gamma \right)\right\}.$$

By minimizing Equation (5), we find the maximum‐likelihood estimate of the data under the following parametric model,

(8) $$I_{n}\left(\omega \right)\overset{i.i.d.}{∼}\mathrm{Exp}\{{\bar{I}}_{n}^{\;‐1}\left(\omega ;\theta \right)\},\;\;\omega \in \Omega_{n},$$

where Exp(*λ*) stands for the exponential distribution with parameter *λ*. Hence the quantity given in Equation (5) can be seen as a composite likelihood (Bevilacqua & Gaetan, 2015; Varin et al., 2011). We also observe that $\nabla_{\theta }\ell \left(\theta \right)=0$ such that our method fits within the general theory of estimating equations (Heyde, 1997; Jesus & Chandler, 2017).

### 3.2 Computation of the expected periodogram

In this section we show how the expected periodogram in Equation (5) can be computed using FFTs such that our quasi‐likelihood remains an O(|n|log|n|) procedure, for any dimension *d*, and independently of the missing data patterns. Direct calculations show that the expected periodogram is the convolution of the spectral density of the process with the multi‐dimensional kernel $F_{n}\left(\omega \right)$,

$${\bar{I}}_{n}\left(\omega ;\gamma \right)=\left\{f_{X}\left(\;\cdot \;;\gamma \right)*F_{n}\left(\cdot \right)\right\}\left(\omega \right)=\int_{T^{d}}f_{X,\delta }\left(\omega -\omega^{\prime };\gamma \right)F_{n}\left(\omega^{\prime }\right)d\omega^{\prime },$$

where

(9) $$F_{n}\left(\omega \right)=\frac{{\left(2\pi \right)}^{-d}}{\sum g_{s}^{2}}{\left|\sum_{s\in J_{n}}g_{s}\exp\left(i\omega \cdot s\right)\right|}^{2},\;\omega \in R^{d}.$$

When $g_{s}=1,\forall s\in J_{n}$, $F_{n}\left(\omega \right)$ is simply the multi‐dimensional rectangular Féjer kernel, that is, a separable product of one‐dimensional Féjer kernels. For this reason we call $F_{n}\left(\omega \right)$ a *modified* Féjer kernel. We now provide two lemmata stating that the expected periodogram can be computed via FFTs for any value of the modulation $g_{s}$ on the grid $J_{n}$.

Lemma 1 (Expected periodogram as a Fourier series) *The expected periodogram can be written as the following Fourier series,*

(10) $${\bar{I}}_{n}\left(\omega ;\gamma \right)={\left(2\pi \right)}^{-d}\sum_{u\in Z^{d}}{\bar{c}}_{n}\left(u;\gamma \right)\exp\left(-i\omega \cdot u\right),\;\forall \omega \in T^{d},\forall \gamma \in \Theta ,$$

*where* ${\bar{c}}_{n}\left(u;\gamma \right)$ *is defined by*

(11) $${\bar{c}}_{n}\left(u;\gamma \right)=c_{g,n}\left(u\right)c_{X}\left(u;\gamma \right),\;u\in Z^{d},\;\mathrm{with}$$

(12) $$c_{g,n}\left(u\right)=\frac{\sum_{s\in J_{n}}g_{s}g_{s+u}}{\sum_{s\in J_{n}}g_{s}^{2}},\;u\in Z^{d}.$$

Direct calculation upon taking the expectation of the periodogram as defined in Equation (4).

Note that, having set $g_{s}$ to take value zero outside of the sampling domain, we can rewrite Equation (12) as

(13) $$c_{g,n}\left(u\right)=\frac{\sum_{s\in Z^{d}}g_{s}g_{s+u}}{\sum_{s\in Z^{d}}g_{s}^{2}},\;u\in Z^{d}.$$

In practice we can evaluate the expected periodogram at the set of Fourier frequencies through a multidimensional FFT, as detailed in the following lemma.

Lemma 2 (Computation of the expected periodogram via FFT) *The expected periodogram can be expressed as*

(14) $${\bar{I}}_{n}\left(\omega ;\gamma \right)={\left(2\pi \right)}^{-d}\sum_{u_{1}=0}^{n_{1}-1}\ldots \sum_{u_{d}=0}^{n_{d}-1}{\tilde{c}}_{n}\left(u\right)\exp\left(-i\omega_{k}\cdot u\right),\;\forall \omega \in \Omega_{n}^{\left(1\right)},$$

*where*

(15) $${\tilde{c}}_{n}\left(u\right)=\sum_{q}{\bar{c}}_{n}\left(u-q\;\circ \;n;\gamma \right),\;u\in Z^{d},$$

*and where the sum over* **q** *ranges over all vectors of size d with elements in the set {0, 1} (hence,* $2^{d}$ *of them), and where ∘ denotes the Hadamard product. Thus the expected periodogram can be computed via FFT. Note that* ${\tilde{c}}_{n}$ *is a periodized version of* ${\bar{c}}_{n}$ *as* ${\tilde{c}}_{n}\left(u-q\;\circ \;n\right)={\tilde{c}}_{n}\left(u\right).$

Please see the Supplementary Material.

As an example, in dimension *d* = 2, *q* takes values in $\left\{{\left[0 0\right]}^{T},{\left[1 0\right]}^{T},{\left[0 1\right]}^{T},{\left[1 1\right]}^{T}\right\}$, and Equation (14) therefore takes the form

$$\begin{array}{cc} {\bar{I}}_{n}\left(\omega ;\gamma \right) & \;={\left(2\pi \right)}^{-d}\sum_{u_{1}=0}^{n_{1}-1}\sum_{u_{2}=0}^{n_{2}-1}\left\{{\bar{c}}_{n}\left(u_{1},u_{2};\gamma \right)+{\bar{c}}_{n}\left(u_{1}-n_{1},u_{2}-n_{2};\gamma \right)\right.\\ & \;+\left.{\bar{c}}_{n}\left(u_{1},u_{2}-n_{2};\gamma \right)+{\bar{c}}_{n}\left(u_{1}-n_{1},u_{2};\gamma \right)\right\}\exp(-i\omega_{k}\cdot u). \end{array}$$

We remind the reader that $g_{s}$ is defined to be zero outside $J_{n}$. Hence, in the case of no tapering, $c_{g,n}\left(u\right)$ in Equation (12) is the ratio of the number of pairs of observations *separated* by the vector **u** over the total number of *observed* points of the rectangular grid $J_{n}$. In the special case of complete observations on the rectangular grid, Equation (12) simplifies to

(16) $$c_{g,n}\left(u\right)=\left\{\begin{array}{cc} {\left|n\right|}^{‐1}\prod_{i=1}^{d}\left(n_{i}‐\left|u_{i}\right|\right)=\prod_{i=1}^{d}\left(1‐\frac{\left|u_{i}\right|}{n_{i}}\right) & \text{if}\;|u_{i}|\leq n_{i}‐1,i=1,\ldots ,d,\\ 0 & \text{otherwise}. \end{array}\right.$$

which is a multidimensional form of the triangle kernel found in Percival and Walden (1993, p. 198) for the expected periodogram of regularly sampled time series. In the general case, $c_{g,n}\left(u\right)$ is precomputed for all relevant values of **u** via an FFT independently of the parameter value ***γ***, such that our method can be applied to scenarios of missing data without loss of computational efficiency. Similarly, we can combine our debiasing procedure with tapering by using a tapered spectral estimate for $I_{n}\left(\omega \right)$ in Equation (5) with adjusted values for $g_{s}$ (as discussed at the end of Section 2). The expected periodogram, ${\bar{I}}_{n}\left(\omega ;\gamma \right)$, is then computed on $\Omega_{n}$ by using these values of $g_{s}$ in the formulation of $c_{g,n}\left(u\right)$ in Equation (12). Combining debiasing and tapering therefore remains an O(|n|log|n|) procedure. The procedure of Equation (14) automatically incorporates sampling effects (geometry of the observation region, missing observations), aliasing and boundary effects in one O(|n|log|n|) operation. Note that merely calculating the aliased spectral density, and using this in the Whittle likelihood, requires knowledge of the full decay of the spectrum, and deciding on how many aliased terms to include; a procedure that in general requires non‐automatic intervention and is not guaranteed to be O(|n|log|n|).

## 4 PROPERTIES OF SAMPLING PATTERNS

To account for missing observations on the rectangular grid $J_{n}$, we replace missing values with zeros via the modulation function $g_{s}$. Depending on $g_{s}$ this may result in losing identifiability of the parameter vector from the second‐order moment quantities available from the data. More generally, we wish to understand how the sampling pattern affects the consistency of our estimation procedure. To this end, we define the notion of SCC for spatial random fields, which determines whether the sampling pattern samples enough *spatial lags* where information about the model lies. This generalizes ideas from modulated time series (Guillaumin et al., 2017). Following three simple lemmata on some properties of $c_{g,n}\left(u\right)$, we shall provide the formal definition of SCC, and follow with some general cases and an example with an isometric model family to provide more intuition and demonstrate the generality of our framework.

### 4.1 Basic properties of $c_{g,n}\left(u\right)$ and $F_{n}\left(\omega \right)$

We state three basic properties of the introduced quantity $c_{g,n}\left(u\right)$, in order to provide more intuition, but also for further use in this paper.

Lemma 3 *We have*

(17) $$0\leq c_{g,n}\left(u\right)\leq 1,\;\forall u\in Z.$$

The left side of the inequality is obvious as, by assumption, $g_{s}\geq 0$. The right side is obtained by direct application of the Cauchy–Schwarz inequality.□

Lemma 4 (Finite support) *The spatial kernel* $c_{g,n}\left(u\right)$ *vanishes for* $u\in Z^{d}$ *if for any j* = 1, …, *d*, $|u_{j}|\geq n_{j}$.

This is immediate from the definition.

Lemma 5 (Fourier pair) *The kernel* $F_{n}\left(\omega \right)$, $\omega \in T^{d}$, *defined in Equation* (9), *and* $c_{g,n}\left(u\right)$, $u\in Z^{d}$, *defined in Equation* (12), *form a Fourier pair*.

This is a direct application of the convolution theorem, having noted that $c_{g,n}\left(u\right)$ is a discrete convolution.□

### 4.2 Definitions

Our concept of SCC is defined in asymptotic terms, since we shall make use of this to establish consistency of our estimator. More specifically, we consider a sequence of grids, indexed by k∈N, which goes to infinity, rather than a single grid.

Definition 1 (Significant correlation contribution (SCC)) A sequence of observed grids ${\left(J_{n_{k}},g_{k}\right)}_{k\in N}$ leads to SCC for the model family {*f*_*X*_(·; ***γ***): ***γ*** ∈ Θ} if it satisfies both

(18) $$\left\{\begin{array}{c} \sum_{u\in Z^{d}}c_{g,n_{k}}\left(u\right)c_{X}^{2}\left(u\right)\;\underset{k\to \infty }{=}\;o\left(\sum g_{s}^{2}\right),\\ {\underline{\lim}}_{k\to \infty }S_{k}\left(\theta_{1},\theta_{2}\right)>0,\;\forall \theta_{1}\neq \theta_{2}\in \Theta , \end{array}\right.$$

where ${\underline{\lim}}_{k\to \infty }$ denotes the limit inferior and where we have defined, for all $\theta_{1},\theta_{2}\in \Theta$,

(19) $$S_{k}\left(\theta_{1},\theta_{2}\right)\equiv \sum_{u\in Z^{d}}c_{g,n_{k}}{\left(u\right)}^{2}{\left\{c_{X}\left(u;\theta_{1}\right)‐c_{X}\left(u;\theta_{2}\right)\right\}}^{2}.$$

The rationale for this definition of $S_{k}\left(\theta_{1},\theta_{2}\right)$ is that

$$S_{k}\left(\theta_{1},\theta_{2}\right)={\left(2\pi \right)}^{-d}\int_{T^{d}}{\left\{{\bar{I}}_{n_{k}}\left(\omega ;\theta_{1}\right)-{\bar{I}}_{n_{k}}\left(\omega ;\theta_{2}\right)\right\}}^{2}d\omega ,$$

due to Equation (10) and Parseval's identity for Fourier series. We remind the reader that the sums in Equations (18) and (19) are *de facto* finite for a given **n**, due to the definition of $c_{g,n}\left(u\right)$, which for fixed *n* has finite support according to Lemma 4. We observe that the above definition depends on both the sequence of grids, from $c_{g,n_{k}}\left(u\right)$, and on the model family, from $c_{X}(u;\gamma )$. In the rest of this paper we shall say that a sequence of grids leads to SCC, if the model family that this applies to is obvious from the context. In addition we define the notion of highly significant correlation contribution (HSCC), which will allow us to establish a convergence rate.

Definition 2 (Highly Significant Correlation Contribution) A sequence of observed grids ${\left(J_{n_{k}},g_{k}\right)}_{k\in N}$ leads to HSCC for the model family {*f*_*X*_(·; ***γ***): ***γ*** ∈ Θ}

a. if it leads to SCC,
b. if the covariance function is differentiable with respect to the parameter vector, and in particular, the quantity $\min_{v\in R^{p},\left\|v\right\|=1}\sum_{u\in Z^{d}}c_{g,n_{k}}^{2}\left(u\right){\left(\sum_{j=1}^{p}v_{j}\left\{\frac{\partial c_{X}}{\partial \theta_{j}}\left(u;\theta \right)\right\}\right)}^{2}$ is asymptotically lower bounded by a non‐zero value, denoted *S*(***θ***),
c. if the expected periodogram is twice differentiable with respect to the parameter vector, and such that its first and second derivatives are both upper bounded in norm by a constant denoted $M_{\partial \theta^{2}}>0$.

Note that a necessary and more intuitive condition for the second item of the above definition is that for all *j* = 1, …, *d*, $\sum_{u\in Z^{d}}c_{g,n_{k}}^{2}\left(u\right){\left[\frac{\partial c_{X}}{\partial \theta_{j}}\left(u;\gamma \right)\right]}^{2}$ be lower bounded by a positive value. Broadly speaking, the first part of Equation (18) is required so that information grows fast enough. It can be compared to necessary conditions of decaying covariances in laws of large numbers, with the additional requirement of accounting for sampling when considering spatial data. Note that the first part of Equation (18) is obviously satisfied if the sample covariance sequence is assumed square summable and the number of observations grows infinite.

The second part of Equation (18) ensures that the expected periodograms for any two parameter vectors of the parameter set remain *asymptotically distant* in terms of $L_{2}$ norm. In Lemma 11 in Section 5, we show how this transfers to the expectation of the likelihood function, ensuring that it attains its minimum at the true parameter vector uniquely. Then in Lemma 15 we show that the likelihood function converges uniformly in probability to its expectation over the parameter set, as long as the first part of Equation (18) is satisfied. This all together will eventually lead to the consistency of our inference procedure, which is the result of Theorem 1. Hence the second part of Equation (18) is required to ensure that the sampling allows to distinguish parameter vectors based on the expectation of our approximate likelihood function. To provide further understanding, we shall now consider some general cases and specific examples with respect to this definition.

### 4.3 General sampling cases and sampling example

Definition 1 extends the definition of SCC provided by Guillaumin et al. (2017) for time series in two ways. First, it provides a generalization for spatial data with the notable difference that spatial *sampling* is more complex than sampling in time. Indeed, one needs to not only account for the frequency of the sampling but also for the spatial sampling direction. Second, even in dimension one, the version provided by Guillaumin et al. (2017) implies the version provided here, while the reverse is not always true—thus relaxing the assumptions required for consistency. Specifically, in the second part of Equation (18), we do not require observing a specific finite set of lags that will allow identification of the parameters, unlike Guillaumin et al. (2017). We now provide more intuition about SCC through general cases, and a specific example.

#### 4.3.1 General sampling cases

Under standard sampling conditions, SCC takes a simpler form, as we show through the two following lemmata.

Lemma 6 (SCC for full grids) *If we observe a sequence of full rectangular grids that grow unbounded in all directions (i.e*. $n_{j}\to \infty ,j=1,\;\ldots ,\;d$), *then SCC is equivalent to the standard assumption that for any two distinct parameter vectors* $\theta_{1},\theta_{2}\in \Theta$, *the measure of the set* $\left\{\omega \in T^{d}:f_{X,\delta }\left(\omega ;\theta_{1}\right)\neq f_{X,\delta }\left(\omega ;\theta_{2}\right)\right\}$ *is positive*.

Please see the Supplementary Material.

Importantly, we do not require the growth to happen at the same rate in all directions. We do require that grids grow unbounded in all directions to obtain this equivalence when we have no further knowledge on the functional form of the spectral densities. However, in many practical cases, such as that of an isometric exponential covariance function, our results still hold if the grid grows unbounded in one direction rather than all. Another important case for practical applications is that of a fixed shape of observations that grows unbounded, which is the subject of the following lemma.

Lemma 7 (Fixed shape of observations) *Consider a fixed shape defined by a function* $\Xi :{\left[0,1\right]}^{d}\mapsto \left\{0,1\right\}$, *and let* $g_{k,s}=\Xi \left(s\circ n_{k}^{-1}\right),\forall s\in J_{n_{k}},\forall k\in N$. *If the grids grow unbounded in all directions, and if the interior of the support of* Ξ *is not empty, then SCC is again equivalent to the condition stated in Lemma* 6 *on the parametric family of spectral densities*.

Please see the Supplementary Material.

In Section 6.2 we provide a simulation study for the particular case of a circular shape of observations, which satisfies this lemma.

Finally, from a frequency‐domain point of view, the second part of SCC can be understood according to the following lemma.

Lemma 8 *The second part of SCC is equivalent to*

$$S_{k}\left(\theta_{1},\theta_{2}\right)=\int_{T^{d}}{\left|\int_{T^{d}}F_{n_{k}}\left(\omega^{\prime }\right)\left\{f_{X}\left(\omega^{\prime }-\omega ;\theta_{1}\right)-f_{X}\left(\omega^{\prime }-\omega ;\theta_{2}\right)\right\}d\omega^{\prime }\right|}^{2}d\omega >0.$$

This comes as a consequence of Lemma 5 and standard Fourier theory.

Most importantly, note that in general SCC requires more than the necessary requirement that for two distinct parameters, the expected periodograms for the sequence of grids should be non‐equal, and this is to correctly account for missing data mechanisms and their impact on consistency. To obtain SCC (c.f. [19]) this means we require that information about $\theta_{1}$ relative to $\theta_{2}$ grows as we observe ever larger patches of data. Our vulnerability to adversarial sampling will depend on the structure of the covariance pattern under study; for example if we only sample along a boundary then between points on the boundary we get information about very short scales, or between parts of the boundary only very long scales. We will now provide further intuition about SCC through a specific example.

#### 4.3.2 Examples

We consider a separable exponential covariance function (*d* = 2 here) with parameters $\rho_{1}>0$ and $\rho_{2}>0$ defined by

(20) $$c_{X}\left(u\right)=\sigma^{2}\exp\left(-\rho_{1}^{-1}\left|u_{1}\right|\right)\exp\left(-\rho_{2}^{-1}\left|u_{2}\right|\right),\;u\in R^{2}.$$

If we sample along one axis only, it is clear that the second part of SCC fails as the range parameter along the other axis cannot be identified from the data. In contrast, the second part of SCC will be satisfied for this particular model and for a full rectangular grid as long as $n_{1}\geq 2$ and $n_{2}\geq 2$. The first part of SCC is valid as long as the sample size grows to infinity, since the sample covariance function is square summable. For this model class, SCC is therefore satisfied if and only if $n_{1}\geq 2$ and $n_{2}\geq 2$ and $n_{1}n_{2}$ goes to infinity. It is also worth observing that under those conditions, the convergence rate of our estimator will be $O\left({\left(n_{1}n_{2}\right)}^{-1/2}\right)$ (see Theorem 2), irrespective of the ratio $n_{1}/n_{2}$, which, in particular, is allowed to converge to zero or infinity. The Supplementary Material provides an example where SCC fails.

These two examples show the flexibility of SCC compared to standard assumptions. They show that the two parts of SCC are complimentary and help understand their role in establishing consistency. The second part is required to ensure identifiability of the parameter vector from the expected periodogram. The first part of SCC is required to ensure that some form of a law of large numbers holds for linear combinations of the periodogram.

#### 4.3.3 Application to randomly missing data

Our extended definition of SCC can be applied to the scenario where data are missing at random, on the condition that the randomness scheme for the missing data is independent from that of the observed process. For such applications we shall say that a sequence of grids leads to SCC almost surely if (18) is satisfied almost surely under the probability that defines the missingness scheme. If a sequence of grids leads to SCC almost surely, it is easy to verify that all our consistency results derived in Section 5 still hold. Yet again for consistency we need our information about $\theta_{1}$ relative to $\theta_{2}$ to grow as we observe ever larger patches of data with randomly missing observations. This need not correspond to a linear relationship between the observed number of samples and the nominal number of samples in the observational domain, but instead depends on the true covariance of the random field under study.

A simple application of these considerations is one where each point of a rectangular grid is observed or missed according to a Bernoulli random variable (with a positive probability of being observed), independently of other points of the grid, and independently of the observed process.

#### 4.3.4 Extension to multivariate random fields

In this section we define the notation necessary for multivariate random fields. Assume we observe *p* ≥ 1 random fields jointly,

(21) $$Y_{s}^{\left(q\right)}=g_{s}^{\left(q\right)}X_{s}^{\left(q\right)},\;s\in R^{d},\;q\in \left\{1,\ldots ,p\right\},$$

and allow the observation pattern defined by the modulations $g_{s}^{\left(q\right)}$ to differ across the *p* random fields. This is a realistic observation scheme in many real‐world settings, for example, for multi‐spectral and repeated remote‐sensing observations, where cloud cover will contribute to varying degrees of censoring, yet with the underlying grids essentially unchanged (e.g. Song et al., 2018).

Just like Rao (1967) we compute the cross‐periodogram of pairs of processes. Assume we observe the *p*‐variate process $X_{s}$ and that for each process sampled at the same grid we have a masking function $g_{s}^{\left(q\right)}$ for 1 ≤ *q* ≤ *p*, so that we can incorporate some variation in sampling frequency, see, for example, Gotway and Young (2002). We calculate the DFT to be

$$J^{\left(q\right)}\left(\omega \right)=\frac{{\left(2\pi \right)}^{‐d/2}}{\sqrt{\sum_{s\in I_{n}}g_{s}^{{\left(q\right)}^{2}}}}\sum_{s}g_{s}^{\left(q\right)}X_{s}^{\left(q\right)}\exp\{-is\cdot \omega \},$$

and we collect the DFTs in the vector $J{\left(\omega \right)}^{T}=\left(J^{\left(1\right)}\left(\omega \right)\;\ldots \;J^{\left(p\right)}\left(\omega \right)\right).$ We can define the cross‐periodogram from this quantity:

$$I_{n}^{\left(qr\right)}\left(\omega \right)=J^{\left(q\right)}\left(\omega \right)J^{(r)*}\left(\omega \right).$$

We can define the expected periodogram at a given frequency ***ω*** by the *p* × *p* matrix,

$${\bar{I}}_{n}\left(\omega \right)=E\left\{J\left(\omega \right)J^{H}\left(\omega \right)\right\},$$

and this is in turn requiring us to define notation for the cross‐covariance function:

$$c_{X}^{\left(qr\right)}\left(u\right)=\text{cov}\left\{X_{s}^{\left(q\right)},X_{s+u}^{\left(r\right)}\right\}.$$

The expected periodogram matrix therefore has the elements

$$\begin{array}{cc} {\bar{I}}_{n}^{\left(qr\right)}\left(\omega \right) & =\frac{{\left(2\pi \right)}^{‐d}}{\sqrt{\sum_{s_{1}\in I_{n}}g_{s_{1}}^{{\left(q\right)}^{2}}\sum_{s_{2}\in I_{n}}g_{s_{2}}^{{\left(r\right)}^{2}}}}\sum_{s}\sum_{u}g_{s}^{\left(q\right)}g_{s+u}^{\left(r\right)}c_{X}^{\left(qr\right)}\left(u\right)\exp\left\{‐iu\cdot \omega \right\}. \end{array}$$

Then with the definition

$$c_{g,n}^{\left(qr\right)}(u)=\frac{\sum_{s\in J_{n}}g_{s}^{\left(q\right)}g_{s+u}^{\left(r\right)}}{\sqrt{\sum_{s\in J_{n}}g_{s}^{{\left(q\right)}^{2}}\sum_{s\in J_{n}}g_{s}^{{\left(r\right)}^{2}}}},$$

the expected periodogram takes the form of

$${\bar{I}}_{n}^{\left(qr\right)}\left(\omega \right)={\left(2\pi \right)}^{‐d}\sum_{u}c_{g,n}^{\left(qr\right)}\left(u\right)c_{X}^{\left(qr\right)}\left(u\right)\exp\left\{‐iu\cdot \omega \right\}.$$

The computation of the above quantity can be carried out by applying Lemma 2 for each $\left(q,r\right)\in {\left\{1,\ldots ,p\right\}}^{2}$. The Whittle likelihood is then trivially extended to this setting as was already remarked upon by Whittle (1953) and Shea (1987). The Whittle likelihood in the multivariate setting can be re‐written as (e.g. Hosoya & Taniguchi, 1982, 1993; Kakizawa, 1997):

(22) $$\ell_{n}\left(\theta \right)={\left|n\right|}^{-1}\sum_{\omega }\left\{\log\;\det\left\{\bar{I}\left(\omega ;\theta \right)\right\}+J^{H}\left(\omega \right){\bar{I}}^{-1}\left(\omega ;\theta \right)J\left(\omega \right)\right\}.$$

We can still use this for estimation, only requiring that the eigenvalues of $\bar{I}$(***ω***) are positive in the neighbourhood of θ. We extend the definition of SCC to the multivariate SCC (m‐SCC) as follows:

Definition 3 (Multivariate SCC) A sequence of observed grids ${\left(J_{n_{k}},g_{k}\right)}_{k\in N}$ leads to SCC for the multivariate model family {*f*(·; ***γ***): ***γ*** ∈ Θ} if it satisfies

(23) $$\left\{\begin{array}{c} \sum_{q,r=1}^{p}\frac{\sum_{u}c_{g}^{\left(qr\right)}\left(u\right){c_{X}^{\left(qr\right)}\left(u\right)}^{2}}{\sqrt{\sum {g_{s}^{\left(q\right)}}^{2}\sum {g_{s}^{\left(r\right)}}^{2}}}=o\left(1\right),\\ {\underline{\lim}}_{k\to \infty }S_{k}\left(\theta_{1},\theta_{2}\right)>0,\;\forall \theta_{1}\neq \theta_{2}\in \Theta , \end{array}\right.$$

where $S_{k}\left(\theta_{1},\theta_{2}\right)$ has been changed to accommodate for the multivariate scenario,

(24) $$S_{k}\left(\theta_{1},\theta_{2}\right)\equiv \sum_{q,r=1}^{p}\sum_{u\in Z^{d}}{c_{g,n_{k}}^{\left(qr\right)}\left(u\right)}^{2}{\left\{c_{X}^{\left(qr\right)}\left(u;\theta_{1}\right)-c_{X}^{\left(qr\right)}\left(u;\theta_{2}\right)\right\}}^{2},\;\forall \theta_{1},\theta_{2}\in \Theta^{2}.$$

## 5 THEORY

In this section we first provide the proof of our estimator's consistency in the general setting that encompasses both non‐Gaussian and multivariate random fields. We then also derive its rate of convergence and the asymptotic distribution in univariate Gaussian and non‐Gaussian settings. We assume the following set of assumptions holds in order to establish consistency.

Assumption 1 (Consistency assumptions)

a. The parameter set Θ is compact.
b. The aliased spectral density $f_{X,\delta }\left(\omega ;\gamma \right),\omega \in T^{d},\gamma \in \Theta$ is bounded above by $f_{\delta ,\max}<\infty$ and below by $f_{\delta ,\min}>0$. Additionally, $f_{X,\delta }\left(\omega ;\gamma \right)$ admits a derivative with respect to the parameter vector ***γ***, which is upper bounded in norm by $M_{\partial \theta }$. For a multivariate random field, we similarly require that the eigenvalues of the matrix spectral density *f*(***ω***; ***γ***) are lower and upper bounded by positive analogous constants $f_{\delta ,\min}$ and $f_{\delta ,\max}$ respectively.
c. The sequence of observation grids leads to SCC for the considered model family.
d. The modulation $g_{s}$, $s\in Z^{d}$, takes its values in the interval [0, 1].
e. The random field *X*(**s**) has finite and absolutely summable fourth‐order cumulants.

Two main asymptotic frameworks coexist in spatial data analysis, namely infill asymptotics and growing‐domain asymptotics (Zhang & Zimmerman, 2005). We study our estimator within the latter framework, which we consider most plausible for finite‐resolution remote‐sensing observations, imposing that the sample size goes to infinity (through our SCC assumption) while having fixed ***δ***. Our set of assumptions is standard, except for SCC, which generalizes the standard assumption of a fully observed rectangular grid associated with the requirement that two distinct parameter vectors map to two spectral densities that are distinct on a Lebesgue set of non‐zero measure.

Theorem 1 (Consistency) *Under Assumption* 1, *the sequence of estimates* ${\hat{\theta }}_{k}$ *defined by* Equation (7) *converges in probability to the true parameter vector* ***θ*** *as the observational domain diverges*.

This result holds for a wide class of practical applications, as

- we do not require the rectangular grid to be fully observed. We allow for a wide class of observational domains, as long as SCC is satisfied;
- we do not require the grid to grow at the same rate along all dimensions. Classical frequency‐domain results make use of the fact that the multilevel Block Toeplitz with Toeplitz Blocks covariance matrix has its eigenvalues distributed as the spectral density. However, this result only holds under the assumption that the sampling grid grows at the same rate along all dimensions.

Theorem 1 holds for Gaussian, non‐Gaussian and multivariate Gaussian random fields that satisfy the required conditions. The proof of Theorem 1 is the same for all three cases, but some lemmata and propositions on which Theorem 1 relies will require additional detail for each case. We shall prove Theorem 1 in a series of steps. We start by introducing some additional notation.

### 5.1 Additional notation

The vector of the values taken by the process on the rectangular grid $J_{n}$ is denoted $X={\left[X_{0},\ldots ,X_{|n|-1}\right]}^{T}$, where points are ordered into a vector according to the colexicographical order. Therefore in dimension *d* = 2, $X_{0},\ldots ,X_{n_{1}-1}$ are values from the first row of $J_{n}$, $X_{n_{1}},\ldots ,X_{2n_{1}-1}$ are values from the second row, and so on. Similarly we denote **g** the vector of the values taken by the modulation function on $J_{n}$, with points ordered in the same way. We also denote by $s_{0},\ldots ,s_{|n|-1}$ the locations of the grid ordered according to the same order, such that $X_{0}=X\left(s_{0}\right),X_{1}=X\left(s_{1}\right),$ etc.

We also denote by *G* the diagonal matrix with elements taken from **g**, such that the vector corresponding to the observed random field (rather than **X** which corresponds to the random field on the rectangular grid $J_{n}$) is given by the matrix product *G* **X**.

Finally, for any vector $v\in R^{p}$ we shall denote by ${\left\|v\right\|}_{q}$ its $L_{q}$ norm (in particular ${\left\|\cdot \right\|}_{2}$ is the Euclidean norm), and for any *p* × *p* matrix *A*, ‖*A*‖ shall denote the spectral norm, that is, the $L_{2}$‐induced norm,

(25) $$\left\|A\right\|=\max_{v\in R^{p},v\neq 0}\frac{{\left\|Av\right\|}_{2}}{{\left\|v\right\|}_{2}}.$$

We remind the reader that if *H* is a Hermitian matrix, since ${\left\|Hv\right\|}_{2}^{2}=v^{*}H^{*}Hv=v^{*}H^{2}v$, the spectral norm of *H* is its spectral radius, that is,

‖H‖=ρ(H)≡max{|λ|:λeigenvalue ofH}.

### 5.2 Distributional properties of the periodogram

It is well known for time series that the bias of the periodogram as an estimator of the spectral density is asymptotically zero (Koopmans, 1995). However, for spatial data in dimension *d* ≥ 2, the decay of the bias of the periodogram is known to be the dominant factor in terms of mean‐squared error (Dahlhaus & Künsch, 1987). Additionally, the bias is asymptotically zero under often non‐realistic assumptions, such as: full knowledge of the aliased spectral density, fully observed grid, growth of the domain in all directions. By directly fitting the expectation of the periodogram, rather than the spectral density, we circumvent this major pitfall of the Whittle likelihood for random fields. Having removed the effect of bias, we are left with studying the correlation properties of the periodogram. We show that the variance of a bounded linear combination of the periodogram at Fourier frequencies goes to zero. This is the result of Proposition 1, which we use later, in Lemma 15, to prove that if Assumption 1 holds our likelihood function converges uniformly in probability to its expectation.

Proposition 1 (Variance of linear functionals of the periodogram) *Suppose Assumption* 1 *holds and the random field is Gaussian. Let* $a_{k}\left(\omega \right)$ *be a family of functions with support* $T^{d}$, *indexed by* k∈N, *and uniformly bounded in absolute value. Then,*

(26) $$\mathrm{var}\left\{|n_{k}{|}^{-1}\sum_{\omega \in \Omega_{n_{k}}}a_{k}\left(\omega \right)I_{n_{k}}\left(\omega \right)\right\}=O\left\{\frac{\sum_{u\in Z^{d}}c_{g,k}\left(u\right)c_{X}^{2}\left(u\right)}{\sum g_{s}^{2}}\right\}.$$

Please see the Supplementary Material.

Corollary 1 (Extension to non‐Gaussian random fields) *Suppose Assumption* 1 *holds. Let* $a_{k}\left(\omega \right)$ *be a family of functions with support* $T^{d}$, *indexed by* k∈N, *and uniformly bounded in absolute value. Then*, *for non‐Gaussian random fields, the variance of linear combinations of the periodogram behaves according to*

(27) $$\mathrm{var}\left\{|n_{k}{|}^{-1}\sum_{\omega \in \Omega_{n_{k}}}a_{k}\left(\omega \right)I_{n_{k}}\left(\omega \right)\right\}=O\left\{\frac{\sum_{u\in Z^{d}}c_{g,k}\left(u\right)c_{X}^{2}\left(u\right)}{\sum g_{s}^{2}}+\frac{\left|n_{k}\right|}{{\left(\sum g_{s}^{2}\right)}^{2}}\right\}.$$

Please see the Supplementary Material.

In the non‐Gaussian case, the first requirement of SCC is adapted by accounting for the additional term in Equation (27) compared to Equation (26). If we observe a full rectangular grid with no tapering, then we have $\sum g_{s}^{2}=\left|n_{k}\right|$, the total number of points of the grid. If we assume square summability of the covariance function, then under the Gaussian assumption, the variance under study vanishes even if $\sum g_{s}^{2}={\left|n\right|}^{1/2}$. As we see with Equation (27), this may not hold anymore for non‐Gaussian data. One such example would be on a *d*‐rectangular grid. Assume we nominally sampled sides of length ℓ on a *d*‐dimensional cube. If we replace this by sampling $\Theta (\sqrt{\ell })$ points, leaving the rest as missing data then $\sum g_{s}^{2}={\left|n\right|}^{1/2}$, and convergence is no longer guaranteed in the non‐Gaussian case. If we no longer have a regularly sampled grid with some missing data, but a very complex spatial sampling then the DFT may not be the most convenient implementation, and we may adapt other methods, for example, Barnett et al. (2019). From Equation (27), however, we see that for non‐degenerate sampling scenarios, we can expect consistency of our estimator even for non‐Gaussian random fields.

Finally, for multivariate random fields, the same question arises about the variance of sesquilinear forms involving the elements of the vector‐Fourier transform. We present this as a second corollary to Proposition 1.

Corollary 2 (Extension to multivariate random fields) *Let* $\left\{A_{k}\left(\omega \right)\right\}$ *be a family of matrix‐valued functions with support* $T^{d}$, *indexed by* k∈N, *and uniformly bounded in terms of the maximum eigenvalues across all frequencies by* $\lambda_{\max}$. *If the random field is p*‐*multivariate Gaussian with absolutely summable cross‐covariance sequence, the variance of sesquilinear functionals of the discrete Fourier transform behaves according to*,

$$\mathrm{var}\left\{|n_{k}{|}^{‐1}\sum_{\omega \in \Omega_{n_{k}}}J_{n_{k}}^{*}\left(\omega \right)A_{k}\left(\omega \right)J_{n_{k}}\left(\omega \right)\right\}=O\left\{\sum_{q,r=1}^{p}\frac{\sum_{s}c_{g}^{\left(qr\right)}\left(s\right)c_{X}^{{\left(qr\right)}^{2}}\left(s\right)}{\sqrt{\sum_{s_{1}}g_{s_{1}}^{{\left(q\right)}^{2}}\sum_{s_{2}}g_{s_{2}}^{{\left(r\right)}^{2}}}}\right\}.$$

Please see the Supplementary Material.

### 5.3 Lemmata required for Theorem 1

All the lemmata in this section suppose that Assumption 1 holds. We provide all the proofs of this section in the Supplementary Material. To establish consistency we introduce some specific notation for the expectation of our quasi‐log‐likelihood,

(28) $${\tilde{\ell }}_{n}\left(\gamma \right)=E_{\theta }\left\{\ell_{n}\left(\gamma \right)\right\}={\left|n\right|}^{-1}\sum_{\omega \in \Omega_{n}}\left\{\log{\bar{I}}_{n}\left(\omega ;\gamma \right)+\frac{{\bar{I}}_{n}\left(\omega ;\theta \right)}{{\bar{I}}_{n}\left(\omega ;\gamma \right)}\right\},\;\forall n\in {\left(N^{+}\right)}^{d}\setminus \left\{0\right\},\forall \gamma \in \Theta ,$$

which we shall regard as a function of ***γ***. For multivariate random fields this is extended according to,

$${\tilde{\ell }}_{n}\left(\gamma \right)=E_{\theta }\{\ell_{n}\left(\gamma \right)\}={\left|n\right|}^{‐1}\sum_{\omega }\{\log\;\det\left\{\bar{I}\left(\omega ;\gamma \right)\right\}+\text{trace}\;[{\bar{I}}^{‐1}\left(\omega ;\gamma \right)\bar{I}\left(\omega ;\theta \right)]\}.$$

The following lemma relates the minimum of that function to the true parameter vector (with no uniqueness property as of now).

Lemma 9 (Minimum of the expected quasi‐likelihood function) *The expected likelihood function attains its minimum at the true parameter value, that is,*

(29) $${\tilde{\ell }}_{n}\left(\theta \right)=\min_{\gamma \in \Theta }{\tilde{\ell }}_{n}\left(\gamma \right).$$

We shall also make repeated use of the following lemma.

Lemma 10 (Lower and upper bounds on the expected periodogram) *The expected periodogram satisfies, for all parameter vector* ***γ*** ∈ Θ, *and at all wave numbers* $\omega \in T^{d}$, *for any* $n\in (N^{+}){\;}^{d}$,

$$f_{\delta ,\min}\leq {\bar{I}}_{n}\left(\omega ;\gamma \right)\leq f_{\delta ,\max}.$$

We now provide additional lemmata which are key to proving the consistency of our maximum quasi‐likelihood estimator. Lemma 11 states that the expected likelihood value at a parameter vector distinct from the true parameter value is asymptotically bounded away from the expected likelihood at the true parameter value. This comes as a consequence of the second part of SCC and the upper bound on the spectral densities of the model family.

Lemma 11 (Identifiability from the expected likelihood function) *Let* ***γ*** ∈ Θ *distinct from* ***θ***. *Then*,

(30) $${\underline{\lim}}_{k\to \infty }\;\left|{\tilde{\ell }}_{n_{k}}\left(\gamma \right)‐{\tilde{\ell }}_{n_{k}}\left(\theta \right)\right|>0,$$

*where* ${\underline{\lim}}_{k\to \infty }$ *denotes the limit inferior as k goes to infinity*.

For multivariate random fields, the proof of Lemma 11 requires an additional simple lemma,

Lemma 12 *Let* $H_{1},H_{2}$ *be two Hermitian positive definite Hermitian matrices*. *Then*,

(31) $$\text{trace}{\left[H_{1}H_{2}\right]}^{2}\geq {\left(\min\;\text{sp}\left(H_{1}\right)\right)}^{2}\text{trace}{\left[H_{2}\right]}^{2},$$

*where* $\text{sp}(H_{1})$ *denotes the set of eigenvalues of* $H_{1}$, *which are all positive*.

Lemma 13 now states a form of regularity of our expected likelihood functions. It relies on our regularity assumption on the spectral model family, where we have assumed the existence and boundedness of the partial derivatives with respect to the parameter vector (Assumption 1b).

Lemma 13 *Let* ***γ*** ∈ Θ *and let* ${\left(\gamma_{k}\right)}_{k\in N}$ *be a sequence of parameter vectors that converges to* ***γ***. *Then*,

(32) $${\tilde{\ell }}_{n_{k}}\left(\gamma_{k}\right)-{\tilde{\ell }}_{n_{k}}\left(\gamma \right)⟶0,\;\;\left(k⟶\infty \right).$$

Lemma 14 *Let* $\gamma_{k}\in \Theta^{N}$ *be a sequence of parameter vectors such that* ${\tilde{\ell }}_{n_{k}}\left(\gamma_{k}\right)-{\tilde{\ell }}_{n_{k}}\left(\theta \right)$ *converges to zero as k tends to infinity*. *Then* $\gamma_{k}$ *converges to* ***θ***.

And finally, the following lemma helps us understand how the likelihood function, as a random element, behaves with regard to the expected likelihood function.

Lemma 15 (Uniform convergence in probability of the likelihood function) *The log‐likelihood function* $\ell_{n_{k}}\left(\cdot \right)$ *converges uniformly in probability to* ${\tilde{\ell }}_{n_{k}}\left(\cdot \right)$ *over the parameter set* Θ *as k goes to infinity*.

With these lemmata we have all the necessary results to establish Theorem 1. This theorem is important as it establishes the consistency of our estimator under a very wide range of sampling schemes and model families. We contrast our results with those of Dahlhaus and Künsch (1987), Guyon (1982), as well as Fuentes (2007). The insight from Theorem 1, as compared to the insight of the need for tapering provided by Dahlhaus and Künsch (1987) is clear. The aim of this paper is to balance computational tractability with estimation performance. Very standard assumptions allow us to still derive the results required for estimation.

### 5.4 Convergence rate and asymptotic normality

We now study the convergence rate and asymptotic distribution of our estimates within the increasing‐domain asymptotics framework. In Theorem 2 we establish a convergence rate in the general framework of HSCC (Definition 1) for both Gaussian and non‐Gaussian random fields, and we also establish asymptotic normality in the scenario of a Gaussian random field observed on a full grid. Under further requirements (Assumption 3), asymptotic normality is shown for non‐Gaussian random fields in Theorem 3, together with a limiting form of the covariance structure of our estimator.

To prove our theorems, we first need to understand better the behaviour of quantities of the form ${\left|n\right|}^{-1}\sum_{\omega \in \Omega_{n_{k}}}w_{k}\left(\omega \right)I_{n}\left(\omega \right)$, for some weights $w_{k}$. In Proposition 1, we had already showed that under mild conditions, their variance vanished at a rate driven by the number of observed points. Now in Proposition 2, and under the assumption of a full grid, by writing this quantity as a quadratic form in the random vector **X** and extending a result by Grenander and Szegö (1958), we show that this quantity is asymptotically normally distributed, under mild conditions on the family of functions $w_{k}\left(\cdot \right)$. Before getting there, we need the following intermediary result, which extends a standard result for Toeplitz matrices to their multi‐dimensional counterpart, Block Toeplitz with Toeplitz Block matrices.

Lemma 16 (Upper bound on the spectral norm of the covariance matrix) *Suppose Assumption* 1 *holds*. *In the case of a full grid, the spectral norm of* $C_{X}$ *and that of its inverse are upper bounded according to*

$$\|C_{X}\|\leq f_{\delta ,\max},\left\|C_{X}^{-1}\right\|\leq f_{\delta ,\min}^{-1}.$$

Please see the Supplementary Material.

Proposition 2 (Asymptotic normality of linear combinations of the periodogram) *Suppose Assumption* 1 *holds and that the random field is Gaussian and observed on a full grid. Let* $w_{k}\left(\cdot \right),k\in N$ *be a family of real‐valued functions defined on* $T^{d}$ *bounded above and below by two constants, denoted* $M_{W},\;m_{W}>0$ *respectively. Then* ${\left|n\right|}^{-1}\sum_{\omega \in \Omega_{n_{k}}}w_{k}\left(\omega \right)I_{n}\left(\omega \right)$ *is asymptotically normally distributed*.

Please see the Supplementary Material.

Before finally establishing our convergence rates, as well as the asymptotic normality in the case of a Gaussian random field observed on a full grid, we require one additional set of assumptions.

Assumption 2 (Assumptions for convergence rate and asymptotic normality)

a. The interior of Θ is non‐null and the true length‐*p* parameter vector ***θ*** lies in the interior of Θ.
b. The sequence of observation grids leads to HSCC for the considered model family.

The following lemma relates HSCC to the minimum eigenvalue of the expectation of the Hessian matrix of *l*(·) at the true parameter vector.

Lemma 17 *Under HSCC, the minimum eigenvalue of the expectation of the Hessian matrix (with respect to the parameter vector) at the true parameter, given by*

(33) $$\left(|n_{k}{|}^{-1}\sum_{\omega \in \Omega_{n_{k}}}{\bar{I}}_{n_{k}}{\left(\omega ;\theta \right)}^{-2}\nabla_{\theta }{\bar{I}}_{n_{k}}\left(\omega ;\theta \right)\nabla_{\theta }^{T}{\bar{I}}_{n_{k}}\left(\omega ;\theta \right)\right),$$

*is lower bounded by S*(***θ***), *which was defined in Definition* 2.

This can be established by a direct adaptation of lemma 7 of Guillaumin et al. (2017).

Theorem 2 (Convergence rate and asymptotic normality of estimates) *Suppose Assumptions* 1 *and* 2 *hold*. *Our estimate converges in probability with rate*

$$r_{k}={\left(\frac{\sum_{u\in Z^{d}}c_{g,k}\left(u\right)c_{X}^{2}\left(u\right)}{\sum g_{s}^{2}}+\frac{\left|n_{k}\right|}{{\left(\sum g_{s}^{2}\right)}^{2}}\right)}^{1/2}.$$

*If the random field is Gaussian the convergence rate simplifies to,*

$$r_{k}={\left(\frac{\sum_{u\in Z^{d}}c_{g,k}\left(u\right)c_{X}^{2}\left(u\right)}{\sum g_{s}^{2}}\right)}^{1/2}.$$

*In addition, if the grid is fully observed and the random field is Gaussian, then* $\hat{\theta }$ *is asymptotically normally distributed*.

Please see the Supplementary Material.

Note that in Theorem 2 we do not make assumptions about the dimensions of the observation domain, as is usually the case for Whittle‐type estimators where a common growth rate in all directions is typically assumed. Asymptotic normality of our estimate can also be established for non‐Gaussian random fields under appropriate assumptions on high‐order cumulants, which we introduce below.

Assumption 3

a. *Observation domain*. The grid is fully observed, and we set $g_{s}=1$ on the grid and 0 otherwise. Additionally, we require the domain to be unbounded in all directions for asymptotic forms to hold.
b. *Higher‐order homogeneity*. Joint moments of any order are finite and for any positive integer *L* ≥ 2 and locations $s_{1},\ldots ,s_{L}\in R^{d}$, for any $u\in R^{d}$, $\text{cum}[X_{s_{1}},\ldots ,X_{s_{L}}]=\text{cum}[X_{s_{1}+u},\ldots ,X_{s_{L}+u}]$. If this assumption holds we define for $u_{1},\ldots ,u_{L-1}\in R^{d}$,
(34) $$c_{L}\left(u_{1},\ldots ,u_{L-1}\right)=\text{cum}\left[X_{s_{0}},X_{s_{0}+u_{1}},\ldots ,X_{s_{0}+u_{L-1}}\right],\forall s_{0}\in R^{d}.$$
  In particular $c_{2}\left(\cdot \right)$ is just the autocovariance function of the random field.
c. *Short‐length memory*. For any positive integer *L* ≥ 2,
(35) $$\sum_{u_{1},\ldots ,u_{L-1}\in R^{d}}\left(1+\|u_{j}{\|}^{d}\right)\left|c_{L}\left(u_{1},\ldots ,u_{L-1}\right)\right|<\infty ,\;j=1,\ldots ,d.$$

Proposition 3 *Suppose Assumptions* 1 *and* 3 *hold. Let* $w_{k}\left(\cdot \right)$ *be uniformely bounded vector‐valued functions from* $T^{d}$ *to* $R^{d}$ *such that* $\left\{w_{k}\left(\cdot \right)\right\}$ *converges to* **w**(·) *pointwise, where* **w**(·) *is a Rieman‐integrable function with values in* $R^{d}$. *Then,* ${\left|n\right|}^{-1}\sum_{\omega \in \Omega_{n}}w_{k}\left(\omega \right)I_{n}\left(\omega \right)$ *is asymptotically jointly normal. Additionally, suppose the grid grows to infinity in all directions, the asymptotic covariance structure of* ${\left|n\right|}^{-1}\sum_{\omega \in \Omega_{n}}w_{k}\left(\omega \right)I_{n}\left(\omega \right)$ *is then determined by*

$$\begin{array}{cc} & {\left(2\pi \right)}^{d}|n{|}^{-1}\int_{T^{d}}{(w(\omega )+w(-\omega ))w}^{T}(\omega )f_{X,\delta }{\left(\omega \right)}^{2}d\omega \\ & \;+{\left(2\pi \right)}^{d}|n{|}^{-1}\int_{T^{d}}\int_{T^{d}}w(\omega_{1})w^{T}(\omega_{2})f_{X,4,\delta }(\omega_{1},\omega_{2},-\omega_{1})d\omega_{1}d\omega_{2}, \end{array}$$

*where* $f_{X,4,\delta }\left(\cdot ,\cdot ,\cdot \right)$ *is the fourth‐order cumulant spectral density, that is*,

$$f_{X,4,\delta }\left(\omega_{1},\omega_{2},\omega_{3}\right)=\sum_{u_{1},u_{2},u_{3}}c_{4}\left(u_{1},u_{2},u_{3}\right)e^{-i(u_{1}\cdot \omega_{1}+u_{2}\cdot \omega_{2}+u_{3}\cdot \omega_{3})},$$

*and where* **w**(−***ω***) *is obtained by 2π periodic extension of* **w** *along all dimensions*.

Please see the Supplementary Material.

Proposition 3 is similar to Proposition 2. The two differ in terms of the assumptions required to prove the result. Proposition 2 requires the random field to be Gaussian while Proposition 3 allows for non‐Gaussian random fields at the expense of additional constraints on the memory of the random field.

Theorem 3 *Suppose Assumptions* 1, 2–3 *hold. Then* $\hat{\theta }$ *is asymptotically normally distributed. Additionally, if the observed random field is Gaussian and the observation domain grows to infinity in all directions*, $\hat{\theta }$ *admits an asymptotic covariance structure determined by*,

$$2^{d+1}\pi^{d}{\left|n\right|}^{-1}{\left[\int_{T^{d}}\nabla_{\theta }\log f_{X,\delta }\left(\omega ;\theta \right)\nabla_{\theta }^{T}\log f_{X,\delta }(\omega ;\theta )d\omega \right]}^{-1}.$$

This results from combining Proposition 3 and the proof of Theorem 2.

The asymptotic form of the covariance structure can also be determined for the non‐Gaussian case from Proposition 3. Theorem 3 is a generalization of a standard result in time series analysis (Brockwell & Davis, 2009, theorem 10.8.2). However, see for example, Simons and Olhede (2013) for a practical large‐sample example where the asymptotic form has not been reached, but is instead dependent on the true form of the expected periodogram as well as the sample size. This—in addition to scenarios of incomplete grids—motivates the following section, where we consider estimation of standard errors in the more general setting where our asymptotic results do not hold.

### 5.5 Estimating standard errors

We now seek to derive how to estimate the standard error of $\hat{\theta }$ for a given spatial sampling and model family. Using Equations (20) and (21) from the Supplementary Material, we obtain an approximation for the variance of $\hat{\theta }$ in the following proposition, where H denotes the Fisher Information matrix.

Proposition 4 (Form of the variance) *The covariance matrix of the quasi‐likelihood estimator takes the form of*

(36) $$\mathrm{var}\left\{\hat{\theta }\right\}\approx {ℋ}^{-1}\left(\theta \right)\mathrm{var}\left\{\nabla \ell_{M}\left(\theta \right)\right\}{ℋ}^{-1}\left(\theta \right),$$

*with the covariance matrix of the score taking the form of*

(37) $$\text{cov}\left\{\frac{\partial \ell_{M}\left(\theta \right)}{\partial \theta_{p}},\frac{\partial \ell_{M}\left(\theta \right)}{\partial \theta_{q}}\right\}={\left|n\right|}^{-2}\sum_{\omega_{1},\omega_{2}\in \Omega_{n}}\frac{\text{cov}\left\{I_{n}\left(\omega_{1}\right),I_{n}\left(\omega_{2}\right)\right\}}{{\bar{I}}_{n}^{2}\left(\omega_{1};\theta \right){\bar{I}}_{n}^{2}\left(\omega_{2};\theta \right)}\frac{\partial {\bar{I}}_{n}\left(\omega_{1};\theta \right)}{\partial \theta_{p}}\frac{\partial {\bar{I}}_{n}\left(\omega_{2};\theta \right)}{\partial \theta_{q}}.$$

The computation that appears in Equation (37) scales like ${\left|n\right|}^{2}$, that is, not well for large grid sizes. We instead propose a Monte Carlo implementation to speed this up. The dominant terms in Equation (37) correspond to $\omega_{1}=\omega_{2}$. We approximate the sum over the rest of the terms, in the form

$$\begin{array}{cc} & \text{cov}\left\{\frac{\partial \ell_{M}\left(\theta \right)}{\partial \theta_{p}},\frac{\partial \ell_{M}\left(\theta \right)}{\partial \theta_{q}}\right\}={\left|n\right|}^{-2}\sum_{\omega_{1}\in \Omega_{n}}\left\{\frac{\partial {\bar{I}}_{n}\left(\omega_{1};\theta \right)}{\partial \theta_{p}}\frac{\mathrm{var}\left\{I_{n}\left(\omega_{1}\right)\right\}}{{\bar{I}}_{n}^{4}\left(\omega_{1};\theta \right)}\frac{\partial {\bar{I}}_{n}\left(\omega_{1};\theta \right)}{\partial \theta_{q}}\right\}\;\\ & \;\;+\frac{{\left|n\right|}^{2}-\left|n\right|}{{M|n|}^{2}}\sum_{i=1\ldots M}\frac{\partial {\bar{I}}_{n}\left(\omega_{1,i};\theta \right)}{\partial \theta_{p}}\frac{\text{cov}\left\{I_{n}\left(\omega_{1,i}\right),I_{n}\left(\omega_{2,i}\right)\right\}}{{\bar{I}}_{n}^{2}\left(\omega_{1,i};\theta \right){\bar{I}}_{n}^{2}\left(\omega_{2,i};\theta \right)}\frac{\partial {\bar{I}}_{n}\left(\omega_{2,i};\theta \right)}{\partial \theta_{q}}, \end{array}$$

where the $\omega_{1,i},\omega_{2,i},i=1\ldots M$ are uniformly and independently sampled from the set of Fourier frequencies $\Omega_{n}$ under the requirement $\omega_{1,i}\neq \omega_{2,i}$. Note that if tapering is used, one should consider a few coefficients near the main diagonal in the above approximation, as tapering generates strong short‐range correlation in the frequency domain.

The covariances of the periodogram at two distinct Fourier frequencies can be approximated by Riemann approximation of the two integrals that appear in the expression below, before taking squared absolute values and summing,

$$\begin{array}{cc} \text{cov}\left\{I_{n}\left(\omega_{1,i}\right),I_{n}\left(\omega_{2,i}\right)\right\} & ={\left|n\right|}^{-1}\left({\left|\int_{T^{d}}\tilde{f}\left(\lambda \right)D_{n}\left(\lambda -\omega_{1,i}\right)D_{n}^{*}\left(\lambda -\omega_{2,i}\right)d\lambda \right|}^{2}\right.\\ & \;+\left.{\left|\int_{T^{d}}\tilde{f}\left(\lambda \right)D_{n}\left(\lambda -\omega_{1,i}\right)D_{n}^{*}\left(\lambda +\omega_{2,i}\right)d\lambda \right|}^{2}\right),\;i=1,\ldots ,M. \end{array}$$

In the above, $\tilde{f}$ is the following approximation to the spectral density, which can be computed by a DFT,

$$\tilde{f}\left(\lambda \right)=\sum_{u\in \prod_{i=1}^{d}\left[-\left(n_{i}-1\right)\ldots \left(n_{i}-1\right)\right]}c_{X}\left(u;\theta \right)\exp\left(-i\lambda \cdot u\right),$$

and $D_{n}\left(\lambda \right)$ is the non‐centred *modified* (due to the modulation $g_{s}$) Dirichlet kernel of order **n** given by

$$D_{n}\left(\lambda \right)=\sum_{s\in J_{n}}g_{s}\exp\left(i\lambda \cdot s\right),$$

where for clarity we omit the dependence on the modulation $g_{s}$ in the notation. Finally we compute the derivatives of ${\bar{I}}_{n}\left(\omega ;\theta \right)$ as follows,

(38) $$\nabla_{\theta }{\bar{I}}_{n}\left(\omega ;\theta \right)=\sum_{u\in Z^{d}}\nabla_{\theta }{\bar{c}}_{X}\left(u;\theta \right)\exp\left(-i\omega \cdot u\right).$$

## 6 SIMULATION STUDIES AND APPLICATION TO THE STUDY OF PLANETARY TOPOGRAPHY

In this section we present simulation studies and an application to the study of Venus' topography that demonstrate the performance of the Debiased Spatial Whittle estimator. We also refer the reader to the Supplementary Material which contains additional simulation studies. The simulations presented in Section 6.1 address the estimation of the range parameter of a Matérn process, whose slope parameter is known, observed over a full rectangular grid. These simulations corroborate our theoretical results on the optimal convergence rate of our estimator despite edge effects, in contrast to the standard Whittle method. Our second simulation study in Section 6.2 shows how our estimation procedure extends the computational benefits of frequency‐domain methods to non‐rectangular shapes of data, where we compare parameter estimates with those of Guinness and Fuentes (2017) in the scenario of a circular shape of observations. In Section 6.3 we estimate the parameters of a simulated Matérn process sampled according to a real‐world sampling scheme of terrestrial ocean‐floor topography (GEBCO Bathymetric Compilation Group, 2019) with approximately 72% missing data. Finally, in Section 6.4 we demonstrate the performance of the Debiased Spatial Whittle estimator when applied to topographical data sets obtained from Venus (Rappaport et al., 1999).

### 6.1 Estimation from a fully observed rectangular grid of data

We simulate from the isotropic Matérn model family, which corresponds to the following covariance function,

(39) $$c_{X}\left(u\right)=\sigma^{2}\frac{2^{1-\nu }}{\Gamma (\nu )}{\left(\sqrt{2\nu }\frac{\left\|u\right\|}{\rho }\right)}^{\nu }K_{\nu }\left(\sqrt{2\nu }\frac{\left\|u\right\|}{\rho }\right),$$

where $K_{\nu }\left(x\right)$ is a Bessel function of the second kind. We consider the problem of estimating the range parameter *ρ*, which is fixed to 10 units, while the amplitude $\sigma^{2}=1$ and the slope parameter $\nu \in \{\frac{1}{2},\frac{3}{2}\}$ are fixed and known. Inference is achieved from simulated data on two‐dimensional rectangular grids of increasing sizes, specifically $\left\{2^{s}:s=4,\cdots ,8\right\}$ in each dimension. We implement four inference methods:

M1. The Debiased Spatial Whittle method, that is, the estimate derived from Equation (7);
M2. The Debiased Spatial Whittle method combined with a taper, specifically the estimate derived from Equation (7) with $g_{s}$ proportional to a Hanning taper;
M3. The standard Whittle likelihood, that is, estimators obtained by replacing ${\bar{I}}_{n}\left(\omega ;\theta \right)$ with $f_{X}\left(\omega \right)$ in Equation (5) and then minimizing Equation (7);
M4. The standard Whittle likelihood combined with tapering using a Hanning taper, again derived from Equation (7) fitting to $f_{X}\left(\omega \right)$.

**FIGURE 1:**
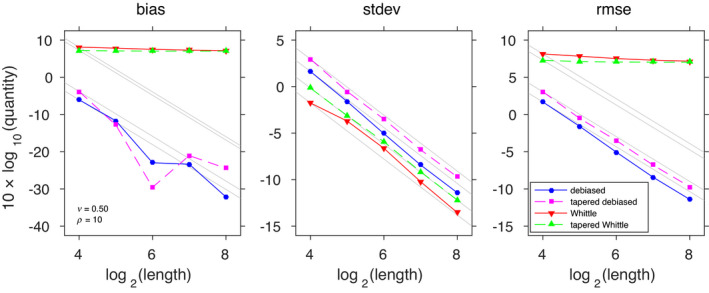
Bias, standard deviation, and root mean‐squared error of estimates of the range parameter *ρ* = 10 of a Matérn process (39) with $\nu =1/2,\sigma^{2}=1$. The estimation method is identified by the line style, and grey lines functionally express the theoretical dependence on the square root of the sample size. The side length of the two‐dimensional square grid is indicated by the horizontal axis, leading to a sample size of the length squared [Colour figure can be viewed at wileyonlinelibrary.com]

**FIGURE 2:**
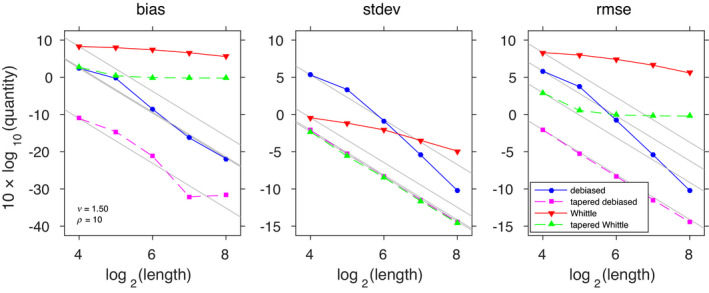
The same simulation setup as in Figure 1, but with *ν* = 3/2. This higher slope parameter is associated with smoother realizations, resulting in worsened edge effects. This illustrates how our method effectively addresses the edge effect issues even in that setting [Colour figure can be viewed at wileyonlinelibrary.com]

For each configuration of the slope parameter and grid size, we report summary statistics corresponding to 1000 independently realized random fields. We report bias, standard deviation and root mean‐squared error for *ν* = 1/2 and *ν* = 3/2 in Figures 1 and 2, respectively.

We first observe that the rate of the Whittle likelihood (M3) is very poor, due to its large bias. It appears that tapering (M4) leads to improved convergence rates when *ν* = 3/2, although bias remains. In contrast, the rates of our proposed method (M1) and its tapered version (M2) do not curb down even with larger grid sizes. This concurs with the theoretical results on the rate of convergence provided in Section 5. This example demonstrates that the Debiased Spatial Whittle method balances the need for computational and statistical efficiency with large data sets.

**FIGURE 3:**
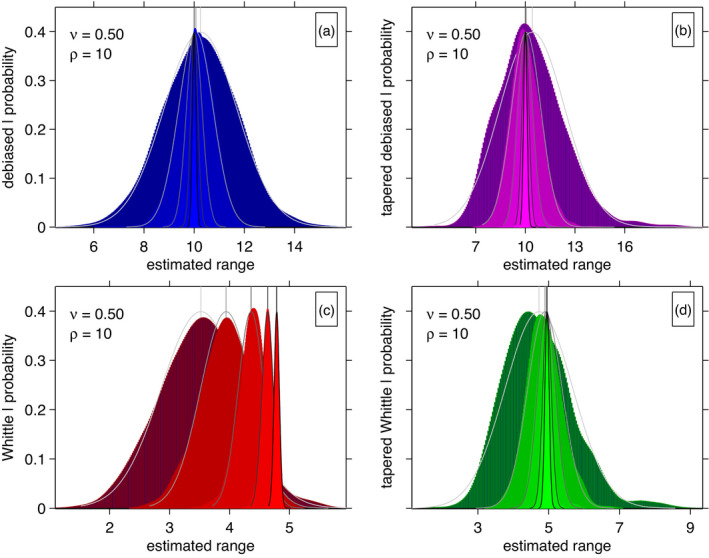
Nonparametric density estimates $\hat{\rho }$ of the estimated range parameter $\hat{\rho }$ (*ρ* = 10) for a Matérn random field (39), with $\sigma^{2}=1$ and *ν* = 1/2. The four subplots show different estimation methods of (a) Debiased Spatial Whittle, (b) Debiased Spatial Whittle with tapering, (c) standard Whittle and (d) standard Whittle with tapering. The density estimate is shaded to reflect the size of the random field, with the darkest corresponding to total observations $|n|\;={\left(2^{4}\right)}^{2}$, and the shading incrementally taking a lighter colour for $\left|n\right|={\left(2^{5}\right)}^{2},{\left(2^{6}\right)}^{2},{\left(2^{7}\right)}^{2},{\left(2^{8}\right)}^{2}$. Each density estimate is complemented by the best fitting Gaussian approximation as a solid black or fading grey line (black corresponds to $\left|n\right|={\left(2^{8}\right)}^{2}$ and the lightest grey to $\left|n\right|={\left(2^{4}\right)}^{2}$) [Colour figure can be viewed at wileyonlinelibrary.com]

In Figure 3 we report the empirical distribution of each estimator obtained from the 1000 independent inference procedures for *ν* = 1/2. The four panels (a), (b), (c) and (d) show the distribution of estimates from the four methods. The first two panels, (a) and (b), are broadly unbiased with estimates centred on *ρ* = 10 that converge quickly. The standard Whittle method (c) has issues with underestimation, tending towards *ρ* = 5. This asymptotic bias is in large part due to aliasing not being accounted for, combined with the relatively small value of *ν* = 1/2; these effects are still present in the tapered estimates (d). As would be expected, in all four subplots the variance is decreasing with increasing sample size, at similar rates. In the Supplementary Material we present the same study where the Whittle and tapered Whittle methods use an aliased version of the spectral density. This largely reduces the bias of these methods. However, some asymptotic bias remains, even for the tapered Whittle method, due to our fixed approximation to the aliased spectral density owing to computational constraints.

### 6.2 Estimation from a circular set of observations

In this section, we show how our Debiased Spatial Whittle method extends to non‐rectangular data. More specifically, we assume we only observe data within a circle with diameter 97 units. We consider the exponential covariance kernel given by

(40) $$c_{X}\left(u\right)=\sigma^{2}\exp\left(-\frac{\left\|u\right\|}{\rho }\right),\;u\in R^{2},$$

where $\sigma^{2}=1$ is fixed and known and we estimate the range parameter *ρ* whose true value is set to 5 units. We note that the case of a growing circle satisfies SCC, according to Lemma 7, and hence leads to consistency of our estimator. We also expect optimal convergence rates, see Theorem 2.

A total number of 1200 independent simulations are performed. As a state‐of‐the‐art baseline, we compare to a recent method proposed by Guinness and Fuentes (2017), which is an approximation of the circulant embedding method developed by Stroud et al. (2017). These authors proposed an Expectation Maximization iterative procedure, where the observed sample is embedded onto a larger grid that makes the covariance matrix *Block Circulant with Circulant Blocks* (BCCB), which can be diagonalized fast through the FFT algorithm. Guinness and Fuentes (2017) point out that the size of the embedding grid is very large, making the imputations costly and the convergence over the iterations slow. To address this limitation they propose using a periodic approximation of the covariance function on an embedding grid which is much smaller than that required for the exact procedure. They show via simulations that using an embedding grid ratio of 1.25 along each axis leads to good approximations of the covariance function on the observed grid.

To implement the method developed by Guinness and Fuentes (2017), we use the code provided by the authors. We set a grid ratio of 1.25 to limit the computational cost, and implement the method with two choices of the number of imputations per iteration, *M* = 1 and *M* = 20. Each implementation is run for a number of 30 iterations for all samples.

**FIGURE 4:**
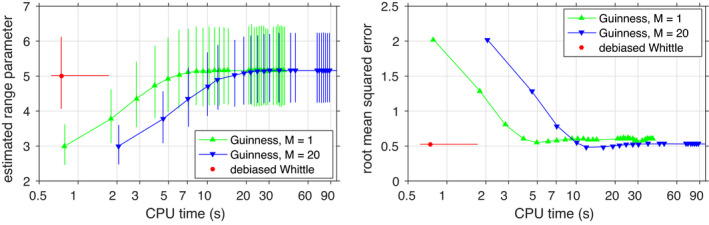
Mean and 95% confidence intervals (left) and root mean‐squared error (right) of estimates of the range parameter *ρ* = 5 of an exponential covariance model (40). Estimation is performed on a circular set of data with diameter 97 units. The converged estimates of the Debiased Spatial Whittle method are compared to the iterated estimates of two implementations of Guinness and Fuentes (2017). The horizontal axis in both panels corresponds to the average computational time, as performed on an Intel(R) Core(TM) i7‐7500U CPU 2.7–2.9GHz processor [Colour figure can be viewed at wileyonlinelibrary.com]

Both our estimation method and that of Guinness and Fuentes (2017) are initialized with the estimates provided by the method proposed by Fuentes (2007). We show in Figure 4 (left) how the Debiased Spatial Whittle method achieves computational and statistical efficiency. The 95% confidence interval of our estimate is similar to that obtained via the method of Guinness and Fuentes (2017) (*M* = 1), however, our method, despite also using an iterative maximization procedure, is significantly faster. As shown in Figure 4 (right panel), Guinness and Fuentes (2017) (*M* = 20) leads to lower root mean‐squared error but requires more computational time.

### 6.3 Application to a realistic sampling scheme of ocean‐floor topography

**FIGURE 5:**
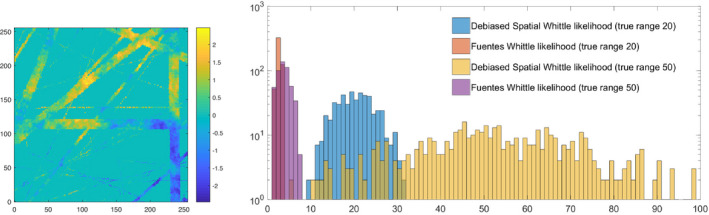
(Left) Simulated Matérn process with slope parameter 0.5 and range parameter 50 units, on a real‐world sampling grid, with missing observations replaced by zeros. (Right) Histogram of estimates of the range parameter of a simulated Matérn process observed on the real‐world grid shown in the left panel. We compare our proposed estimation method, the Debiased Spatial Whittle likelihood, to the method proposed by Fuentes (2007). The true value of the range is fixed to 20 or 50. Despite an increased variance due to the complex missing data patterns, our method is still able to produce a useful estimate of the range parameter, in comparison to the estimates produced by the method proposed by Fuentes (2007), which was not built to address such large and complex patterns of missing data [Colour figure can be viewed at wileyonlinelibrary.com]

In this simulation study we show that our estimator can address complex lower‐dimensional sampling substructure. We apply it to the estimation of a Matérn process sampled on a real‐world observation grid of ocean‐bathymetry soundings, characterized by a very large amount of missing data (≈72%). We simulate two Matérn processes, each with slope parameter 0.5 and with range 20 and 50 units respectively. The initial grid is of size 1081 × 1081. We select a subgrid of size 256 × 256 with similar missingness properties to those of the whole grid. In Figure 5 we plot (left) a simulated Matérn process on that grid where missing observations have been replaced with zeros. We note the large amount of missing observations within the bounding rectangular grid, as well as its complex patterns (i.e. rather than a uniform missingness scheme). For both these reasons the method proposed by Fuentes (2007) fails, while our method is still able to produce useful estimates, as shown in the right panel of Figure 5.

### 6.4 Application to the study of Venus' topography

In this section we apply our Debiased Spatial Whittle method to the study of Venus' topography. The motivation for modelling a planet's topography using a parametric covariance model such as the Matérn process is multifaceted. For instance, we may expect that the combination of the slope and range parameters will carry important information about the geomorphological process or age of formation of the observed topography, that is, it is expected that those parameters will have an interpretable physical meaning. The slope parameter can be related to the smoothness of the topography, and the range parameter tells about the typical distance over which two observed portions are uncorrelated.

**FIGURE 6:**
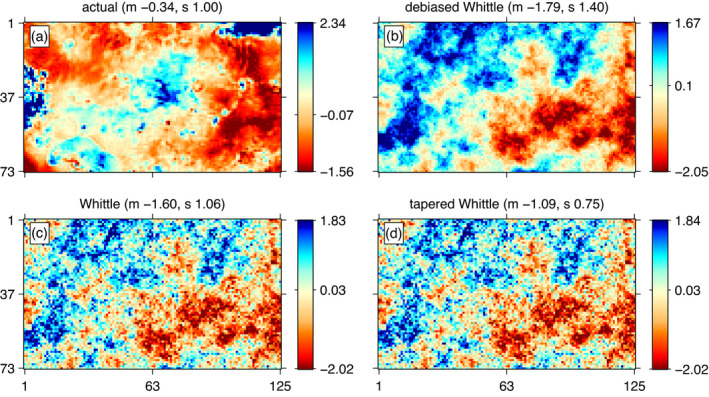
(a) A realized random field from the topography of Venus; and simulated random fields from a Matérn model with parameters estimated using (b) Debiased Spatial Whittle estimation, (c) standard Whittle estimation and (d) standard Whittle estimation using a Hanning taper. Simulated random fields were obtained using the same random seed to facilitate comparison. Parameter values for each method are given in Table 1 (Patch 3) in Section 6.4. Sample means (m) and standard deviations (s) are in the titles. Colour bars are marked at the 2.5th, 50th and 97.5th quantiles. Axis labels are in pixels [Colour figure can be viewed at wileyonlinelibrary.com]

Building on the work of Eggers (2013), we have selected four patches of data (including that shown in Figure 6 which corresponds to Patch 3), each sampled regularly on a complete rectangular grid. We compare three estimation procedures: the Debiased Spatial Whittle method, the standard Whittle method, and the standard Whittle method with tapering (again using a Hanning taper). Parameter estimates are reported in Table 1. We also compare the value of the exact likelihood function taken at the estimated parameters for each estimation method in Table 2. Specifically, if ${\hat{\theta }}_{M}$ and ${\hat{\theta }}_{W}$ respectively denote the estimates obtained via the Debiased Spatial Whittle and standard Whittle procedure, we compare $l_{E}\left({\hat{\theta }}_{M}\right)$ and $l_{E}\left({\hat{\theta }}_{W}\right)$, with $l_{E}\left(\cdot \right)$ denoting the exact likelihood function (which is expensive to evaluate but only needs to be done once for each analysed method). The results in Table 2 show a much better fit of the model corresponding to the parameters estimated via the Debiased Spatial Whittle method, in comparison to the parameters estimated via either standard Whittle or tapered Whittle. The parameter estimates in Table 1 should be interpreted with care due to the challenges inherent in joint estimation of all three parameters of a Matérn covariance function (see, e.g. Zhang, 2004). However, in all four patches we observe that the standard and tapered Whittle likelihood appear to overestimate the range while underestimating the smoothness, consistent with results found by Sykulski et al. (2019) for oceanographic time series.

**TABLE 1:** Estimates of the three parameters of a Matérn process, see Equation (39)

	Patch 1			Patch 2			Patch 3			Patch 4		
Parameter:	*σ*	*ν*	*ρ*	*σ*	*ν*	*ρ*	*σ*	*ν*	*ρ*	*σ*	*ν*	*ρ*
Debiased Spatial Whittle	1.2	0.5	17.7	1.2	0.7	6.8	2.1	0.5	36.5	1.5	0.6	15.0
Standard Whittle	1.6	0.3	62.7	1.8	0.3	73.9	1.5	0.2	77.3	1.7	0.3	87.3
Tapered Whittle	2.0	0.4	52.0	1.7	0.2	80.6	1.2	0.2	88.1	1.9	0.4	83.7

**TABLE 2:** Percentage of increase in the exact likelihood value at the estimated parameter values from Table 1 in comparison to the minimal value obtained among the three methods

	Patch 1	Patch 2	Patch 3	Patch 4
Debiased Spatial Whittle	60.60	104.80	91.60	48.40
Standard Whittle	0	16.10	0	0
Tapered Whittle	23.20	0	53.90	25.20

Finally, Figure 6 presents a comparison of Patch 3 with three simulated samples, obtained using the Matérn model estimated using the Debiased Spatial Whittle, standard and tapered Whittle methods respectively. This analysis supports the conclusion that the Debiased Spatial Whittle method is able to find more appropriate parameter values for the model fit.

## 7 DISCUSSION

In this paper we addressed the estimation of parametric covariance models for Gaussian and non‐Gaussian random fields using the discrete Fourier transform. Key to understanding a random field is its spatial sampling; this can range from a spatial point process, to regular sampling with an irregular boundary, to observations missing at random on a grid, to a fully sampled square regular grid. To maintain computational feasibility, this paper addresses the analysis of a regularly sampled random field, with potentially missing observations and an irregular (not cuboid) sampling domain.

The Whittle likelihood uses the FFT to achieve computational efficiency. The approximation is based on results for Block Toeplitz with Toeplitz Blocks matrices (Kazeev et al., 2013; Tyrtyshnikov & Zamarashkin, 1998), on (growing‐domain) asymptotics, and on arguments that equate the Gaussian non‐diagonal quadratic form with another Gaussian, nearly diagonal, form. For time series this argument is relatively straightforward, but is somewhat more complex for spatial data in higher dimensions, where the bias becomes the dominant term (Guyon, 1982), and the geometry of the sampling process leaves a strong imprint.

The bias of the periodogram as an estimator of the spectral density (which drives subsequent bias) decreases with rate $O\left({\left|n\right|}^{-1/d}\right)$ (Dahlhaus & Künsch, 1987; Guyon, 1982) in the ideal case of a fully observed rectangular lattice in *d* dimensions that grows at the same rate along all directions. Dahlhaus (1983) proposed tapering to remedy this issue. A more general result by Kent and Mardia (1996) shows that the approximation resulting from replacing the exact likelihood with the Whittle likelihood in the case of a full grid is driven by the size of the smallest side of the rectangular lattice. Tapering on its own cannot solve this issue. To address bias in a general setting we proposed replacing the spectral density by the true expectation of the periodogram. From the notion of SCC, we can understand the technical underpinning of this bias removal process and draw a general framework of sampling schemes and model families for which our estimator is statistically efficient.

In addition, our Debiased Whittle procedure also explicitly accounts for aliasing in the computation of the expected periodogram, thus avoiding computationally‐expensive wrapping operations to fold in higher unobserved frequencies into the likelihood. As would be expected, in simulations we found the bias correction from aliasing to be most important when the rate of decay in the spectral density in frequency is slow (e.g. a Matérn process with small slope parameter). In contrast, we found that accounting for finite sampling and boundary effects to be most important when the rate of decay is high and the spectrum therefore has a large dynamic range (e.g. a Matérn process with large slope parameter). Overall, our explicit handling for the effects of missing data provided further improvements for all processes studied, regardless of the specific form of the spectral density.

For random fields with missing observations, Fuentes (2007) suggested to replace the missing points of a rectangular lattice with zeros, as we do in Equation (4), and correcting uniformly across frequencies for the amplitude of the periodogram, based on the ratio of the number of observed points to the total number of points in the grid. This only partly corrects for the bias of the periodogram that results from any non‐trivial shape of the data, as frequencies are likely to not be affected uniformly by the sampling scheme; in contrast to our estimation procedure which directly encodes the observed data, and the observed missingness pattern. Under relatively weak assumptions, and through the notion of SCC, we establish consistency and asymptotic normality in both Gaussian and non‐Gaussian settings.

When studying non‐Gaussian observations one can take two approaches; either limiting the effects of the non‐Gaussianity on the variance of the estimator (Giraitis & Taqqu, 1999; Sykulski et al., 2019), or even permitting Whittle‐type estimation based on higher order spectral moments, see e.g. Anh et al. (2007). If infill asymptotics are considered (Bandyopadhyay & Lahiri, 2009), then the limiting distribution of the Fourier transform need not be Gaussian. Note that the aforementioned authors assumed completely random sampling of the fields, which we do not, as such sampling leads to a ‘nugget effect’ at frequency zero and beyond.

To treat more general multivariate processes, we defined a multivariate sampling mechanism that is initially on the same grid, but where the missingness pattern may be different between processes. To be able to arrive at consistent estimators, we again use a version of the concept of SCC, but now adapted to the multivariate nature of the data. Under this assumption, which ensures we gain more information as our sampling scheme diverges in cardinality, we do achieve estimation consistency.

Stroud et al. (2017) have proposed an approach that does not require approximating the multi‐level Toeplitz covariance matrix of the rectangular lattice sample by a multi‐level circulant matrix. Instead, their method finds a larger lattice, termed an embedding, such that there exists a BCCB matrix that is the covariance matrix of a Gaussian process on this extended lattice, and such that the covariance matrix of the real process is a submatrix of this extended matrix. One can then simulate efficiently the missing data on the extended lattice, and estimate the parameters of the models. This process can be iterated until a convergence criterion is met. This elegant method still suffers from computational issues, as the size of the embedding might be quite large. A solution suggested by Guinness and Fuentes (2017) is to use a circulant approximation of the covariance on a smaller rectangular lattice. In that case, the method is no longer exact, but Guinness and Fuentes (2017) showed via simulations that using small embeddings can in some cases provide a good compromise between statistical and computational efficiency.

In contrast, in this paper we revisited the root cause of why the approximation of the likelihood may deteriorate, while continuing to require that any proposed bias elimination result in a computationally competitive method. Our method of bias elimination is ‘built in’ by fitting the periodogram to its expectation ${\bar{I}}_{n}\left(\omega ;\theta \right)$. This is in contrast to estimating the bias and removing it, which typically increases variance, and might lead to negative spectral density estimates.

We have thus proposed a bias elimination method that is data‐driven, fully automated and computationally practical for a number of realistic spatial sampling methods, in any dimension. Our methods are robust to huge volumes of missing data, as backed up by our theoretical analysis, and evidenced by our practical simulation examples. As a result, our methodology is not only of great benefit for improved parameter estimation directly, but also has knock‐on benefits in, for example, the problem of prediction. Here a huge number of methods exist and there is some debate as to which are most practically useful (Heaton et al., 2019). The broader point is that many of these methods are based on Matérn covariance kernels, and therefore our methods, which we have shown greatly improve Matérn parameter estimation, can be naturally incorporated to improve the performance of such spatial methods for prediction. Quantifying this benefit over a range of settings is a natural line of further investigation.

Within parameter estimation, there are a number of large outstanding challenges which are nontrivial extensions and merit further investigation as stand‐alone pieces of work: (a) extensions to fully irregularly sampled process on non‐uniform grids; and (b) extensions to multivariate processes with complex sampling patterns. In each case the impact on the Fourier transform and the expected periodogram need to be carefully handled to properly account for the bias of naively using basic Whittle‐type approximations. We do, however, expect that large improvements are possible both in terms of bias reduction (vs. standard Whittle methods where edge effect contamination will increase), and in terms of computational speed (vs. exact likelihood and other pseudo‐likelihoods which will become increasingly intractable as assumptions are relaxed).
