# Supplementary material for: The Debiased Spatial Whittle likelihood
Source: J R Stat Soc Series B Stat Methodol. 2022 Jul 20;84(4):1526–57. doi: 10.1111/rssb.12539 (PMC9796718; doi:10.1111/rssb.12539)
Supplement: Supplementary file 1 [file RSSB-84-1526-s001.pdf]

The Debiased Spatial Whittle Likelihood – Supplementary material

Arthur P. Guillaumin

Queen Mary University of London, London, UK

Adam M. Sykulski

Lancaster University, United Kingdom.

Sofia C. Olhede

École polytechnique fédérale de Lausanne, Switzerland.
University College London, UK

Frederik J. Simons

Princeton University, USA.

Aliased Whittle likelihood comparison

In this section, we provide simulation results in the same manner as those of Section 6.1 in the main document, except for the fact that here both the Whittle and tapered Whittle estimator use a truncated approximation of the aliased spectral density of the sampled process, see Figure 1. We limited the approximation to include the contribution of frequencies from $[-3\pi, 3\pi]^2$ to keep computational cost reasonable. The fact that we use a fixed approximation to the aliased spectral density explains why, despite largely reducing the bias for the Whittle and tapered Whittle, in comparison to the version in the main document, the efficiency of both the Whittle and tapered Whittle estimators appears to saturate for large grid sizes.

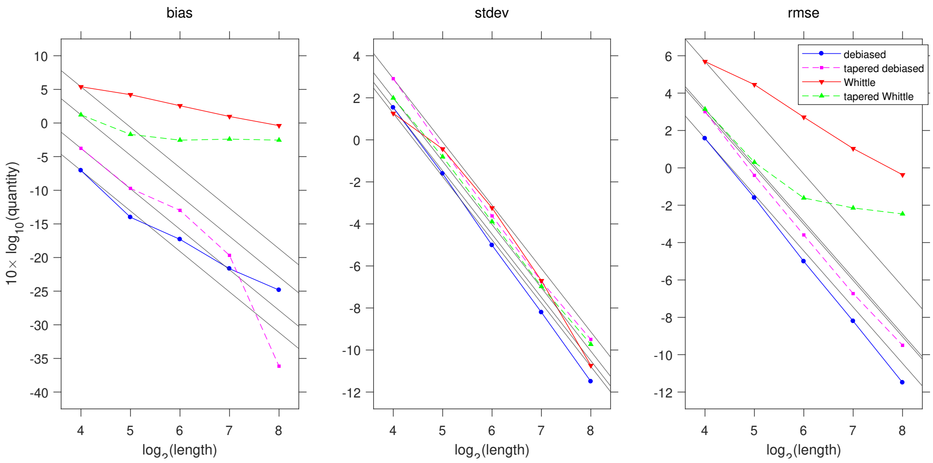

Fig. 1. Bias, standard deviation, and root mean-squared error of estimates of the range parameter $\rho = 10$ of a Matérn process (39) with $\nu = 1/2, \sigma^2 = 1$. Compared to Figure 1 in the main document, the Whittle and tapered Whittle estimation methods use an approximation to the aliased spectral density function, by incorporating contributions from frequencies within the square domain $[-3\pi, 3\pi]^2$.

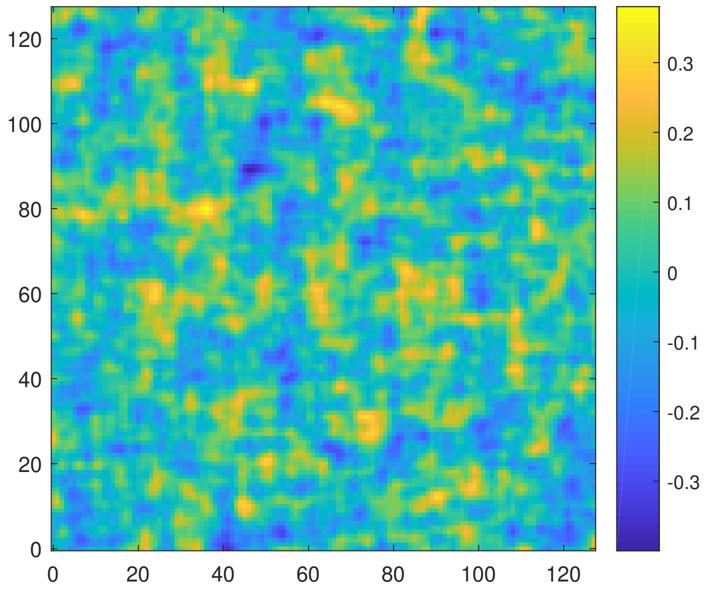

Fig. 2. Simulated sample from the discrete model defined by equation (1), with $\theta = 3$.

Estimation for a discrete spatial model

In this section we apply the Spatial Debiased Whittle to the estimation of a discrete parametric model. In comparison to continuous models, the estimation of the parameters of a discrete spatial model is not hindered by aliasing. The model we consider is defined in the frequency domain according to,

$$f(\omega_1, \omega_2) = \begin{cases} \exp\{-\theta(|\omega_1| + |\omega_2|)\} & \text{if} \quad \boldsymbol{\omega} \in (-\pi, \pi)^2 \\ 0 & \text{o/w} \end{cases}, \tag{1}$$

where $\theta \geq 0$. The covariance function of this model is easily obtained analytically, and takes the form of,

$$c_X(u_1, u_2) = 4\mathcal{R}\left\{\frac{1}{iu_1 - \theta}\left(\exp\left[(iu_1 - \theta)\pi\right] - 1\right)\right\}\mathcal{R}\left\{\frac{1}{iu_2 - \theta}\left(\exp\left[(iu_2 - \theta)\pi\right] - 1\right)\right\}, \tag{2}$$

which is separable in u_1 and u_2, since the spectrum is separable in ω_1 and ω_2. We display a simulated realization in Figure 2.

In our experiments we set $\theta = 3$ and initialize estimates to 0.2 for all estimation methods. In a first experiment we consider estimation on growing squares, see Figure 3. The tapered Whittle method performs very well for this discrete model for large sizes, but suffers from bias for smaller grid sizes. The tapered version of the Spatial Debiased Whittle performs better than its non-tapered counter-part, due to remaining boundary effects. However, it is notable that even without tapering, the Spatial Debiased Whittle appears to perform at the expected square root n rate.

In a second experiment, we demonstrate the ability of the Spatial Debiased Whittle to perform well for rectangular but not square domains, see Figure 4. We fix one side of the domain to 16

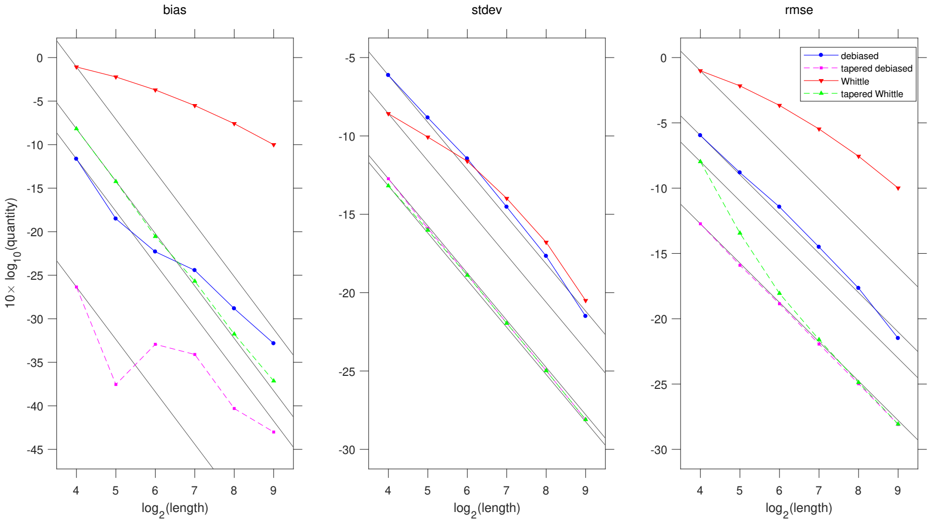

Fig. 3. Bias, standard deviation, and RMSE of estimates of $\theta = 3$ for rectangular grids of size $N \times N$ where N increases in powers of 2 which are indicated by the values on the x-axis. All estimators are initialized to the value 0.2.

units, while the other side grows in powers of 4, so that the sample sizes increase in the same way as in the previous experiment. In this configuration, the asymptotic bias of the tapered Whittle is non-zero—this is because the expected periodogram never converges to the spectral density, due to the bounded sample size along one dimension. In contrast, the observed rate of the Spatial Debiased Whittle likelihood remains of the order of square root the sample size.

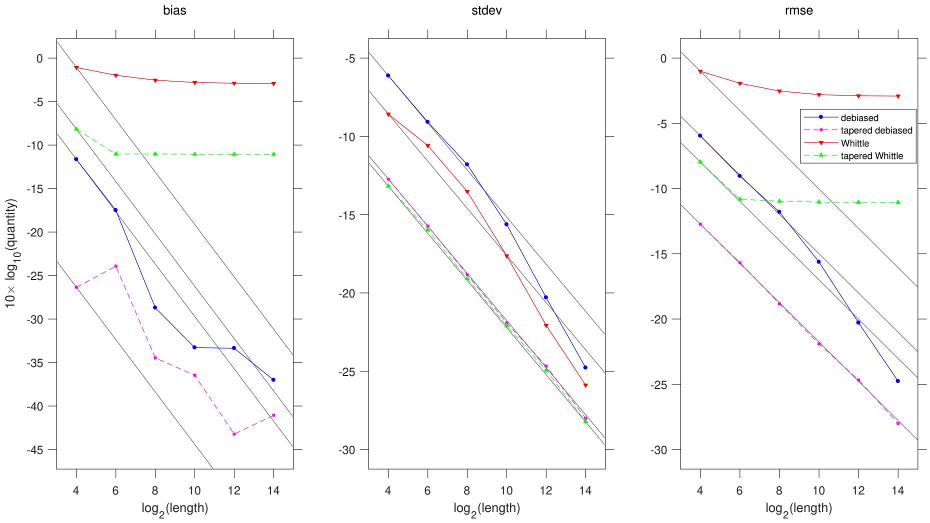

Fig. 4. Bias, standard deviation, and RMSE of estimates of $\theta = 3$ for rectangular grids of size $16 \times N$ where N increases in powers of 4 and is indicated on the x-axis. We observe how even for a simple discrete model, tapering has its limits and cannot fully account for the shape of the observational domain.

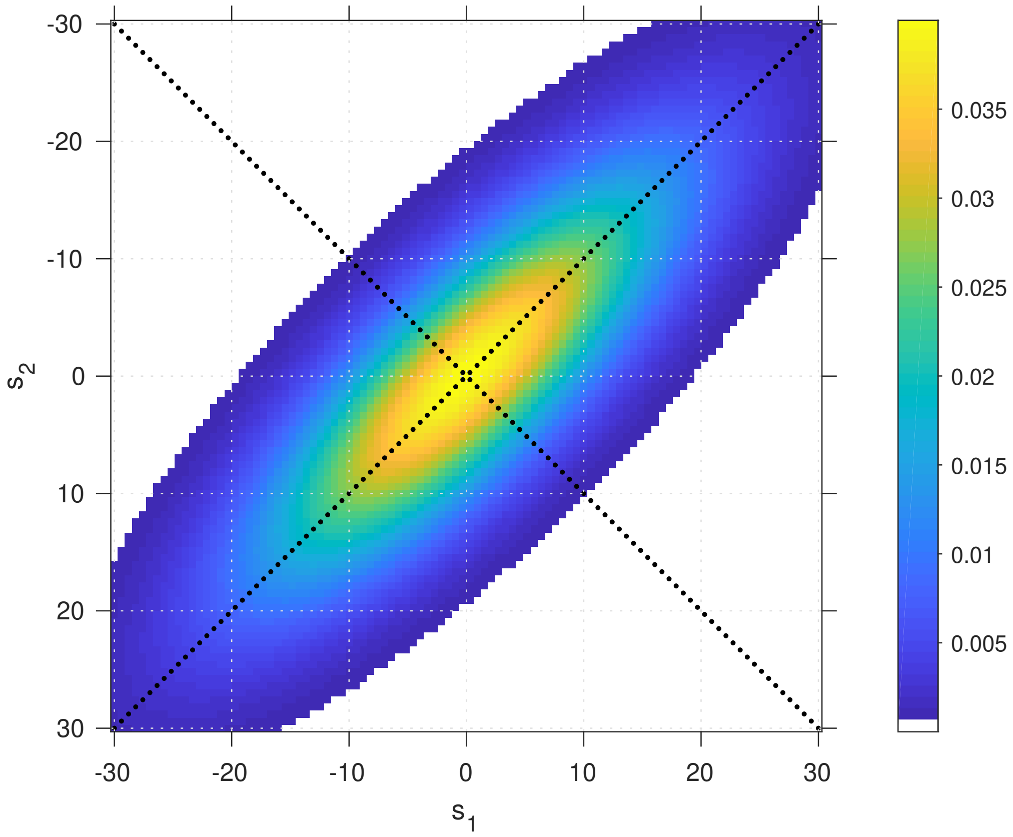

Fig. 5. This figure illustrates the geometric characteristics of Significant Correlation Contribution in 2D. We plot $c_X(\mathbf{u};\boldsymbol{\theta})$ as a contour plot and superimpose $\mathbf{s}_{1,j}$ and $\mathbf{s}_{2,j}$, even if $\mathbf{s}_{1,j}$ and $\mathbf{s}_{2,j}$ are used to sample $X^{(r)}(\mathbf{s})$ to give $X^{(r)}_{\mathbf{s}}$ rather than sampling the covariance kernel.

Example of a violation of SCC

We start by assuming that the autocovariance is

$$c_X(\mathbf{u};\boldsymbol{\theta}) = 0.04 \times \exp\left\{-\frac{\theta_1}{2}(u_1+u_2)^2\right\} \exp\left\{-\frac{\theta_2}{2}(-u_1+u_2)^2\right\}, \tag{3}$$

and then we sample the process according to

$$\mathbf{s}_{1,j} = \begin{pmatrix} j & j \end{pmatrix}, \quad \mathbf{s}_{2,j} = \begin{pmatrix} -j & j \end{pmatrix}.$$

It is fairly straightforward, with either these line samplings, to convince oneself that with one sampling we only learn about θ_1 and with the other only about θ_2 as illustrated by Figure 5. Note that $c_X()$ is a valid auto–covariance, as the Fourier transform of Gaussians is Gaussian and thus non–negative. Sampling the process $\mathbf{X}(\mathbf{s})$ with $\mathbf{s}_{1,j}$ means that sums and differences of the sampling pattern lives in the same linear subspace of $\mathbb{R}^2$. This means that we only learn about one of the two functions in (3).

Proofs of lemmata, propositions and theorems

Proof of lemma 2

Proof. Let $\mathbf{k} = (k_0, \ldots, k_{d-1}) \in \prod_{j=0}^{d-1} \{0, \ldots, n_j - 1\}$. We remind the reader that for $\mathbf{u} \in \mathbb{Z}^d$, $\bar{c}_{\mathbf{n}}(\mathbf{u}) = c_{g,\mathbf{n}}(\mathbf{u}) c_X(\mathbf{u})$, where,

$$c_{g,\mathbf{n}}(\mathbf{u}) = \frac{\sum_{\mathbf{s} \in \mathbb{Z}^d} g_{\mathbf{s}} g_{\mathbf{s}+\mathbf{u}}}{\sum_{\mathbf{s} \in \mathbb{Z}^d} g_{\mathbf{s}}^2}.$$

Using the fact that for any $\mathbf{q} \in \{0, 1\}^d$,

$$\bar{c}_{\mathbf{n}}(\mathbf{u} - \mathbf{q} \circ \mathbf{n}) \exp\left(-i \sum_{j=0}^{d-1} \frac{2k_j \pi}{n_j}(u_j - q_j n_j)\right) = \bar{c}_{\mathbf{n}}(\mathbf{u} - \mathbf{q} \circ \mathbf{n}) \exp\left(-i \sum_{j=0}^{d-1} \frac{2k_j \pi}{n_j} u_j\right),$$

where $\circ$ denotes the Hadamard product, i.e. element-wise multiplication, and since $\bar{c}_{\mathbf{n}}(\mathbf{u} - \mathbf{q} \circ \mathbf{n})$ is zero if any component of $\mathbf{u}$ is zero and the corresponding component of $\mathbf{q}$ is one (due to the definition of $c_{g,\mathbf{n}_k}$), we obtain the proposed formula. Indeed, any $\mathbf{u} \in \prod_{j=0}^{d-1} \{-(n_j - 1), \ldots, n_j - 1\}$ that contribute to the LHS of the proposed formula can be written as $\mathbf{u} = \mathbf{u}^+ - \mathbf{q} \circ \mathbf{n}$ for some unique $\mathbf{u}^+ \in \prod_{j=0}^{d-1} \{0, \ldots, n_j - 1\}$. The extra terms in the RHS of the proposed formula take value zero according to the previous argument.

Proof of Lemma 6

Proof. This comes as a consequence of the two following observations. First, two continuous functions on $\mathcal{T}^d$ are equal if and only if their Fourier coefficients are equal, see for instance Körner (1988). Second, for a sequence of full rectangular grids indexed by $k \in \mathbb{N}$ that grow unbounded in all directions, for any $\mathbf{u} \in \mathbb{Z}^d$, we have $c_{g,n_k}(\mathbf{u}) \to 1$ as k goes to infinity, see equation (16) in the main body.

Proof of Lemma 7

Proof. The argument is very similar to that of Lemma 6, with the difference that for any $\mathbf{u} \in \mathbb{Z}^d$ we have that $c_{g,n_k}(\mathbf{u})$ converges to a positive constant (which might be strictly smaller than one) as k goes to infinity.

Proof of Theorem 1

Proof. We will show in Lemma 15 that $l_{\mathbf{n}_k}(\cdot)$, as a random function, converges uniformly to $\widetilde{l}_{\mathbf{n}_k}(\cdot)$ in probability, i.e. their difference converges uniformly to the zero function in probability. Hence the difference $l_{\mathbf{n}_k}(\hat{\boldsymbol{\theta}}_k) - \widetilde{l}_{\mathbf{n}_k}(\hat{\boldsymbol{\theta}}_k)$ converges to zero in probability. Additionally, $l_{\mathbf{n}_k}(\hat{\boldsymbol{\theta}}_k) - \widetilde{l}_{\mathbf{n}_k}(\boldsymbol{\theta})$ converges to zero in probability. Indeed, by definition, the parameter vector $\hat{\boldsymbol{\theta}}_k$ minimizes the function $l_{\mathbf{n}_k}(\cdot)$ over the parameter set Θ, and according to Lemma 9, the parameter vector $\boldsymbol{\theta}$ minimizes the function $\widetilde{l}_{\mathbf{n}_k}(\cdot)$. We therefore have, by the triangle inequality,

$$\left|\widetilde{l}_{\mathbf{n}_k}(\boldsymbol{\theta}) - \widetilde{l}_{\mathbf{n}_k}(\hat{\boldsymbol{\theta}}_k)\right| \leq \left|l_{\mathbf{n}_k}(\hat{\boldsymbol{\theta}}_k) - \widetilde{l}_{\mathbf{n}_k}(\hat{\boldsymbol{\theta}}_k)\right| + \left|l_{\mathbf{n}_k}(\hat{\boldsymbol{\theta}}_k) - \widetilde{l}_{\mathbf{n}_k}(\boldsymbol{\theta})\right|,$$

which converges to zero in probability. Making use of Lemma 14 we conclude that $\hat{\boldsymbol{\theta}}_k$ converges in probability to $\boldsymbol{\theta}$.

Proof of Proposition 1

Proof. We write the proof for the case $\Omega_{\mathbf{n}} = \Omega_{\mathbf{n}}^{(1)}$, while the case $\Omega_{\mathbf{n}} = \Omega_{\mathbf{n}}^{(2)}$ is the same, up to a constant factor. We first write the proof of the proposition for the univariate Gaussian case. Let $a_{\max} > 0$ be a finite constant such that $|a_{\mathbf{n}}(\boldsymbol{\omega})| \leq a_{\max}, \forall \boldsymbol{\omega} \in \mathcal{T}, \forall \mathbf{n} \in \mathbb{N}^d$. We first make the observation that the sum of the periodogram values at the Fourier frequencies is the squared L_2 norm of the sample, up to some multiplicative constant, since the Discrete Fourier Transform is orthonormal, i.e.

$$\sum_{\boldsymbol{\omega} \in \Omega_{\mathbf{n}}} I_{\mathbf{n}}(\boldsymbol{\omega}) = \frac{|\mathbf{n}|}{(2\pi)^d \sum_{\mathbf{s} \in \mathbb{Z}^d} g_{\mathbf{s}}^2} \sum_{\mathbf{s} \in \mathbb{Z}^d} g_{\mathbf{s}}^2 X_{\mathbf{s}}^2.$$

Therefore,

$$\begin{aligned} \operatorname{var}\left\{ |\mathbf{n}|^{-1} \sum_{\boldsymbol{\omega} \in \Omega_{\mathbf{n}}} a_{\mathbf{n}}(\boldsymbol{\omega}) I_{\mathbf{n}}(\boldsymbol{\omega}) \right\} &\leq a_{\max}^2 \operatorname{var}\left\{ |\mathbf{n}|^{-1} \sum_{\boldsymbol{\omega} \in \Omega_{\mathbf{n}}} I_{\mathbf{n}}(\boldsymbol{\omega}) \right\} \\ &= \frac{a_{\max}^2}{(2\pi)^{2d} \left(\sum g_{\mathbf{s}}^2\right)^2} \operatorname{var}\left\{ \sum_{\mathbf{s} \in \mathbb{Z}^d} g_{\mathbf{s}}^2 X_{\mathbf{s}}^2 \right\}. \end{aligned} \tag{4}$$

Note that the first inequality is valid since the covariance of the periodogram at two Fourier frequencies $\boldsymbol{\omega}, \boldsymbol{\omega}'$ is non-negative for a Gaussian process (as a consequence of Isserlis' theorem). Indeed, letting

$$J(\boldsymbol{\omega}) = \frac{(2\pi)^{-d/2}}{\sqrt{\sum_{\mathbf{s} \in \mathcal{J}_{\mathbf{n}}} g_{\mathbf{s}}^2}} \sum_{\mathbf{s} \in \mathcal{J}_{\mathbf{n}}} g_{\mathbf{s}} X_{\mathbf{s}} \exp(-i\boldsymbol{\omega} \cdot \mathbf{s}),$$

we have, by Isserlis' theorem,

$$\begin{aligned} \operatorname{cov}\left\{ I(\boldsymbol{\omega}), I(\boldsymbol{\omega}') \right\} &= \mathrm{E}\left\{ J(\boldsymbol{\omega}) J^*(\boldsymbol{\omega}) J(\boldsymbol{\omega}') J^*(\boldsymbol{\omega}') \right\} - \mathrm{E}\left\{ I(\boldsymbol{\omega}) \right\} \mathrm{E}\left\{ I(\boldsymbol{\omega}') \right\} \\ &= \mathrm{E}\left\{ J(\boldsymbol{\omega}) J(\boldsymbol{\omega}') \right\} \mathrm{E}\left\{ J^*(\boldsymbol{\omega}) J^*(\boldsymbol{\omega}') \right\} + \mathrm{E}\left\{ J(\boldsymbol{\omega}) J^*(\boldsymbol{\omega}') \right\} \mathrm{E}\left\{ J^*(\boldsymbol{\omega}) J(\boldsymbol{\omega}') \right\} \\ &= \left| \mathrm{E}\left\{ J(\boldsymbol{\omega}) J(\boldsymbol{\omega}') \right\} \right|^2 + \left| \mathrm{E}\left\{ J^*(\boldsymbol{\omega}) J(\boldsymbol{\omega}') \right\} \right|^2, \end{aligned}$$

which is non-negative as the sum of two squares. We study the term $\operatorname{var}\left\{ \sum_{\mathbf{s} \in \mathbb{Z}^d} g_{\mathbf{s}}^2 X_{\mathbf{s}}^2 \right\}$. We have, again using Isserlis' theorem for Gaussian random variables,

$$\begin{aligned} \operatorname{var}\left\{ \sum_{\mathbf{s} \in \mathbb{Z}^d} g_{\mathbf{s}}^2 X_{\mathbf{s}}^2 \right\} &= \mathrm{E}\left(\sum_{\mathbf{s} \in \mathcal{J}_{\mathbf{n}}} g_{\mathbf{s}}^2 X_{\mathbf{s}}^2 \right)^2 - \left(\mathrm{E} \sum_{\mathbf{s} \in \mathcal{J}_{\mathbf{n}}} g_{\mathbf{s}}^2 X_{\mathbf{s}}^2 \right)^2 \\ &= \sum_{\mathbf{s} \in \mathcal{J}_{\mathbf{n}}} \sum_{\mathbf{s}' \in \mathcal{J}_{\mathbf{n}}} \mathrm{E}\left\{ g_{\mathbf{s}}^2 g_{\mathbf{s}'}^2 X_{\mathbf{s}}^2 X_{\mathbf{s}'}^2 \right\} - \mathrm{E}\left\{ g_{\mathbf{s}}^2 X_{\mathbf{s}}^2 \right\} \mathrm{E}\left\{ g_{\mathbf{s}'}^2 X_{\mathbf{s}'}^2 \right\} \\ &= 2 \sum_{\mathbf{s} \in \mathcal{J}_{\mathbf{n}}} \sum_{\mathbf{s}' \in \mathcal{J}_{\mathbf{n}}} g_{\mathbf{s}}^2 g_{\mathbf{s}'}^2 \left(\mathrm{E}\left\{ X_{\mathbf{s}} X_{\mathbf{s}'} \right\} \right)^2. \end{aligned} \tag{5}$$

We now obtain, combining equations (4) and (5),

$$\begin{aligned}\operatorname{var}\left\{|\mathbf{n}_k|^{-1}\sum_{\boldsymbol{\omega}\in\Omega_{\mathbf{n}}} a_{\mathbf{n}}(\boldsymbol{\omega})I_{\mathbf{n}}(\boldsymbol{\omega})\right\} &\leq \frac{2a_{\max}^2}{(2\pi)^{2d}\left(\sum g_{\mathbf{s}}^2\right)^2}\sum_{\mathbf{s}\in\mathcal{J}_{\mathbf{n}}}\sum_{\mathbf{s}'\in\mathcal{J}_{\mathbf{n}}} g_{\mathbf{s}}^2 g_{\mathbf{s}'}^2\left(\mathrm{E}\left\{X_{\mathbf{s}}X_{\mathbf{s}'}\right\}\right)^2\\
&\leq \frac{2a_{\max}^2}{(2\pi)^{2d}\left(\sum g_{\mathbf{s}}^2\right)^2}\sum_{\mathbf{u}\in\mathbb{Z}^d} c_X(\mathbf{u})^2\sum_{\mathbf{s}\in\mathcal{J}_{\mathbf{n}}} g_{\mathbf{s}}^2 g_{\mathbf{s}+\mathbf{u}}^2\\
&\leq \frac{2a_{\max}^2}{(2\pi)^{2d}\left(\sum g_{\mathbf{s}}^2\right)^2}\sum_{\mathbf{u}\in\mathbb{Z}^d} c_X(\mathbf{u})^2\sum_{\mathbf{s}\in\mathcal{J}_{\mathbf{n}}} g_{\mathbf{s}} g_{\mathbf{s}+\mathbf{u}}\\
&\leq \frac{2a_{\max}^2}{(2\pi)^{2d}\sum g_{\mathbf{s}}^2}\sum_{\mathbf{u}\in\mathbb{Z}^d} c_X(\mathbf{u})^2 c_g(\mathbf{u}),\end{aligned}$$

where we have made use of the assumption that $0 \leq g_{\mathbf{s}} \leq 1, \forall \mathbf{s} \in \mathbb{Z}^d$. Therefore, we obtain the stated result, i.e.,

$$\operatorname{var}\left\{|\mathbf{n}_k|^{-1}\sum_{\boldsymbol{\omega}\in\Omega_{\mathbf{n}_k}} a_k(\boldsymbol{\omega})I_{\mathbf{n}_k}(\boldsymbol{\omega})\right\} = \mathcal{O}\left\{\frac{\sum_{\mathbf{u}\in\mathbb{Z}^d} c_X(\mathbf{u})^2 c_{g,k}(\mathbf{u})}{\sum g_{\mathbf{s}}^2}\right\},$$

where the big O is with respect to k going to infinity. This concludes the proof for the univariate Gaussian case. □

Proof of Corollary 1

We now treat the extension to the univariate but non-Gaussian case. This requires defining the fourth-order cumulant according to,

$$\begin{aligned}\mathrm{E}\{X_{\mathbf{s}_1}X_{\mathbf{s}_2}X_{\mathbf{s}_3}X_{\mathbf{s}_4}\} &= c_4(\mathbf{s}_2-\mathbf{s}_1,\mathbf{s}_3-\mathbf{s}_1,\mathbf{s}_4-\mathbf{s}_1) + c_X(\mathbf{s}_3-\mathbf{s}_1)c_X(\mathbf{s}_4-\mathbf{s}_2)\\
&\quad + c_X(\mathbf{s}_4-\mathbf{s}_1)c_X(\mathbf{s}_3-\mathbf{s}_2) + c_X(\mathbf{s}_2-\mathbf{s}_1)c_X(\mathbf{s}_4-\mathbf{s}_3).\end{aligned}$$

Note that in the Gaussian case this equality holds with $C_4(\mathbf{s}_2-\mathbf{s}_1,\mathbf{s}_3-\mathbf{s}_1,\mathbf{s}_4-\mathbf{s}_1)=0$ (trivially) always. With this definition, we can study the covariance of the periodogram at two Fourier frequencies as follows,

$$\begin{aligned}\operatorname{cov}\{I_{\mathbf{n}}(\boldsymbol{\omega}_1), I_{\mathbf{n}}(\boldsymbol{\omega}_2)\} &= \frac{(2\pi)^{-2d}}{(\sum_{\mathbf{s}} g_{\mathbf{s}}^2)^2}\operatorname{cov}\left\{\sum_{\mathbf{s}_1,\mathbf{s}_2} g_{\mathbf{s}_1}g_{\mathbf{s}_2}X_{\mathbf{s}_1}X_{\mathbf{s}_2}e^{-i\boldsymbol{\omega}_1^T(\mathbf{s}_1-\mathbf{s}_2)}, \sum_{\mathbf{s}_3,\mathbf{s}_4} g_{\mathbf{s}_3}g_{\mathbf{s}_4}X_{\mathbf{s}_3}X_{\mathbf{s}_4}e^{-i\boldsymbol{\omega}_2^T(\mathbf{s}_3-\mathbf{s}_4)}\right\}\\
&= \frac{(2\pi)^{-2d}}{(\sum_{\mathbf{s}} g_{\mathbf{s}}^2)^2}\sum_{\mathbf{s}_1,\mathbf{s}_2,\mathbf{s}_3,\mathbf{s}_4} g_{\mathbf{s}_1}g_{\mathbf{s}_2}g_{\mathbf{s}_3}g_{\mathbf{s}_4}\operatorname{cov}\{X_{\mathbf{s}_1}X_{\mathbf{s}_2}, X_{\mathbf{s}_3}X_{\mathbf{s}_4}\}e^{-i\boldsymbol{\omega}_1^T(\mathbf{s}_1-\mathbf{s}_2)}e^{-i\boldsymbol{\omega}_2^T(\mathbf{s}_3-\mathbf{s}_4)}.\end{aligned}$$

We write $C_k = \frac{(2\pi)^{-d}}{\sum_{\mathbf{s}} g_{\mathbf{s}}^2}$, where the dependence on k comes from the implicit dependence of $\{g_{\mathbf{s}}\}$ on k. We note that we can determine directly that

$$\begin{aligned}\operatorname{cov}\{X_{\mathbf{s}_1}X_{\mathbf{s}_2}, X_{\mathbf{s}_3}X_{\mathbf{s}_4}\} &= \mathrm{E}\{X_{\mathbf{s}_1}X_{\mathbf{s}_2}X_{\mathbf{s}_3}X_{\mathbf{s}_4}\} - \mathrm{E}\{X_{\mathbf{s}_1}X_{\mathbf{s}_2}\}\mathrm{E}\{X_{\mathbf{s}_3}X_{\mathbf{s}_4}\}\\
&= c_4(\mathbf{s}_2-\mathbf{s}_1,\mathbf{s}_3-\mathbf{s}_1,\mathbf{s}_4-\mathbf{s}_1) + c_X(\mathbf{s}_3-\mathbf{s}_1)c_X(\mathbf{s}_4-\mathbf{s}_2)\\
&\quad + c_X(\mathbf{s}_4-\mathbf{s}_1)c_X(\mathbf{s}_3-\mathbf{s}_2). \qquad (6)\end{aligned}$$

We additionally define,

$$\mathcal{G}(\mathbf{s}_1,\mathbf{s}_2,\mathbf{s}_3) = c_X(\mathbf{s}_3-\mathbf{s}_1)c_X(\mathbf{s}_4-\mathbf{s}_2) + c_X(\mathbf{s}_4-\mathbf{s}_1)c_X(\mathbf{s}_3-\mathbf{s}_2),$$

where the choice of the letter $\mathcal{G}$ comes from the fact that in the Gaussian case $\operatorname{cov}\{X_{\mathbf{s}_1}X_{\mathbf{s}_2}, X_{\mathbf{s}_3}X_{\mathbf{s}_4}\}$ simplifies to this quantity. Now summing over 2-combinations of Fourier frequencies, we can apply the triangular inequality,

$$\left|\sum_{\boldsymbol{\omega_1},\boldsymbol{\omega_2}} a_{\boldsymbol{\omega_1}}a_{\boldsymbol{\omega_2}}\operatorname{cov}\{I_{\mathbf{n}}(\boldsymbol{\omega}_1), I_{\mathbf{n}}(\boldsymbol{\omega}_2)\}\right| =$$

$$\left|\sum_{\boldsymbol{\omega_1},\boldsymbol{\omega_2}} a_{\boldsymbol{\omega_1}}a_{\boldsymbol{\omega_2}}C_k^2 \sum_{\mathbf{s}_1,\mathbf{s}_2,\mathbf{s}_3,\mathbf{s}_4} g_{\mathbf{s}_1}g_{\mathbf{s}_2}g_{\mathbf{s}_3}g_{\mathbf{s}_4}\left\{c_4(\mathbf{s}_2-\mathbf{s}_1,\mathbf{s}_3-\mathbf{s}_1,\mathbf{s}_4-\mathbf{s}_1)+\mathcal{G}(\mathbf{s}_1,\mathbf{s}_2,\mathbf{s}_3)\right\}e^{-i\boldsymbol{\omega}_1^T(\mathbf{s}_1-\mathbf{s}_2)}e^{-i\boldsymbol{\omega}_2^T(\mathbf{s}_3-\mathbf{s}_4)}\right|$$

$$\leq \left|\sum_{\boldsymbol{\omega_1},\boldsymbol{\omega_2}} a_{\boldsymbol{\omega_1}}a_{\boldsymbol{\omega_2}}C_k^2 \sum_{\mathbf{s}_1,\mathbf{s}_2,\mathbf{s}_3,\mathbf{s}_4} g_{\mathbf{s}_1}g_{\mathbf{s}_2}g_{\mathbf{s}_3}g_{\mathbf{s}_4}c_4(\mathbf{s}_2-\mathbf{s}_1,\mathbf{s}_3-\mathbf{s}_1,\mathbf{s}_4-\mathbf{s}_1)e^{-i\boldsymbol{\omega}_1^T(\mathbf{s}_1-\mathbf{s}_2)}e^{-i\boldsymbol{\omega}_2^T(\mathbf{s}_3-\mathbf{s}_4)}\right|$$

$$+\left|\sum_{\boldsymbol{\omega_1},\boldsymbol{\omega_2}} a_{\boldsymbol{\omega_1}}a_{\boldsymbol{\omega_2}}C_k^2 \sum_{\mathbf{s}_1,\mathbf{s}_2,\mathbf{s}_3,\mathbf{s}_4} g_{\mathbf{s}_1}g_{\mathbf{s}_2}g_{\mathbf{s}_3}g_{\mathbf{s}_4}\mathcal{G}(\mathbf{s}_1,\mathbf{s}_2,\mathbf{s}_3)e^{-i\boldsymbol{\omega}_1^T(\mathbf{s}_1-\mathbf{s}_2)}e^{-i\boldsymbol{\omega}_2^T(\mathbf{s}_3-\mathbf{s}_4)}\right|$$

The second term in the sum has already been studied in the proof of Proposition 1 where we assumed Gaussianity. As for the first term, again using the triangular inequality, we may deduce that

$$\left|\sum_{\boldsymbol{\omega_1},\boldsymbol{\omega_2}} a_{\boldsymbol{\omega_1}}a_{\boldsymbol{\omega_2}}C_k^2 \sum_{\mathbf{s}_1}\sum_{\mathbf{s}_2}\sum_{\mathbf{s}_3}\sum_{\mathbf{s}_4} g_{\mathbf{s}_1}g_{\mathbf{s}_2}g_{\mathbf{s}_3}g_{\mathbf{s}_4}c_4(\mathbf{s}_2-\mathbf{s}_1,\mathbf{s}_3-\mathbf{s}_1,\mathbf{s}_4-\mathbf{s}_1)e^{-i\boldsymbol{\omega}_1^T(\mathbf{s}_1-\mathbf{s}_2)}e^{-i\boldsymbol{\omega}_2^T(\mathbf{s}_3-\mathbf{s}_4)}\right|$$
$$\leq \sum_{\boldsymbol{\omega_1},\boldsymbol{\omega_2}} a_{\boldsymbol{\omega_1}}a_{\boldsymbol{\omega_2}}C_k^2 \sum_{\mathbf{s}_1}\sum_{\mathbf{s}_2}\sum_{\mathbf{s}_3}\sum_{\mathbf{s}_4} g_{\mathbf{s}_1}g_{\mathbf{s}_2}g_{\mathbf{s}_3}g_{\mathbf{s}_4}\left|c_4(\mathbf{s}_2-\mathbf{s}_1,\mathbf{s}_3-\mathbf{s}_1,\mathbf{s}_4-\mathbf{s}_1)e^{-i\boldsymbol{\omega}_1^T(\mathbf{s}_1-\mathbf{s}_2)}e^{-i\boldsymbol{\omega}_2^T(\mathbf{s}_3-\mathbf{s}_4)}\right|$$
$$\leq \sum_{\boldsymbol{\omega_1},\boldsymbol{\omega_2}} a_{\boldsymbol{\omega_1}}a_{\boldsymbol{\omega_2}}\left|\mathbf{n}\right|C_k^2\sum_{\boldsymbol{\tau}_1}\sum_{\boldsymbol{\tau}_2}\sum_{\boldsymbol{\tau}_3}\left|c_4(\boldsymbol{\tau}_1,\boldsymbol{\tau}_2,\boldsymbol{\tau}_3)\right|.$$

We now make use of our assumption of absolute summability of fourth-order cumulants. Defining the positive finite constant $K_4 = \sum_{\boldsymbol{\tau}_1=0}^{\infty}\sum_{\boldsymbol{\tau}_2=0}^{\infty}\sum_{\boldsymbol{\tau}_3=0}^{\infty}\left|c_4(\boldsymbol{\tau}_1,\boldsymbol{\tau}_2,\boldsymbol{\tau}_3)\right| < \infty$, we obtain,

$$\frac{1}{|\mathbf{n}_k|^2}\left|\sum_{\boldsymbol{\omega_1},\boldsymbol{\omega_2}} a_{\boldsymbol{\omega_1}}a_{\boldsymbol{\omega_2}}\operatorname{cov}\{I_{\mathbf{n}}(\boldsymbol{\omega}_1), I_{\mathbf{n}}(\boldsymbol{\omega}_2)\}\right| \leq \frac{\sum_{\mathbf{u}\in\mathbb{Z}^d} c_X(\mathbf{u})^2 c_{g,k}(\mathbf{u})}{\sum g_{\mathbf{s}}^2} + |\mathbf{n}_k| C_k^2 K_4,$$

where the first term is the one obtained also for Gaussian random fields. This allows us to conclude, under our assumption of absolute summability of fourth-order cumulants, that in the non-Gaussian case,

$$\operatorname{var}\left\{|\mathbf{n}_k|^{-1}\sum_{\boldsymbol{\omega}\in\Omega_{\mathbf{n}_k}} a_k(\boldsymbol{\omega})I_{\mathbf{n}_k}(\boldsymbol{\omega})\right\} = \mathcal{O}\left\{\frac{\sum_{\mathbf{u}\in\mathbb{Z}^d} c_X(\mathbf{u})^2 c_{g,k}(\mathbf{u})}{\sum g_{\mathbf{s}}^2} + \frac{|\mathbf{n}_k|}{(\sum g_{\mathbf{s}}^2)^2}\right\}. \tag{7}$$

□

Proof of Corollary 2

Proof. For a multivariate random field we proceed much in the same way as the proof of Proposition 1. We study the variance of the quadratic form

$$|\mathbf{n}_k|^{-1}\sum_{\boldsymbol{\omega}\in\Omega_{\mathbf{n}_k}} \mathbf{J}_{\mathbf{n}_k}^*(\boldsymbol{\omega})A_k(\boldsymbol{\omega})\mathbf{J}_{\mathbf{n}_k}(\boldsymbol{\omega}). \tag{8}$$

For all $\boldsymbol{\omega} \in \Omega_{\mathbf{n}_k}$ we perform an orthonormal eigendecomposition of $A_k(\boldsymbol{\omega})$,

$$A_k(\boldsymbol{\omega}) = \sum_{j=1}^{p} \lambda_j(\boldsymbol{\omega}) \mathbf{e}_j(\boldsymbol{\omega}) \mathbf{e}_j(\boldsymbol{\omega})^H,$$

where we do not indicate the dependence on k to avoid complicating the notation. We then define the complex-valued scalars,

$$Z_j = \mathbf{J}^H(\boldsymbol{\omega}) \mathbf{e}_j(\boldsymbol{\omega}), \quad j = 1, \ldots, p,$$

and note that, due to the orthonormality of the basis $\mathbf{e}_1, \ldots, \mathbf{e}_p$,

$$\mathbf{J}_{\mathbf{n}_k}(\boldsymbol{\omega}) = \sum_{j=1}^{p} Z_j(\boldsymbol{\omega}) \mathbf{e}_j(\boldsymbol{\omega}).$$

We have,

$$\begin{aligned} \operatorname{var}\left\{ |\mathbf{n}_k|^{-1} \sum_{\boldsymbol{\omega} \in \Omega_{\mathbf{n}_k}} \mathbf{J}_{\mathbf{n}_k}^H(\boldsymbol{\omega}) A_k(\boldsymbol{\omega}) \mathbf{J}_{\mathbf{n}_k}(\boldsymbol{\omega}) \right\} &= \operatorname{var}\left\{ |\mathbf{n}_k|^{-1} \sum_{\boldsymbol{\omega}} \sum_{j=1}^{p} \lambda_j(\boldsymbol{\omega}) |Z_j(\boldsymbol{\omega})|^2 \right\} \\ &= |\mathbf{n}_k|^{-2} \sum_{\boldsymbol{\omega}_1, \boldsymbol{\omega}_2} \sum_{j_1, j_2} \lambda_{j_1}(\boldsymbol{\omega}_1) \lambda_{j_2}(\boldsymbol{\omega}_2) \operatorname{cov}\{|Z_{j_1}(\boldsymbol{\omega}_1)|^2, |Z_{j_2}(\boldsymbol{\omega}_2)|^2\}. \end{aligned}$$

Using Isserliss' theorem we deduce, for any $\boldsymbol{\omega}_1, \boldsymbol{\omega}_2 \in \Omega_{\mathbf{n}_k}^2$, $j_1, j_2 = 1, \ldots, p$,

$$\begin{aligned} \operatorname{cov}\{|Z_{j_1}(\boldsymbol{\omega}_1)|^2, |Z_{j_2}(\boldsymbol{\omega}_2)|^2\} = {} & \mathrm{E}\{Z_{j_1}(\boldsymbol{\omega}_1) Z_{j_2}(\boldsymbol{\omega}_2)\} \mathrm{E}\{Z_{j_1}^*(\boldsymbol{\omega}_1) Z_{j_2}^*(\boldsymbol{\omega}_2)\} \\ & + \mathrm{E}\{Z_{j_1}(\boldsymbol{\omega}_1) Z_{j_2}^*(\boldsymbol{\omega}_2)\} \mathrm{E}\{Z_{j_1}^*(\boldsymbol{\omega}_1) Z_{j_2}(\boldsymbol{\omega}_2)\} \geq 0. \end{aligned}$$

Therefore it follows that

$$\operatorname{var}\left\{ |\mathbf{n}_k|^{-1} \sum_{\boldsymbol{\omega} \in \Omega_{\mathbf{n}_k}} \mathbf{J}_{\mathbf{n}_k}^H(\boldsymbol{\omega}) A_k(\boldsymbol{\omega}) \mathbf{J}_{\mathbf{n}_k}(\boldsymbol{\omega}) \right\} \leq \lambda_{\max}^2 |\mathbf{n}_k|^{-2} \sum_{\boldsymbol{\omega}_1} \sum_{\boldsymbol{\omega}_2} \sum_{j_1} \sum_{j_2} \operatorname{cov}\{|Z_{j_1}(\boldsymbol{\omega}_1)|^2, |Z_{j_2}(\boldsymbol{\omega}_2)|^2\}.$$

Besides,

$$\begin{aligned} \operatorname{var}\left\{ |\mathbf{n}_k|^{-1} \sum_{\boldsymbol{\omega} \in \Omega_{\mathbf{n}_k}} \mathbf{J}_{\mathbf{n}_k}^H(\boldsymbol{\omega}) \mathbf{J}_{\mathbf{n}_k}(\boldsymbol{\omega}) \right\} &= \operatorname{var}\left\{ |\mathbf{n}_k|^{-1} \sum_{\boldsymbol{\omega} \in \Omega_{\mathbf{n}_k}} \left(\sum_{j=1}^{p} Z_j^* \mathbf{e}_j^H(\boldsymbol{\omega}) \right) \left(\sum_{j=1}^{p} Z_j \mathbf{e}_j(\boldsymbol{\omega}) \right) \right\} \\ &= \operatorname{var}\left\{ |\mathbf{n}_k|^{-1} \sum_{\boldsymbol{\omega} \in \Omega_{\mathbf{n}_k}} \left(\sum_{j_1, j_2 = 1}^{p} Z_{j_1}^* Z_{j_2} \mathbf{e}_{j_1}^H(\boldsymbol{\omega}) \mathbf{e}_{j_2}(\boldsymbol{\omega}) \right) \right\} \\ &= \operatorname{var}\left\{ |\mathbf{n}_k|^{-1} \sum_{\boldsymbol{\omega} \in \Omega_{\mathbf{n}_k}} \sum_{j=1}^{p} |Z_j|^2 \right\}, \end{aligned}$$

after we again use the orthonormality of the basis $\mathbf{e}_1, \ldots, \mathbf{e}_p$. Hence we deduce that,

$$\operatorname{var}\left\{ |\mathbf{n}_k|^{-1} \sum_{\boldsymbol{\omega} \in \Omega_{\mathbf{n}_k}} \mathbf{J}_{\mathbf{n}_k}^H(\boldsymbol{\omega}) A_k(\boldsymbol{\omega}) \mathbf{J}_{\mathbf{n}_k}(\boldsymbol{\omega}) \right\} \leq \lambda_{\max}^2 \operatorname{var}\left\{ |\mathbf{n}_k|^{-1} \sum_{\boldsymbol{\omega} \in \Omega_{\mathbf{n}_k}} \mathbf{J}_{\mathbf{n}_k}^H(\boldsymbol{\omega}) \mathbf{J}_{\mathbf{n}_k}(\boldsymbol{\omega}). \right\}$$

As in the univariate case, we use the isometry property of the discrete Fourier transform to write this in the form of,

$$\operatorname{var}\left\{|\mathbf{n}_k|^{-1}\sum_{\boldsymbol{\omega}\in\Omega_{\mathbf{n}_k}}\mathbf{J}_{\mathbf{n}_k}(\boldsymbol{\omega})^H A_k(\boldsymbol{\omega})\mathbf{J}_{\mathbf{n}_k}(\boldsymbol{\omega})\right\}\leq\lambda^2_{\max}\operatorname{var}\left\{\sum_{q=1,\dots,p}\left\{\frac{(2\pi)^{-d}}{\sum g^{(q)\,2}_{\mathbf{s}'}}\sum_{\mathbf{s}}g^{(q)\,2}_{\mathbf{s}}X^{(q)\,2}_{\mathbf{s}}\right\}\right\}.$$

By applying the Isserlis theorem, we obtain the following upper-bound,

$$\begin{aligned}&\operatorname{var}\left\{|\mathbf{n}_k|^{-1}\sum_{\boldsymbol{\omega}\in\Omega_{\mathbf{n}_k}}\mathbf{J}_{\mathbf{n}_k}(\boldsymbol{\omega})^H A_k(\boldsymbol{\omega})\mathbf{J}_{\mathbf{n}_k}(\boldsymbol{\omega})\right\}\\&\leq 2\lambda^2_{\max}\sum_{q,r}\frac{(2\pi)^{-2d}}{\sum g^{(q)\,2}_{\mathbf{s}'}\sum g^{(r)\,2}_{\mathbf{s}'}}\left\{\sum_{s,s'}g^{(q)\,2}_{\mathbf{s}}g^{(r)\,2}_{\mathbf{s}'}\left(\mathrm{E}\left[X^{(r)}_{\mathbf{s}}X^{(q)}_{\mathbf{s}'}\right]\right)^2\right\}.\end{aligned}$$

By a manipulation similar to the one we used earlier for the univariate case, we obtain,

$$\operatorname{var}\left\{|\mathbf{n}_k|^{-1}\sum_{\boldsymbol{\omega}\in\Omega_{\mathbf{n}_k}}\mathbf{J}_{\mathbf{n}_k}(\boldsymbol{\omega})^* A_k(\boldsymbol{\omega})\mathbf{J}_{\mathbf{n}_k}(\boldsymbol{\omega})\right\}=\mathcal{O}\left\{\sum_{q,r}\frac{\sum_{\mathbf{u}}c^{(qr)}_X(\mathbf{u})^2c^{(qr)}_g(\mathbf{u})}{\sqrt{\sum g^{(q)\,2}_{\mathbf{s}}\sum g^{(r)\,2}_{\mathbf{s}}}}\right\},$$

which determines the order of the variance of such quadratic forms. □

Proof of Lemma 9

Proof. The difference between the expected likelihood function at the true parameter vector and any parameter vector $\boldsymbol{\gamma}\in\Theta$ takes the form

$$\widetilde{l}_{\mathbf{n}}(\gamma)-\widetilde{l}_{\mathbf{n}}(\boldsymbol{\theta})=|\mathbf{n}|^{-1}\sum_{\boldsymbol{\omega}\in\Omega_{\mathbf{n}}}\phi\left(\frac{\overline{I}_{\mathbf{n}_k}(\boldsymbol{\omega};\boldsymbol{\theta})}{\overline{I}_{\mathbf{n}_k}(\boldsymbol{\omega};\gamma)}\right),$$

with $\phi: x\mapsto x-\log x-1$. This function is non-negative and attains it minimum uniquely at $x=1$.

The proof in the multivariate case requires a bit more care than the univariate case but follows the same pattern. Following Taniguchi (1979) and Guillaumin et al (2017) for 1-d and the multivariate version provided in Hosoya and Taniguchi (1982) we define the function

$$\widetilde{l}_{\mathbf{n}}(\gamma)=|\mathbf{n}|^{-1}\sum_{\boldsymbol{\omega}}\left\{\log\det\{\bar{\mathbf{I}}(\boldsymbol{\omega};\gamma)\}+\operatorname{trace}\left\{\bar{\mathbf{I}}^{-1}(\boldsymbol{\omega};\gamma)\bar{\mathbf{I}}(\boldsymbol{\omega};\boldsymbol{\theta}\right\}\right\}.$$

We now note that

$$\widetilde{l}_{\mathbf{n}}(\gamma)-\widetilde{l}_{\mathbf{n}}(\boldsymbol{\theta})=|\mathbf{n}|^{-1}\sum_{\boldsymbol{\omega}}\left\{\operatorname{trace}\left\{\bar{\mathbf{I}}^{-1}(\boldsymbol{\omega};\gamma)\bar{\mathbf{I}}(\boldsymbol{\omega};\boldsymbol{\theta})\right\}-\log\frac{\det\bar{\mathbf{I}}(\boldsymbol{\omega};\boldsymbol{\theta})}{\det\bar{\mathbf{I}}(\boldsymbol{\omega};\gamma)}-p\right\}.$$

We define $\mathbf{B}_{\boldsymbol{\omega}}(\boldsymbol{\theta},\gamma)=\bar{\mathbf{I}}(\boldsymbol{\omega};\boldsymbol{\theta})\bar{\mathbf{I}}^{-1}(\boldsymbol{\omega};\gamma)$, and assume this matrix has positive eigenvalues $\{\beta_i(\omega)\}_{i=1}^p$. We then obtain,

$$\widetilde{l}_{\mathbf{n}}(\gamma)-\widetilde{l}_{\mathbf{n}}(\boldsymbol{\theta})=|\mathbf{n}|^{-1}\sum_{\boldsymbol{\omega}}\sum_j\{\beta_j-\log\beta_j-1\}.$$

From here, like in the univariate case we make use of the fact that $\phi: x\mapsto x-\log x-1$ is non-negative and attains it minimum uniquely at $x=1$, which corresponds to $\mathbf{B}_{\boldsymbol{\omega}}(\boldsymbol{\theta},\gamma)$ being the identity matrix. □

Proof of Lemma 10

Proof. By combining equations (4) and (12) in the main body then the periodogram can be expressed as

$$I_{\mathbf{n}}(\boldsymbol{\omega}) = \frac{(2\pi)^{-d}}{\sum g_{\mathbf{s}}^2} \left| \sum_{\mathbf{s}\in\mathcal{J}_{\mathbf{n}}} g_{\mathbf{s}} X_{\mathbf{s}} \exp(-i\boldsymbol{\omega}\cdot\mathbf{s}) \right|^2, \qquad \boldsymbol{\omega} \in \mathcal{T}^d.$$

Making use of equation (9) of the main body, we therefore have,

$$\overline{I}_{\mathbf{n}}(\boldsymbol{\omega};\gamma) = \int_{\mathcal{T}^d} f_{\delta,X}(\boldsymbol{\omega}-\boldsymbol{\lambda};\gamma)\mathcal{F}_{\mathbf{n}}(\boldsymbol{\lambda})d\boldsymbol{\lambda}.$$

Also,

$$\begin{aligned}
\int_{\mathcal{T}^d} \mathcal{F}_{\mathbf{n}}(\boldsymbol{\omega})d\boldsymbol{\omega} &= \frac{(2\pi)^{-d}}{\sum g_{\mathbf{s}}^2} \int_{\mathcal{T}^d} \left| \sum_{\mathbf{s}\in\mathcal{J}_{\mathbf{n}}} g_{\mathbf{s}} \exp(i\boldsymbol{\omega}\cdot\mathbf{s} \right|^2 d\,\boldsymbol{\omega} \\
&= \frac{(2\pi)^{-d}}{\sum g_{\mathbf{s}}^2} \int_{\mathcal{T}^d} \sum_{\mathbf{s}\in\mathcal{J}_{\mathbf{n}}} \sum_{\mathbf{s}'\in\mathcal{J}_{\mathbf{n}}} g_{\mathbf{s}} g_{\mathbf{s}'} \exp\{i\boldsymbol{\omega}\cdot(\mathbf{s}'-\mathbf{s})\}\, d\boldsymbol{\omega} \\
&= \frac{(2\pi)^{-d}}{\sum g_{\mathbf{s}}^2} \sum_{\mathbf{s}\in\mathcal{J}_{\mathbf{n}}} \sum_{\mathbf{s}'\in\mathcal{J}_{\mathbf{n}}} \int_{\mathcal{T}^d} g_{\mathbf{s}} g_{\mathbf{s}'} \exp\{i\boldsymbol{\omega}\cdot(\mathbf{s}'-\mathbf{s})\}\, d\boldsymbol{\omega} \\
&= \frac{1}{\sum g_{\mathbf{s}}^2} \sum_{\mathbf{s}\in\mathcal{J}_{\mathbf{n}}} \sum_{\mathbf{s}'\in\mathcal{J}_{\mathbf{n}}} g_{\mathbf{s}} g_{\mathbf{s}'} \delta_{\mathbf{s},\mathbf{s}'} \\
&= 1,
\end{aligned}$$

which is a direct adaptation of a standard result for the Féjer kernel. Hence,

$$\begin{aligned}
\left|\overline{I}_{\mathbf{n}}(\boldsymbol{\omega};\gamma)\right| &\leq \int_{\mathcal{T}^d} |f_{\delta,X}(\boldsymbol{\omega}-\boldsymbol{\lambda};\gamma)\mathcal{F}_{\mathbf{n}}(\boldsymbol{\lambda})|\, d\boldsymbol{\lambda} \\
&\leq f_{\delta,\max} \int_{\mathcal{T}^d} |\mathcal{F}_{\mathbf{n}}(\boldsymbol{\lambda})|\, d\boldsymbol{\lambda} \\
&\leq f_{\delta,\max}.
\end{aligned}$$

Similarly, we obtain the other inequality, i.e. $\overline{I}_{\mathbf{n}}(\boldsymbol{\omega};\gamma) \geq f_{\delta,\min}$, which concludes the proof. □

Proof of Lemma 11

We shall need the following intermediary result in our proof.

Lemma A. We have, for a growing domain,

$$|\mathbf{n}_k|^{-1} \sum_{\boldsymbol{\omega}\in\Omega_{\mathbf{n}_k}} \left\{\overline{I}_{\mathbf{n}_k}(\boldsymbol{\omega};\boldsymbol{\theta}) - \overline{I}_{\mathbf{n}_k}(\boldsymbol{\omega};\gamma)\right\}^2 = \sum_{\mathbf{u}\in\mathbb{Z}^d} \left\{\bar{c}_{\mathbf{n}_k}(\mathbf{u};\boldsymbol{\theta}) - \bar{c}_{\mathbf{n}_k}(\mathbf{u};\gamma)\right\}^2 + o(1).$$

Proof. We distinguish two cases:

(a) In the case where the domain is unbounded, we have set $\Omega_{\mathbf{n}} = \Omega_{\mathbf{n}}^{(1)}$, see the discussion following (5) in the main document. Then the result in obtained by application of Parseval's equality, according to which,

$$\sum_{\mathbf{u}\in\mathbb{Z}^d} \left\{\bar{c}_{\mathbf{n}_k}(\mathbf{u};\boldsymbol{\theta}) - \bar{c}_{\mathbf{n}_k}(\mathbf{u};\gamma)\right\}^2 = \int_{\mathcal{T}^d} \left\{\overline{I}_{\mathbf{n}_k}(\boldsymbol{\omega};\boldsymbol{\theta}) - \overline{I}_{\mathbf{n}_k}(\boldsymbol{\omega};\gamma)\right\}^2 d\boldsymbol{\omega},$$

and application of the Dominated Convergence Theorem.

(b) In the case where one or more dimensions of the domain are bounded, we use $\Omega_{\mathbf{n}} = \Omega_{\mathbf{n}}^{(2)}$, see the discussion following (5) in the main document, and in that case we have exactly,

$$|\mathbf{n}_k|^{-1} \sum_{\boldsymbol{\omega}\in\Omega_{\mathbf{n}_k}} \left\{\overline{I}_{\mathbf{n}_k}(\boldsymbol{\omega};\boldsymbol{\theta}) - \overline{I}_{\mathbf{n}_k}(\boldsymbol{\omega};\gamma)\right\}^2 = \sum_{\mathbf{u}\in\mathbb{Z}^d} \left\{\overline{c}_{\mathbf{n}_k}(\mathbf{u};\boldsymbol{\theta}) - \overline{c}_{\mathbf{n}_k}(\mathbf{u};\gamma)\right\}^2.$$

This can be established by direct calculations using the expression of the expected periodogram as a Fourier series provided in Lemma 1.

We can now establish the proof for Lemma 11.

Proof. We start by providing a proof in the scalar case (the non–Gaussian but scalar case requires no adjustment). We first observe, given equation (28) of the main body, that

$$\widetilde{l}_{\mathbf{n}_k}(\gamma) - \widetilde{l}_{\mathbf{n}_k}(\boldsymbol{\theta}) = |\mathbf{n}_k|^{-1} \sum_{\boldsymbol{\omega}\in\Omega_{\mathbf{n}_k}} \left\{\frac{\overline{I}_{\mathbf{n}_k}(\boldsymbol{\omega};\boldsymbol{\theta})}{\overline{I}_{\mathbf{n}_k}(\boldsymbol{\omega};\gamma)} - \log\frac{\overline{I}_{\mathbf{n}_k}(\boldsymbol{\omega};\boldsymbol{\theta})}{\overline{I}_{\mathbf{n}_k}(\boldsymbol{\omega};\gamma)} - 1\right\}.$$

As before, denoting $\phi : x \mapsto x - \log x - 1,\ x > 0$, and $g_{\mathbf{n}}(\boldsymbol{\omega})$ the piece-wise continuous function that maps any frequency of $\mathcal{T}^d$ to the closest smaller Fourier frequency corresponding to the grid $\mathcal{J}_{\mathbf{n}}$, we have

$$\widetilde{l}_{\mathbf{n}_k}(\gamma) - \widetilde{l}_{\mathbf{n}_k}(\boldsymbol{\theta}) = (2\pi)^{-d} \int_{\mathcal{T}^d} \phi\left(\frac{\overline{I}_{\mathbf{n}_k}(g(\boldsymbol{\omega});\boldsymbol{\theta})}{\overline{I}_{\mathbf{n}_k}(g(\boldsymbol{\omega});\gamma)}\right) d\boldsymbol{\omega}.$$

A Taylor expansion of $\phi(\cdot)$ around 1 gives, with $\psi(x) = (x-1)^2$,

$$\phi(x) = \psi(x)(1+\epsilon(x)),$$

where $\epsilon(x) \to 0$ as $x \to 1$. Therefore for any $\delta > 0$ there exists $\mu > 0$ such that for all x such that $|x-1| \le \mu$, $|\epsilon(x)| < \delta$. Now let, for all $k \in \mathbb{N}$,

$$\Pi_k = \left\{\boldsymbol{\omega} \in \mathcal{T}^d : \left|\frac{\overline{I}_{\mathbf{n}_k}(g(\boldsymbol{\omega});\boldsymbol{\theta})}{\overline{I}_{\mathbf{n}_k}(g(\boldsymbol{\omega});\gamma)} - 1\right| \le \mu\right\}.$$

We distinguish two cases:

(a) If for some $\delta > 0$, the Lebesgue measure of Π_k does not converge to $(2\pi)^d$, equation (30) of the main body holds.

(b) Otherwise, if for any $\delta > 0$ the Lebesgue measure of Π_k does converge to $(2\pi)^d$, we then have

$$\left|\widetilde{l}_{\mathbf{n}_k}(\gamma) - \widetilde{l}_{\mathbf{n}_k}(\boldsymbol{\theta})\right| = \int_{\Pi_k\cup\Pi_k^C} \psi\left(\frac{\overline{I}_{\mathbf{n}_k}(g(\boldsymbol{\omega});\boldsymbol{\theta})}{\overline{I}_{\mathbf{n}_k}(g(\boldsymbol{\omega});\gamma)}\right)\left\{1+\epsilon\left(\frac{\overline{I}_{\mathbf{n}_k}(g(\boldsymbol{\omega});\boldsymbol{\theta})}{\overline{I}_{\mathbf{n}_k}(g(\boldsymbol{\omega});\gamma)}\right)\right\} d\boldsymbol{\omega},$$

where Π_k^C denotes the complementary of Π_k as a subset of $\mathcal{T}^d$ and where the function $\epsilon(\cdot)$ was defined in equation (10). Denoting $h(\boldsymbol{\omega};\boldsymbol{\theta},\gamma) = \frac{\overline{I}_{\mathbf{n}_k}(g(\boldsymbol{\omega});\boldsymbol{\theta})}{\overline{I}_{\mathbf{n}_k}(g(\boldsymbol{\omega});\gamma)}$ (note that this quantity also depends on k),

$$\begin{aligned}\widetilde{l}_{\mathbf{n}_k}(\gamma) - \widetilde{l}_{\mathbf{n}_k}(\boldsymbol{\theta}) &= \int_{\mathcal{T}^d} \psi\left(h(\boldsymbol{\omega};\boldsymbol{\theta},\gamma)\right) d\boldsymbol{\omega} \\ &+ \int_{\Pi_k} \psi\left(h(\boldsymbol{\omega};\boldsymbol{\theta},\gamma)\right)\epsilon\left(h(\boldsymbol{\omega};\boldsymbol{\theta},\gamma)\right) d\boldsymbol{\omega} \\ &+ \int_{\Pi_k^C} \psi(h(\boldsymbol{\omega};\boldsymbol{\theta},\gamma)\epsilon(h(\boldsymbol{\omega};\boldsymbol{\theta},\gamma)) d\boldsymbol{\omega}.\end{aligned}$$

We shall now show that the two last terms of the right-hand side of this equation are asymptotically vanishing, so that we can limit our study to the first term, which will turn out to take a simple form in relation to our definition of significant correlation contribution (SCC) in the main body. Given the definition of Π_k we have,

$$\left| \int_{\Pi_k} \psi\left(h(\boldsymbol{\omega};\boldsymbol{\theta},\boldsymbol{\gamma})\right) \epsilon\left(h(\boldsymbol{\omega};\boldsymbol{\theta},\boldsymbol{\gamma})\right) d\boldsymbol{\omega} \right| \leq \delta \int_{\Pi_k} \psi\left(h(\boldsymbol{\omega};\boldsymbol{\theta},\boldsymbol{\gamma})\right) d\boldsymbol{\omega} \leq \delta \int_{\mathcal{T}^d} \psi\left(h(\boldsymbol{\omega};\boldsymbol{\theta},\boldsymbol{\gamma})\right) d\boldsymbol{\omega},$$

where the two inequalities come from the fact that the function $\psi(\cdot)$ is non-negative. We also have

$$\left| \int_{\Pi_k^C} \psi(h(\boldsymbol{\omega};\boldsymbol{\theta},\boldsymbol{\gamma})\epsilon(h(\boldsymbol{\omega};\boldsymbol{\theta},\boldsymbol{\gamma})) d\boldsymbol{\omega} \right| = o(1),$$

since the integrand is upper-bounded given Assumption 1.(1b) and since the measure of the set Π_k^C goes to zero. Hence we obtain, by the triangle inequality,

$$\left| \widetilde{l}_{\mathbf{n}_k}(\boldsymbol{\gamma}) - \widetilde{l}_{\mathbf{n}_k}(\boldsymbol{\theta}) \right| \geq \left(\int_{\mathcal{T}^d} \psi\left(h(\boldsymbol{\omega};\boldsymbol{\theta},\boldsymbol{\gamma})\right) d\boldsymbol{\omega} \right) (1-\delta) + o(1).$$

We now study the term $(2\pi)^{-d} \int_{\mathcal{T}^d} \psi\left(h(\boldsymbol{\omega};\boldsymbol{\theta},\boldsymbol{\gamma})\right) d\boldsymbol{\omega} = |\mathbf{n}_k|^{-1} \sum_{\boldsymbol{\omega}\in\Omega_{\mathbf{n}_k}} \left\{ \frac{\overline{I}_{\mathbf{n}_k}(\boldsymbol{\omega};\boldsymbol{\theta})}{\overline{I}_{\mathbf{n}_k}(\boldsymbol{\omega};\boldsymbol{\gamma})} - 1 \right\}^2$. We observe that

$$\begin{aligned} |\mathbf{n}_k|^{-1} \sum_{\boldsymbol{\omega}\in\Omega_{\mathbf{n}_k}} \left\{ \overline{I}_{\mathbf{n}_k}(\boldsymbol{\omega};\boldsymbol{\theta}) - \overline{I}_{\mathbf{n}_k}(\boldsymbol{\omega};\boldsymbol{\gamma}) \right\}^2 &= |\mathbf{n}_k|^{-1} \sum_{\boldsymbol{\omega}\in\Omega_{\mathbf{n}_k}} \overline{I}_{\mathbf{n}_k}(\boldsymbol{\omega};\boldsymbol{\gamma})^2 \left\{ \frac{\overline{I}_{\mathbf{n}_k}(\boldsymbol{\omega};\boldsymbol{\theta})}{\overline{I}_{\mathbf{n}_k}(\boldsymbol{\omega};\boldsymbol{\gamma})} - 1 \right\}^2 \\ &\leq |\mathbf{n}_k|^{-1} f_{\max,\delta}^2 \sum_{\boldsymbol{\omega}\in\Omega_{\mathbf{n}_k}} \left\{ \frac{\overline{I}_{\mathbf{n}_k}(\boldsymbol{\omega};\boldsymbol{\theta})}{\overline{I}_{\mathbf{n}_k}(\boldsymbol{\omega};\boldsymbol{\gamma})} - 1 \right\}^2. \end{aligned}$$

Additionally, according to Lemma A,

$$\begin{aligned} |\mathbf{n}_k|^{-1} \sum_{\boldsymbol{\omega}\in\Omega_{\mathbf{n}_k}} \left\{ \overline{I}_{\mathbf{n}_k}(\boldsymbol{\omega};\boldsymbol{\theta}) - \overline{I}_{\mathbf{n}_k}(\boldsymbol{\omega};\boldsymbol{\gamma}) \right\}^2 &= \sum_{\mathbf{u}\in\mathbb{Z}^d} \left\{ \bar{c}_{\mathbf{n}_k}(\mathbf{u};\boldsymbol{\theta}) - \bar{c}_{\mathbf{n}_k}(\mathbf{u};\boldsymbol{\gamma}) \right\}^2 + o(1) \\ &= \sum_{\mathbf{u}\in\mathbb{Z}^d} c_{g,\mathbf{n}_k}(\mathbf{u})^2 \left\{ c_X(\mathbf{u};\boldsymbol{\theta}) - c_X(\mathbf{u};\boldsymbol{\gamma}) \right\}^2 + o(1) \\ &\geq \frac{1}{2} \underline{\lim}_{k\to\infty} S_k(\boldsymbol{\theta},\boldsymbol{\gamma}) + o(1), \end{aligned}$$

where the last inequality holds for k sufficiently large, given the SCC assumption, see Definition 1. Therefore we obtain for k sufficiently large,

$$\left| \widetilde{l}_{\mathbf{n}_k}(\boldsymbol{\gamma}) - \widetilde{l}_{\mathbf{n}_k}(\boldsymbol{\theta}) \right| \geq \frac{1}{2 f_{\max,\delta}^2} (1-\delta) \underline{\lim}_{k\to\infty} S_k(\boldsymbol{\theta},\boldsymbol{\gamma}) + o(1).$$

Choosing $\delta = 1/2$, we obtain the inequality stated in equation (30) of the main body. This concludes the proof in the univariate case, as we have shown the absolute difference of the expected log-likelihood is lower bounded by the assumption of SCC.

We now extend the proof of Lemma 11 to the multivariate case. In the multivariate case, we first observe that we may write the difference of the expected log–likelihood for different

parameter values as

$$\begin{aligned}\widetilde{\ell}_{\mathbf{n}}(\gamma) - \widetilde{\ell}_{\mathbf{n}}(\boldsymbol{\theta}) &= |\mathbf{n}|^{-1} \sum_{\boldsymbol{\omega}} \left\{ \log\det\{\bar{\mathbf{I}}(\boldsymbol{\omega};\gamma)\} + \operatorname{trace}\left\{ \bar{\mathbf{I}}^{-1}(\boldsymbol{\omega};\gamma)\bar{\mathbf{I}}(\boldsymbol{\omega};\boldsymbol{\theta}) \right\} \right\} \\ &\quad - |\mathbf{n}|^{-1} \sum_{\boldsymbol{\omega}} \left\{ \log\det\{\bar{\mathbf{I}}(\boldsymbol{\omega};\boldsymbol{\theta})\} + \operatorname{trace}\left\{ \bar{\mathbf{I}}^{-1}(\boldsymbol{\omega};\boldsymbol{\theta})\bar{\mathbf{I}}(\boldsymbol{\omega};\boldsymbol{\theta}) \right\} \right\} \\ &= |\mathbf{n}|^{-1} \sum_{\boldsymbol{\omega}} \left\{ -\log\det\{\bar{\mathbf{I}}^{-1}(\boldsymbol{\omega};\gamma)\bar{\mathbf{I}}(\boldsymbol{\omega};\boldsymbol{\theta}\} + \operatorname{trace}\left\{ \bar{\mathbf{I}}^{-1}(\boldsymbol{\omega};\gamma)\bar{\mathbf{I}}(\boldsymbol{\omega};\boldsymbol{\theta} \right\} - p \right\}. \quad (9)\end{aligned}$$

We define $\tilde{\mathbf{B}}_{\boldsymbol{\omega}}(\boldsymbol{\theta},\gamma) = \bar{\mathbf{I}}^{-1}(\boldsymbol{\omega};\gamma)\bar{\mathbf{I}}(\boldsymbol{\omega};\boldsymbol{\theta})$, and assume this matrix has positive eigenvalues $\{\tilde{\beta}_i(\omega)\}_{i=1}^p$. Rewriting this expression in terms of the eigenvalues we get

$$\begin{aligned}\widetilde{\ell}_{\mathbf{n}}(\gamma) - \widetilde{\ell}_{\mathbf{n}}(\boldsymbol{\theta}) &= |\mathbf{n}|^{-1} \sum_{\boldsymbol{\omega}} \left\{ -\sum_j \log\tilde{\beta}_j(\omega) + \sum_j \tilde{\beta}_j(\omega) - p \right\} \\ &= |\mathbf{n}|^{-1} \sum_{\boldsymbol{\omega}} \sum_j \phi\{\tilde{\beta}_j(\boldsymbol{\omega})\}. \quad (10)\end{aligned}$$

We define $g_{\mathbf{n}}(\boldsymbol{\omega})$ as the piece-wise continuous function that maps any frequency of $\mathcal{T}^d$ to the closest smaller Fourier frequency corresponding to the grid of $\mathcal{I}_n$, we have

$$\widetilde{\ell}_{\mathbf{n}}(\gamma) - \widetilde{\ell}_{\mathbf{n}}(\boldsymbol{\theta}) = (2\pi)^{-d} \int_{\mathcal{T}^d} \sum_{j=1}^p \phi\left(\tilde{\beta}_j(g_{\mathbf{n}}(\boldsymbol{\omega}))\right) d\boldsymbol{\omega}. \quad (11)$$

A Taylor expansion of $\phi(\cdot)$ around 1 gives with $\phi(x) = \psi(x)(1+\epsilon(x))$ with $\psi(x) = (x-1)^2$, where $\epsilon(x)$ is going to zero as $x \to 1$. Most of this proceeds exactly like for the univariate case, but we shall now proceed to study what SCC means in this context. Unlike the univariate case we now have to propose a new approximation that works also in this case. Given we have

$$\begin{aligned}\widetilde{\ell}_{\mathbf{n}}(\gamma) - \widetilde{\ell}_{\mathbf{n}}(\boldsymbol{\theta}) &= \sum_{\boldsymbol{\omega}} \sum_j \left(\tilde{\beta}_j(g_{\mathbf{n}}(\boldsymbol{\omega})) - 1\right)^2 \\ &= \sum_{\boldsymbol{\omega}} \operatorname{trace}\left[\tilde{\mathbf{B}}_{\boldsymbol{\omega}}(\boldsymbol{\theta},\gamma) - I_p\right]^2 \\ &= \sum_{\boldsymbol{\omega}} \operatorname{trace}\left[\bar{\mathbf{I}}^{-1}(\boldsymbol{\omega};\gamma)\left(\bar{\mathbf{I}}(\boldsymbol{\omega};\boldsymbol{\theta}) - \bar{\mathbf{I}}(\boldsymbol{\omega};\gamma)\right)\right]^2 \\ &\geq f_{\max,\delta}^{-2} \sum_{\boldsymbol{\omega}} \operatorname{trace}\left[\bar{\mathbf{I}}(\boldsymbol{\omega};\boldsymbol{\theta}) - \bar{\mathbf{I}}(\boldsymbol{\omega};\gamma)\right]^2 \quad (12) \\ &= \sum_{\boldsymbol{\omega}} \sum_{q=1}^p \sum_{r=1}^p \left|\bar{I}^{(qr)}(\boldsymbol{\omega};\boldsymbol{\theta}) - \bar{I}^{(qr)}(\boldsymbol{\omega};\gamma)\right|^2, \quad (13)\end{aligned}$$

where the inequality results from Lemma 12. We can now relate the above quantity to the multivariate version of SCC via the use of Parseval's identity, just like we did in the univariate case. □

Proof of Lemma 12

Proof. Since H_1 is Hermitian positive definite it admits p real positive eigenvalues $0 < \lambda_1 \leq \ldots \leq \lambda_p$ and there exits a unitary matrix U such that $H_1 = U^*DU$, where D is the diagonal

matrix with elements $\lambda_1, \dots, \lambda_p$ on the diagonal. We then have,

$$\text{trace}\,[H_1H_2]^2 = \text{trace}\,[U^*DUH_2U^*DUH_2] = \text{trace}\,[DUH_2U^*DUH_2U^*] = \text{trace}\,[DZDZ]\,,$$

where $Z = UH_2U^*$, which is Hermitian positive definite just like H_2 is. Therefore,

$$\begin{aligned}\text{trace}\,[H_1H_2]^2 &= \sum_{j=1}^{p}\sum_{k=1}^{p}\lambda_j Z_{j,k}\lambda_k Z_{k,j} = \sum_{j=1}^{p}\sum_{k=1}^{p}\lambda_j\lambda_k\,|Z_{j,k}|^2 \\ &\geq \lambda_1^2\sum_{j=1}^{p}\sum_{k=1}^{p}|Z_{j,k}|^2 = \lambda_1^2\text{trace }Z^2 = \lambda_1^2\text{trace }{H_2}^2.\end{aligned}$$

This concludes the proof. □

Proof of Lemma 13

Proof. First we observe that for any fixed $\boldsymbol{\omega} \in \mathcal{T}^d$, $\overline{I}_{\mathbf{n}_k}(\boldsymbol{\omega};\boldsymbol{\gamma}_k)$ converges to $\overline{I}_{\mathbf{n}_k}(\boldsymbol{\omega};\boldsymbol{\gamma})$ as k goes to infinity. This comes from Assumption 1.(1b), where we have assumed an upper-bound on the derivative of the spectral density with respect to the parameter vector. In that case,

$$\begin{aligned}\left|\overline{I}_{\mathbf{n}_k}(\boldsymbol{\omega};\boldsymbol{\gamma}_k) - \overline{I}_{\mathbf{n}_k}(\boldsymbol{\omega};\boldsymbol{\gamma})\right| &\leq \left|(2\pi)^{-d}\int_{\mathcal{T}^d}\{f_{X,\boldsymbol{\delta}}(\boldsymbol{\omega}-\boldsymbol{\omega}';\boldsymbol{\gamma}_k) - f_{X,\boldsymbol{\delta}}(\boldsymbol{\omega}-\boldsymbol{\omega}';\boldsymbol{\gamma})\}\,\mathcal{F}_{\mathbf{n}}(\boldsymbol{\omega}')d\boldsymbol{\omega}'\right| \\ &\leq (2\pi)^{-d}\int_{\mathcal{T}^d}|f_{X,\boldsymbol{\delta}}(\boldsymbol{\omega}-\boldsymbol{\omega}';\boldsymbol{\gamma}_k) - f_{X,\boldsymbol{\delta}}(\boldsymbol{\omega}-\boldsymbol{\omega}';\boldsymbol{\gamma})|\,\mathcal{F}_{\mathbf{n}}(\boldsymbol{\omega}')d\boldsymbol{\omega}' \\ &\leq (2\pi)^{-d}\int_{\mathcal{T}^d}M_{\partial_\theta}\|\boldsymbol{\gamma}_k-\boldsymbol{\gamma}\|_2\mathcal{F}_{\mathbf{n}}(\boldsymbol{\omega}')d\boldsymbol{\omega}' \\ &\leq M_{\partial_\theta}\|\boldsymbol{\gamma}_k-\boldsymbol{\gamma}\|_2\end{aligned}$$

which converges to zero as $\|\boldsymbol{\gamma}_k-\boldsymbol{\gamma}\|_2$ converges to zero by assumption.

Now using equation (28), we can apply the Dominated Convergence Theorem to $(\widetilde{l}_{\mathbf{n}_k}(\boldsymbol{\gamma}_k) - \widetilde{l}_{\mathbf{n}_k}(\boldsymbol{\gamma}))_{k\in\mathbb{N}}$, using the bounds established in Lemma 10, and the $\boldsymbol{\omega}$-pointwise convergence of $\left|\overline{I}_{\mathbf{n}_k}(\boldsymbol{\omega};\boldsymbol{\gamma}_k) - \overline{I}_{\mathbf{n}_k}(\boldsymbol{\omega};\boldsymbol{\gamma}_k)\right|$ to zero. Hence $(\widetilde{l}_{\mathbf{n}_k}(\boldsymbol{\gamma}_k) - \widetilde{l}_{\mathbf{n}_k}(\boldsymbol{\gamma}))_{k\in\mathbb{N}}$ converges to zero, which concludes the proof. □

Proof of Lemma 14

Proof. Assume, with the intent to reach a contradiction, that $(\boldsymbol{\gamma}_k)$ does not converge to $\boldsymbol{\theta}$. By compactness of Θ, there exists $\boldsymbol{\gamma} \in \Theta$ distinct from $\boldsymbol{\theta}$ and $(\boldsymbol{\gamma}_{j_k})$ a subsequence of $(\boldsymbol{\gamma}_k)$ such that $\boldsymbol{\gamma}_{j_k}$ converges to $\boldsymbol{\gamma}$. We then have, using the inverse triangle inequality,

$$|\widetilde{l}_{\mathbf{n}_{j_k}}(\boldsymbol{\gamma}_{j_k}) - \widetilde{l}_{\mathbf{n}_{j_k}}(\boldsymbol{\theta})| \geq \left|\widetilde{l}_{\mathbf{n}_{j_k}}(\boldsymbol{\gamma}) - \widetilde{l}_{\mathbf{n}_{j_k}}(\boldsymbol{\theta})\right| - \left|\widetilde{l}_{\mathbf{n}_{j_k}}(\boldsymbol{\gamma}_{j_k}) - \widetilde{l}_{\mathbf{n}_{j_k}}(\boldsymbol{\gamma})\right|.$$

The second term on the right-hand side of the above equation converges to zero according to Lemma 13 whereas the first term is asymptotically lower bounded according to Lemma 11. Therefore the quantity $|\widetilde{l}_{\mathbf{n}_{j_k}}(\boldsymbol{\gamma}_{j_k}) - \widetilde{l}_{\mathbf{n}_{j_k}}(\boldsymbol{\theta})|$ is asymptotically lower bounded, which contradicts the initial assumption that $\widetilde{l}_{\mathbf{n}_k}(\boldsymbol{\gamma}_k) - \widetilde{l}_{\mathbf{n}_k}(\boldsymbol{\theta})$ converges to zero. This concludes the proof, by obtaining a contradiction. □

Proof of Lemma 15

Proof. We have, for $\boldsymbol{\gamma} \in \Theta$,

$$\widetilde{l}_{\mathbf{n}_k}(\boldsymbol{\gamma}) - l_{\mathbf{n}_k}(\boldsymbol{\gamma}) = |\mathbf{n}_k|^{-1} \sum_{\boldsymbol{\omega} \in \Omega_{\mathbf{n}}} \left\{ \log \overline{I}_{\mathbf{n}_k}(\boldsymbol{\omega}; \boldsymbol{\gamma}) + \frac{\overline{I}_{\mathbf{n}_k}(\boldsymbol{\omega}; \boldsymbol{\theta})}{\overline{I}_{\mathbf{n}_k}(\boldsymbol{\omega}; \boldsymbol{\gamma})} - \log \overline{I}_{\mathbf{n}_k}(\boldsymbol{\omega}; \boldsymbol{\gamma}) - \frac{I_{\mathbf{n}_k}(\boldsymbol{\omega})}{\overline{I}_{\mathbf{n}_k}(\boldsymbol{\omega}; \boldsymbol{\gamma})} \right\}$$
$$= |\mathbf{n}_k|^{-1} \sum_{\boldsymbol{\omega} \in \Omega_{\mathbf{n}}} \frac{\overline{I}_{\mathbf{n}_k}(\boldsymbol{\omega}; \boldsymbol{\theta}) - I_{\mathbf{n}_k}(\boldsymbol{\omega})}{\overline{I}_{\mathbf{n}_k}(\boldsymbol{\omega}; \boldsymbol{\gamma})}.$$

In order to show that $\widetilde{l}_{\mathbf{n}_k}(\boldsymbol{\gamma}) - l_{\mathbf{n}_k}(\boldsymbol{\gamma})$ converges uniformly in probability to the zero function over Θ, we need to show that,

$$\sup_{\boldsymbol{\gamma} \in \Theta} \left| \widetilde{l}_{\mathbf{n}_k}(\boldsymbol{\gamma}) - l_{\mathbf{n}_k}(\boldsymbol{\gamma}) \right| = o_p(1), \tag{14}$$

as k goes to infinity.

We first observe that, given that the quantity $\overline{I}_{\mathbf{n}_k}(\boldsymbol{\omega}; \boldsymbol{\gamma})^{-1}$ is deterministic and upper-bounded independently of $\boldsymbol{\gamma}$ by $f_{\min,\delta}^{-1}$, we can use Proposition 1 to write that

$$\operatorname{var}\left\{ \widetilde{l}_{\mathbf{n}_k}(\boldsymbol{\gamma}) - l_{\mathbf{n}_k}(\boldsymbol{\gamma}) \right\} = \mathcal{O}\left\{ \frac{\sum_{\mathbf{u} \in \mathbb{Z}^d} c_X(\mathbf{u})^2 c_g(\mathbf{u})}{\sum g_{\mathbf{s}}^2} \right\},$$

where the big $\mathcal{O}$ does not depend on $\boldsymbol{\gamma}$. Thus using Chebychev's inequality

$$\widetilde{l}_{\mathbf{n}_k}(\boldsymbol{\gamma}) - l_{\mathbf{n}_k}(\boldsymbol{\gamma}) = \mathcal{O}_P\left\{ \left(\frac{\sum_{\mathbf{u} \in \mathbb{Z}^d} c_X(\mathbf{u})^2 c_g(\mathbf{u})}{\sum g_{\mathbf{s}}^2} \right)^{1/2} \right\}.$$

This holds for any fixed $\boldsymbol{\gamma} \in \Theta$. In order to establish uniform convergence in probability we shall also use smoothness properties of the expected periodogram. Let $\epsilon > 0$ and $\eta > 0$. Define the events,

$$A_k = \left(\sup_{\boldsymbol{\gamma} \in \Theta} \left| \widetilde{l}_{\mathbf{n}_k}(\boldsymbol{\gamma}) - l_{\mathbf{n}_k}(\boldsymbol{\gamma}) \right| \geq \epsilon \right), \quad \forall k \in \mathbb{N}.$$

We wish to show that there exists $k_A \in \mathbb{N}$ such that for all integer $k \geq k_A, P(A_k) \leq \eta$. We note that,

$$A_k = \bigcup_{\boldsymbol{\gamma} \in \Theta} \left(\left| \widetilde{l}_{\mathbf{n}_k}(\boldsymbol{\gamma}) - l_{\mathbf{n}_k}(\boldsymbol{\gamma}) \right| \geq \epsilon \right), \quad \forall k \in \mathbb{N}.$$

Indeed, inclusion $\supset$ is obvious, whereas inclusion $\subset$ follows from the sup being reached due to the continuity w.r.t $\boldsymbol{\gamma}$ and the compacity of Θ. Let

$$\Delta_{\mathbf{n}_k}(\boldsymbol{\gamma}, \boldsymbol{\gamma}') = \widetilde{l}_{\mathbf{n}_k}(\boldsymbol{\gamma}) - l_{\mathbf{n}_k}(\boldsymbol{\gamma}) - (\widetilde{l}_{\mathbf{n}_k}(\boldsymbol{\gamma}') - l_{\mathbf{n}_k}(\boldsymbol{\gamma}')).$$

We have, by Taylor-expension,

$$\Delta_{\mathbf{n}_k}(\boldsymbol{\gamma}, \boldsymbol{\gamma}') = |\mathbf{n}_k|^{-1} \sum_{\boldsymbol{\omega} \in \Omega_{\mathbf{n}}} \left(\frac{1}{\overline{I}_{\mathbf{n}_k}(\boldsymbol{\omega}; \boldsymbol{\gamma})} - \frac{1}{\overline{I}_{\mathbf{n}_k}(\boldsymbol{\omega}; \boldsymbol{\gamma}')} \right) \left(\overline{I}_{\mathbf{n}_k}(\boldsymbol{\omega}; \boldsymbol{\theta}) - I_{\mathbf{n}_k}(\boldsymbol{\omega}) \right)$$
$$= |\mathbf{n}_k|^{-1} \sum_{\boldsymbol{\omega} \in \Omega_{\mathbf{n}}} \left\{ \frac{1}{\overline{I}_{\mathbf{n}_k}(\boldsymbol{\omega}; \boldsymbol{\gamma})^2} (\boldsymbol{\gamma}' - \boldsymbol{\gamma})^T \nabla_\theta \overline{I}_{\mathbf{n}_k}(\boldsymbol{\omega}; \widetilde{\boldsymbol{\gamma}}_{\boldsymbol{\omega}}) \left(\overline{I}_{\mathbf{n}_k}(\boldsymbol{\omega}; \boldsymbol{\theta}) - I_{\mathbf{n}_k}(\boldsymbol{\omega}) \right) \right\},$$

where each $\widetilde{\gamma}_{\boldsymbol{\omega}}$ depends on $\boldsymbol{\omega}$. Hence, by the triangle inequality,

$$|\Delta_{\mathbf{n}_k}(\gamma,\gamma')| \le |\mathbf{n}_k|^{-1}\left|\sum_{\boldsymbol{\omega}\in\Omega_{\mathbf{n}}}\frac{1}{\overline{I}_{\mathbf{n}_k}(\boldsymbol{\omega};\gamma)^2}\left(\gamma'-\gamma\right)^T\nabla_\theta\overline{I}_{\mathbf{n}_k}(\boldsymbol{\omega};\widetilde{\gamma}_{\boldsymbol{\omega}})I_{\mathbf{n}_k}(\boldsymbol{\omega})\right|$$
$$+|\mathbf{n}_k|^{-1}\left|\sum_{\boldsymbol{\omega}\in\Omega_{\mathbf{n}}}\frac{1}{\overline{I}_{\mathbf{n}_k}(\boldsymbol{\omega};\gamma)^2}\left(\gamma'-\gamma\right)^T\nabla_\theta\overline{I}_{\mathbf{n}_k}(\boldsymbol{\omega};\widetilde{\gamma}_{\boldsymbol{\omega}})\overline{I}_{\mathbf{n}_k}(\boldsymbol{\omega};\boldsymbol{\theta})\right|.$$

Using the upper-bound for the norm of the derivative of the spectral density with respect to the parameter vector, as well as the lower bound for the spectral density, we obtain,

$$|\Delta_{\mathbf{n}_k}(\gamma,\gamma')| \le |\mathbf{n}_k|^{-1}f_{\delta,\min}^{-2}M_{\partial\theta}\|\gamma'-\gamma\|\sum_{\boldsymbol{\omega}\in\Omega_{\mathbf{n}}}\left\{I_{\mathbf{n}_k}(\boldsymbol{\omega})+\overline{I}_{\mathbf{n}_k}(\boldsymbol{\omega};\boldsymbol{\theta})\right\}$$
$$=|\mathbf{n}_k|^{-1}f_{\delta,\min}^{-2}M_{\partial\theta}\|\gamma'-\gamma\|\left(2\sum_{\boldsymbol{\omega}\in\Omega_{\mathbf{n}}}\overline{I}_{\mathbf{n}_k}(\boldsymbol{\omega};\boldsymbol{\theta})+o_P(1)\right),$$

according to Proposition 1, and using SCC. This implies that we can choose $\delta>0$ small enough such that there exists a natural integer k_C such that,

$$\forall k\ge k_C,\ \forall\boldsymbol{\gamma},\boldsymbol{\gamma}'\in\Theta,\ \|\gamma'-\gamma\|\le\delta\Longrightarrow P\left(|\Delta_{\mathbf{n}_k}(\gamma,\gamma')|\ge\frac{\epsilon}{2}\right)\le\frac{\eta}{2.}.$$

Now, let $\left\{\boldsymbol{\alpha}_j^\delta\right\}_{j=1,\ldots,J}$ be a finite family of elements of Θ such that,

$$\bigcup_{j=1}^{J}B(\boldsymbol{\alpha}_j^\delta,\delta)\supset\Theta,$$

with $B(\boldsymbol{\alpha}_j^\delta,\delta)$ denoting the ball centered on $\boldsymbol{\alpha}_j^\delta$ with radius δ. Existence here follows from the compacity of Θ, and the positiveness of δ. Define the events,

$$B_k=\bigcup_{j=1}^{J}\left(|\mathbf{n}_k|^{-1}\left|\sum_{\boldsymbol{\omega}\in\Omega_{\mathbf{n}}}\frac{\overline{I}_{\mathbf{n}_k}(\boldsymbol{\omega};\boldsymbol{\theta})-I_{\mathbf{n}_k}(\boldsymbol{\omega})}{\overline{I}_{\mathbf{n}_k}(\boldsymbol{\omega};\boldsymbol{\alpha}_j)}\right|\ge\frac{\epsilon}{2}\right),\quad\forall k\in\mathbb{N},$$

and $C_k=A_k\backslash B_k$. Clearly $B_k\subset A_k$ so that $A_k=B_k\cup C_k$, and therefore $P(A_k)\le P(B_k)+P(C_k)$. Again by Proposition 1, and because J is finite, there exists k_B such that for any integer $k\ge k_B$, $P(B_k)\le\frac{\eta}{2}$. Finally, for an outcome in C_k, there exists $\boldsymbol{\gamma}'\in\Theta$ such that $\left|\widetilde{l}_{\mathbf{n}_k}(\gamma')-l_{\mathbf{n}_k}(\gamma')\right|\ge\epsilon$. By construction, there exists $j\in\{1,\ldots,J\}$ such that $\|\boldsymbol{\alpha}_j-\boldsymbol{\gamma}'\|\le\delta$, but at the same time we have $\left|\widetilde{l}_{\mathbf{n}_k}(\boldsymbol{\alpha}_j)-l_{\mathbf{n}_k}(\boldsymbol{\alpha}_j)\right|\le\frac{\epsilon}{2}$. By inverse triangle inequality, we therefore have, $\Delta_{\mathbf{n}}(\boldsymbol{\alpha}_j,\gamma')\ge\frac{\epsilon}{2}$. Hence for integer $k\ge k_C$, $P(C_k)\le\frac{\eta}{2}$. We conclude that, with $k_A=\max(k_B,k_c)$, for $k\ge k_A, P(A_k)\le\eta$. Since this can be achieved for any choice of η, this concludes the proof.

The extension to univariate non-Gaussian random fields follows from Corollary 1. Similarily, for a Gaussian multivariate random field,

$$\widetilde{l}_{\mathbf{n}_k}(\gamma)-l_{\mathbf{n}_k}(\gamma)=|\mathbf{n}_k|^{-1}\sum_{\boldsymbol{\omega}\in\Omega_{\mathbf{n}}}\left\{\operatorname{trace}\left[\overline{I}_{\mathbf{n}_k}(\boldsymbol{\omega};\boldsymbol{\theta})\overline{I}_{\mathbf{n}_k}(\boldsymbol{\omega};\gamma)^{-1}\right]-J^H(\boldsymbol{\omega})\overline{I}_{\mathbf{n}_k}(\boldsymbol{\omega};\gamma)^{-1}J(\boldsymbol{\omega})\right\}$$

and we use Corollary 2. □

Proof of Lemma 16

Proof. The proof is adapted from the one-dimensional case, see Guillaumin *et al.* (2017) and Sykulski *et al.* (2019). We first define the following isomorphism from $\prod_{i=1}^{d}\{1,\dots,n_i\}$ to $\{1,\dots,|\mathbf{n}|\}$, that will be used for a change of variable:

$$j(j_1,\dots,j_d)=\sum_{k=1}^{d}\left\{(j_k-1)\prod_{j=1}^{k-1}n_j\right\},$$

and $j_1(j),\dots,j_d(j)$ the component functions of its inverse. This isomorphism gives the index in the column vector $\mathbf{X}$ of the observation at location $(j_1,\dots,j_d)$ on the grid, given our choice of ordering.

Let $\boldsymbol{\alpha}$ be any complex-valued vector of $\mathbb{C}^n$, and denote $\boldsymbol{\alpha}^*$ its Hermitian transpose. We then have, using the above isomorphism for a change of variables,

$$\begin{aligned}\boldsymbol{\alpha}^* C_{\mathbf{X}}\boldsymbol{\alpha} &= \sum_{j,k=1}^{|\mathbf{n}|}\boldsymbol{\alpha}_j^*(C_{\mathbf{X}})_{j,k}\boldsymbol{\alpha}_k\\ &=\sum_{j_1=0}^{n_1-1}\cdots\sum_{j_d=1}^{n_d-1}\sum_{k_1=0}^{n_1-1}\cdots\sum_{k_d=1}^{n_d-1}\boldsymbol{\alpha}^*_{j(j_1,\dots,j_d)}(C_{\mathbf{X}})_{j(j_1,\dots,j_d),k(k_1,\dots,k_d)}\boldsymbol{\alpha}_{k(k_1,\dots,k_d)}.\end{aligned}$$

Here we use the fact that

$$(C_{\mathbf{X}})_{j(j_1,\dots,j_d),k(k_1,\dots,k_d)}=c_{\mathbf{X}}(k_1-j_1,\dots,k_d-j_d),$$

so that

$$\begin{aligned}\boldsymbol{\alpha}^* C_{\mathbf{X}}\boldsymbol{\alpha} &= \sum_{j_1=0}^{n_1-1}\cdots\sum_{j_d=1}^{n_d-1}\sum_{k_1=0}^{n_1-1}\cdots\sum_{k_d=1}^{n_d-1}\boldsymbol{\alpha}^*_{j(j_1,\dots,j_d)}\boldsymbol{\alpha}_{k(k_1,\dots,k_d)}\int_{\mathcal{T}^d}f_{X,\delta}(\boldsymbol{\omega})e^{i((k_1-j_1)\omega_1+\dots+(k_d-j_d)\omega_d)}d\boldsymbol{\omega}\\ &=\int_{\mathcal{T}^d}f_{X,\delta}(\boldsymbol{\omega})\sum_{j_1=0}^{n_1-1}\cdots\sum_{j_d=1}^{n_d-1}\sum_{k_1=0}^{n_1-1}\cdots\sum_{k_d=1}^{n_d-1}\boldsymbol{\alpha}^*_{j(j_1,\dots,j_d)}\boldsymbol{\alpha}_{k(k_1,\dots,k_d)}e^{i((k_1-j_1)\omega_1+\dots+(k_d-j_d)\omega_d)}d\boldsymbol{\omega}\\ &=\int_{\mathcal{T}^d}f_{X,\delta}(\boldsymbol{\omega})\left|\sum_{j_1=0}^{n_1-1}\cdots\sum_{j_d=1}^{n_d-1}\boldsymbol{\alpha}_{j(j_1,\dots,j_d)}e^{i(j_1\omega_1+\dots+j_d\omega_d)}\right|^2 d\boldsymbol{\omega}\\ &\le f_{\delta,\max}\int_{\mathcal{T}^d}\left|\sum_{j_1=0}^{n_1-1}\cdots\sum_{j_d=1}^{n_d-1}\boldsymbol{\alpha}_{j(j_1,\dots,j_d)}e^{i(j_1\omega_1+\dots+j_d\omega_d)}\right|^2 d\boldsymbol{\omega}.\end{aligned}$$

By Parseval's equality, we obtain,

$$0\le\boldsymbol{\alpha}^* C_{\mathbf{X}}\boldsymbol{\alpha}\le f_{\delta,\max}\|\boldsymbol{\alpha}\|_2^2,$$

where $\|\boldsymbol{\alpha}\|_2$ is the l_2 vector norm of the vector $\boldsymbol{\alpha}$. This concludes the proof of the upper bound. The lower bound can be derived in the same way, which concludes the proof. □

Proof of Proposition 2

Proof. We only treat the scenario where $g_{\mathbf{s}}=1, \forall\mathbf{s}\in\mathcal{J}_{n_k}$, i.e., we do not consider the situation of missing observations for this proposition. The proof is adapted from Grenander and

Szegö (1958, p. 217). We write it for the case of $\Omega_{\mathbf{n}} = \Omega_{\mathbf{n}}^{(1)}$, the case $\Omega_{\mathbf{n}} = \Omega_{\mathbf{n}}^{(2)}$ being almost identical. Define

$$L_k = |\mathbf{n}_k|^{-1} \sum_{\boldsymbol{\omega} \in \Omega_{\mathbf{n}_k}} w_k(\boldsymbol{\omega}) I_{\mathbf{n}_k}(\boldsymbol{\omega}),$$

as a weighted sum of periodogram values, and $U_{\mathbf{n}_k}$ the multi-dimensional Fourier matrix corresponding to $\mathcal{J}_{\mathbf{n}}$. We have

$$L_k = |\mathbf{n}_k|^{-1} \mathbf{X}^* U_{\mathbf{n}_k}^* \mathrm{diag}(w_k(\boldsymbol{\omega}_0), \ldots, w_k(\boldsymbol{\omega}_{|\mathbf{n}_k|-1})) U_{\mathbf{n}_k} \mathbf{X}.$$

Writing $W_k = |\mathbf{n}_k|^{-1} U_{\mathbf{n}_k}^* \mathrm{diag}(w_k(\boldsymbol{\omega}_0), \ldots, w_k(\boldsymbol{\omega}_{|\mathbf{n}_k|-1})) U_{\mathbf{n}_k}$, we then have

$$L_k = \mathbf{X}^* W_k \mathbf{X},$$

which we regard as a quadratic form in the vector $\mathbf{X}$. Following Cramér (1946, p. 134), in particular his formula 11.12.2, the characteristic function of the random variable L_k therefore takes the form of

$$\begin{aligned} \phi_{L_k}(\alpha) &= \mathrm{E}\left\{\exp(i\alpha L_k)\right\} \\ &= (2\pi)^{-n/2} \left|C_X(\boldsymbol{\theta})\right|^{-1/2} \int_{-\infty}^{\infty} \cdots \int_{-\infty}^{\infty} \exp\left\{-x^* \left(-i\alpha W_k + \frac{1}{2} C_X^{-1}(\boldsymbol{\theta})\right) x\right\} dx_1 \ldots dx_n, \end{aligned}$$

where for a square matrix A, $|A|$ denotes its determinant. Using a known result (Horn and Johnson, 1985) for complex-valued symmetric matrices, there exists a diagonal matrix D_k and a unitary matrix V_k such that

$$-i\alpha W_k + \frac{1}{2} C_X^{-1}(\boldsymbol{\theta}) = V D_k V^T. \tag{15}$$

By posing the change of variables $y = V^T x$ we obtain,

$$\phi_{L_k}(\alpha) = (2\pi)^{-n/2} \left|C_X(\boldsymbol{\theta})\right|^{-1/2} \prod_{j=1}^{n} \int_{-\infty}^{\infty} \exp\left\{-y^2 d_{j,k}\right\} dy,$$

where the $d_{j,k}, j = 1, \ldots, n$ are the complex-valued elements of the diagonal matrix D_k from equation (15), and where we remind the reader that $|V| = 1$ since V is unitary. As we recognize integrals of the form $\int_{-\infty}^{\infty} \exp(-y^2) dy$ we obtain,

$$\begin{aligned} \phi_{L_k}(\alpha) &= 2^{-n/2} \left|C_X(\boldsymbol{\theta})\right|^{-1/2} \left|-i\alpha W_k + \frac{1}{2} C_X(\boldsymbol{\theta})^{-1}\right|^{-1/2} \\ &= \left|-2i\alpha C_X(\boldsymbol{\theta}) W_k + I_{|\mathbf{n}|}\right|^{-1/2} \end{aligned}$$

Hence,

$$\log \phi_{L_k}(\alpha) = -\frac{1}{2} \log \left|I_{|\mathbf{n}_k|} - 2i\alpha C_X(\boldsymbol{\theta}) W_k\right|.$$

Denoting with $\nu_{1,k}, \ldots, \nu_{|\mathbf{n}_k|,k}$ the eigenvalues of $C_X(\boldsymbol{\theta}) W_k$, we therefore have

$$\log \phi_{L_k}(\alpha) = -\frac{1}{2} \sum_{j=1}^{|\mathbf{n}_k|} \log\left(1 - 2i\alpha \nu_{j,k}\right).$$

According to Proposition 16 the spectral norm of C_X, the covariance matrix of $\mathbf{X}$, is upper-bounded by $f_{\max,\delta}$. The spectral norm of W_k is clearly upper-bounded by $|\mathbf{n}_k|^{-1}M_W$, as from the definition of W_k its eigenvalues are exactly

$$|\mathbf{n}_k|^{-1}w_k(\boldsymbol{\omega}_0), |\mathbf{n}_k|^{-1}w_k(\boldsymbol{\omega}_1), \ldots, |\mathbf{n}_k|^{-1}w_k(\boldsymbol{\omega}_{|\mathbf{n}_k|-1}).$$

By property of the spectral norm of a product of matrices, we obtain,

$$|\mathbf{n}_k|^{-1}m_W f_{\min,\delta} \le |\nu_{j,k}| \le |\mathbf{n}_k|^{-1}M_W f_{\max,\delta}, \quad \forall j = 1, \ldots, |\mathbf{n}_k|, k \in \mathbb{N}. \tag{16}$$

The variance of L_k is given by

$$\sigma_k^2 = \text{var}\{L_k\} = 2\sum_{j=1}^{|\mathbf{n}_k|} \nu_{j,k}^2,$$

and therefore satisfies

$$2|\mathbf{n}_k|^{-1}(m_W f_{\min})^2 \le \sigma_k^2 \le 2|\mathbf{n}_k|^{-1}(M_W f_{\max,\delta})^2. \tag{17}$$

We also observe that

$$\frac{\nu_{j,k}}{\sigma_k} \to 0, \ (k \to \infty),$$

uniformly, given the bounds determined in equations (16) and (17). Denote $\underline{L}_k$ the standardized quantity $(L_k - \text{E}\{L_k\})/\sigma_k$. After Taylor expansion of the logarithm terms to third order, its characteristic function takes the form of

$$\begin{aligned}\log \phi_{\underline{L}_k}(\alpha) &= -\frac{1}{2}\sum_{j=1}^{|\mathbf{n}_k|} \log\left(1 - \frac{2i\alpha\nu_{j,k}}{\sigma_k}\right) - i\frac{\alpha\sum_{j=1}^{|\mathbf{n}_k|}\nu_{j,k}}{\sigma_k} \\ &= -\frac{1}{2}\alpha^2 + \sum_{j=1}^{|\mathbf{n}_k|}\left[\frac{4}{3}\left(\frac{i\alpha\nu_{j,k}}{\sigma_k}\right)^3 + o\left\{\left(\frac{i\alpha\nu_{j,k}}{\sigma_k}\right)^3\right\}\right],\end{aligned} \tag{18}$$

where the small o is uniform and is denoted ϵ_k in what follows, to make it clear that it does not depend on j. The second term in equation (18) can be shown to become negligible as k goes to infinity, since

$$\begin{aligned}\left|\sum_{j=1}^{|\mathbf{n}_k|}\left[\frac{4}{3}\left(\frac{i\alpha\nu_{j,k}}{\sigma_k}\right)^3 + o\left\{\left(\frac{i\alpha\nu_{j,k}}{\sigma_k}\right)^3\right\}\right]\right| &\le \alpha^3\sigma_k^{-3}\left(\frac{4}{3}+\epsilon_k\right)\sum_{j=1}^{|\mathbf{n}_k|}|\nu_{j,k}|^3 \\ &\le \alpha^3\left(\frac{4}{3}+\epsilon_k\right)\frac{|\mathbf{n}_k|^{-2}M_W^3 f_{\max}^3}{|\mathbf{n}_k|^{-3/2}m_W^3 f_{\min}^3} \\ &= \mathcal{O}(|\mathbf{n}_k|^{-1/2}).\end{aligned}$$

We conclude that $\phi_{\underline{L}_k}(\alpha)$ converges to $\exp(-\frac{1}{2}\alpha)$, and therefore L_k is asymptotically standard normally distributed after appropriate normalization. □

Proof of Theorem 2

Proof. Direct calculations show that the gradient of our quasi-likelihood function at the true parameter vector is given by,

$$\nabla_\theta l_{\mathbf{n}_k}(\boldsymbol{\theta}) = |\mathbf{n}_k|^{-1}\sum_{\boldsymbol{\omega}\in\Omega_{\mathbf{n}_k}} \overline{I}_{\mathbf{n}_k}(\boldsymbol{\omega};\boldsymbol{\theta})^{-2}\left(\overline{I}_{\mathbf{n}_k}(\boldsymbol{\omega};\boldsymbol{\theta}) - I(\boldsymbol{\omega})\right)\nabla_\theta \overline{I}_{\mathbf{n}_k}(\boldsymbol{\omega};\boldsymbol{\theta}). \tag{19}$$

By expanding this gradient function at the true parameter value, and noting that $\nabla_\theta l_{\mathbf{n}_k}(\boldsymbol{\omega};\hat{\boldsymbol{\theta}})=0$ by definition of $\hat{\boldsymbol{\theta}}$ and given Assumption 2.(2a), we obtain

$$\nabla_\theta l_{\mathbf{n}_k}(\boldsymbol{\omega};\boldsymbol{\theta}) = H(\boldsymbol{\theta}_k')(\boldsymbol{\theta}-\hat{\boldsymbol{\theta}}_k),$$

where $H(\cdot)$ is the Hessian of $l_{\mathbf{n}_k}(\cdot)$ and $\boldsymbol{\theta}_k'$ is a parameter vector that converges in probability to the true parameter vector, since $\hat{\boldsymbol{\theta}}_k$ is consistent as per Theorem 1. Therefore,

$$\hat{\boldsymbol{\theta}}_k - \boldsymbol{\theta} = -H^{-1}(\boldsymbol{\theta}_k')\nabla_\theta l_{\mathbf{n}_k}(\boldsymbol{\omega};\boldsymbol{\theta}). \tag{20}$$

We now study the expected Hessian of the likelihood function taken at the true parameter vector, $\mathcal{H}(\boldsymbol{\theta})$. Direct calculations lead to

$$\mathcal{H}(\boldsymbol{\theta}) = |\mathbf{n}_k|^{-1}\sum_{\boldsymbol{\omega}\in\Omega_{\mathbf{n}_k}} \overline{I}_{\mathbf{n}_k}(\boldsymbol{\omega};\boldsymbol{\theta})^{-2}\nabla_\theta \overline{I}_{\mathbf{n}_k}(\boldsymbol{\omega};\boldsymbol{\theta})\nabla_\theta \overline{I}_{\mathbf{n}_k}(\boldsymbol{\omega};\boldsymbol{\theta})^T.$$

It can be shown, see Sykulski *et al.* (2019, p. 17 of their supplementary document) for instance, that in equation (20) the quantity $H(\boldsymbol{\theta}_k')$ satisfies, if Assumption 2.(2b) holds,

$$H(\boldsymbol{\theta}_k') = \mathcal{H}(\boldsymbol{\theta}) + \mathcal{O}_P(r_k) + o_P(1).$$

Hence we have, asymptotically,

$$H^{-1}(\boldsymbol{\theta}_k') = \mathcal{H}^{-1}(\boldsymbol{\theta}) + o_P(1). \tag{21}$$

Since equation (19) follows the conditions required for Proposition 1 to apply, the gradient at the true parameter vector $\nabla_\theta l_{\mathbf{n}_k}(\boldsymbol{\omega};\boldsymbol{\theta})$ is itself $\mathcal{O}_P(r_k)$. Further more, Lemma 17 tells us that the minimum eigenvalue of $\mathcal{H}$ is lower-bounded by $S(\boldsymbol{\theta})$, independently of k. We finally obtain the stated result,

$$\hat{\boldsymbol{\theta}}_k - \boldsymbol{\theta} = \mathcal{O}_P(r_k).$$

In the case of a sequence of full grids, $|\mathbf{n}_k|^{1/2}\nabla_\theta l_{\mathbf{n}_k}(\boldsymbol{\omega};\boldsymbol{\theta})$ is additionally shown to follow a standard normal distribution via Proposition 2, and we conclude to the asymptotic normality of our estimator. □

Definitions, notation and lemmatas required for the proof of Proposition 3

First we introduce some notation for cumulants and remind the reader about their basic properties. For integer $L \geq 1$ and random variables $Y_1,\dots,Y_L$, all having finite L-th order moments, the cumulant of $Y_1,\dots,Y_L$ is defined by,

$$\operatorname{cum}[Y_1,\dots,Y_L] = \sum_{\nu\in\mathcal{P}\{1,\dots,L\}} (-1)^{\#\nu-1}(\#\nu-1)!\prod_{\mathcal{S}\in\nu}\mathrm{E}\left[\prod_{j\in\mathcal{S}} Y_j\right],$$

where $\mathcal{P}\{1,\dots,L\}$ denotes the set of partitions of $\{1,\dots,L\}$, and $\#\nu$ denotes the cardinality of the partition ν, i.e. the number of sets it contains. The cases $L=1$ and $L=2$ correspond to expectation and covariance respectively. Higher-order cumulants vanish for multivariate normal $Y_1,\dots,Y_L$. For a given random variable Y, we denote $\operatorname{cum}_L\{Y\}$ its L-th order cumulant, i.e. $\operatorname{cum}_L\{Y\} = \operatorname{cum}(Y,\dots,Y)$ with Y repeated L times. In our proof we shall make use of the two following lemmas, that can be found in Brillinger (2001).

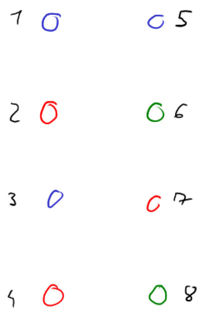

Fig. 6. Example of an indecomposable partition of the $L \times 2$ table (22) in the case $L = 4$, with sets of the partition indicated by colors red, green and blue. The partition is indecomposable because any two elements of the table can be joined by a path where two consecutive elements on said path are either within a same set $\mathcal{S} \in \nu$ or on the same row. For instance, here, such a path between 1 and 8 is $1 \rightarrow 3 \rightarrow 7 \rightarrow 4 \rightarrow 8$.

Lemma B (Basic properties of cumulants). Let L be a positive integer, Z, $Y_1, \dots Y_L$ be random variables all having finite L-th order moments, and $a \in \mathbb{R}$. We have the following properties;

(a) *Symmetry.* The cumulant $\text{cum}\,\{Y_1, \dots, Y_L\}$ does not depend on the order of the variables.
(b) *Multi-linearity.* The cumulant is linear with respect to each of its variables, i.e.

$$\text{cum}\,\{aZ + Y_1, Y_2, \dots, Y_L\} = a\;\text{cum}\,\{Z, Y_2 \dots, Y_L\} + \text{cum}\,\{Y_1, \dots, Y_L\}\,.$$

Lemma C (Cumulant of products of random variables). Let L be a positive integer. Let $Y_1, \dots, Y_{2L}$ be random variables, all having finite $2L$-th order moments. We have,

$$\text{cum}\,[Y_1 Y_{L+1}, Y_2 Y_{L+2}, \dots, Y_L Y_{2L}] = \sum_{\nu} \text{cum}\,[Y_j : j \in \nu_1] \dots \text{cum}\,[Y_j : j \in \nu_p]\,,$$

where the left-hand side is the cumulant of L products of pairs of random variables, and where the summation on the right-hand side is over indecomposable—as defined by Brillinger (2001)—partitions $\nu = (\nu_1, \dots, \nu_p)$ of the $L \times 2$ table below.

$$\begin{array}{rr} 1 & L+1 \\ 2 & L+2 \\ \dots & \dots \\ L-1 & 2L-1 \\ L & 2L \end{array} \qquad (22)$$

A partition ν of the above $L \times 2$ table is indecomposable if and only if any two elements of the table can be joined by a path where two consecutive elements on said path are either within a same set $\mathcal{S} \in \nu$ or on the same row. We give an example of an indecomposable partition in Figure 6.

To establish the proof of Proposition 3 we shall follow the line of proof from Brillinger (2001) for the analysis of time series. We introduce some additional notation in order to extend to

random fields of any dimensionality d. Let $\Delta_{\mathbf{n}}(\boldsymbol{\omega})$ denote the d-dimensional separable Dirichlet kernel, i.e.,

$$\Delta_{\mathbf{n}}(\boldsymbol{\omega}) = \prod_{j=1}^{d} \left(\sum_{t=0}^{n_j - 1} e^{i\omega_j t} \right) = \prod_{j=1}^{d} \Delta_{n_j}(\omega_j),$$

where for a positive integer n, and scalar $\omega \in \mathbb{R}$,

$$\Delta_n(\omega) = \sum_{t=0}^{n-1} e^{i\omega t},$$

is the usual Dirichlet kernel. We define, for $0 \leq q \leq d$, and for any $l_1, \ldots, l_q \in \{1, \ldots, d\}$,

$$\Delta_{\mathbf{n}}^{(l_1,\ldots,l_q)}(\boldsymbol{\omega}) = \prod_{\substack{j=1 \\ j \neq l_1,\ldots,l_q}}^{d} \Delta_{n_j}(\omega_j). \tag{23}$$

Note that when $\boldsymbol{\omega} \in \Omega_{\mathbf{n}}$, i.e. is a Fourier frequency, $\Delta_{\mathbf{n}}(\boldsymbol{\omega}) = \Delta_{\mathbf{n}}^{(k)}(\boldsymbol{\omega}) = 0$ except if $\omega_j \equiv 0$ $[2\pi]$, $j = 1, \ldots, d$ (where we write $a \equiv b$ $[c]$ for real numbers a, b, c if there exists an integer k such that $a - b = kc$) in which case $\Delta(\boldsymbol{\omega}) = |\mathbf{n}|$, and except if $\omega_j \equiv 0$ $[2\pi]$, $j = 1, \ldots, d$, $j \neq k$ in which case $\Delta_{\mathbf{n}}^{(k)}(\boldsymbol{\omega}) = \prod_{\substack{j=1 \\ j \neq k}}^{d} n_j$.

Let $\widetilde{J}_{\mathbf{n}}(\boldsymbol{\omega}) = |\mathbf{n}|^{\frac{1}{2}} J_{\mathbf{n}}(\boldsymbol{\omega})$. The following lemma is an adaptation of Brillinger (2001, Lemma P4.1) to higher dimensions.

Lemma D. Let $d \geq 1$ be an integer. Let $\mathbf{n} \in (\mathbb{N} \backslash \{0\})^d$, $\mathbf{u} \in \mathbb{N}^d$ and $\boldsymbol{\lambda} \in \mathbb{R}^d$. Let $\{g_{\mathbf{s}}\}_{\mathbf{s} \in \mathbb{Z}^d}$ take value 1 for $\mathbf{s} \in \mathcal{J}_{\mathbf{n}}$, and value 0 otherwise. We have the following inequality,

$$\left| \sum_{\mathbf{s} \in \mathbb{Z}^d} g_{\mathbf{s}} g_{\mathbf{s}+\mathbf{u}} e^{-i\mathbf{s} \cdot \boldsymbol{\lambda}} - \Delta_{\mathbf{n}}(\boldsymbol{\lambda}) \right| \leq \sum_{j=1}^{d} u_j \left| \Delta_{\mathbf{n}}^{(j)}(\boldsymbol{\lambda}) \right| + \sum_{\substack{j,k=1 \\ k>j}}^{d} u_j u_k \left| \Delta_{\mathbf{n}}^{(j,k)}(\boldsymbol{\lambda}) \right| + \ldots + u_1 \cdots u_d \left| \Delta_{\mathbf{n}}^{(1,\ldots,d)}(\boldsymbol{\lambda}) \right|,$$

where we note that $\Delta_{\mathbf{n}}^{(1,\ldots,d)}(\boldsymbol{\lambda}) = 1$, $\forall \boldsymbol{\lambda} \in \mathbb{R}^d$.

Proof. We write,

$$\left| \sum_{\mathbf{s} \in \mathbb{Z}^d} g_{\mathbf{s}} g_{\mathbf{s}+\mathbf{u}} e^{-i\mathbf{s} \cdot \boldsymbol{\lambda}} - \Delta_{\mathbf{n}}(\boldsymbol{\lambda}) \right| = \left| \sum_{\mathbf{s} \in \mathbb{Z}^d} g_{\mathbf{s}} g_{\mathbf{s}+\mathbf{u}} e^{-i\boldsymbol{\lambda} \cdot \mathbf{s}} - \sum_{\mathbf{s} \in \mathbb{Z}^d} g_{\mathbf{s}} e^{-i\boldsymbol{\lambda} \cdot \mathbf{s}} \right| = \left| \sum_{\mathbf{s} \in \mathbb{Z}^d} g_{\mathbf{s}} (1 - g_{\mathbf{s}+\mathbf{u}}) e^{-i\boldsymbol{\lambda} \cdot \mathbf{s}} \right|.$$

We first consider the cases $d = 1$ and $d = 2$ as examples, before proving the result for any dimensionality $d \geq 1$ by induction. For $d = 1$, we have $u \in \mathbb{N}$ and, by applying the triangle inequality,

$$\left| \sum_{s} g_s (1 - g_{s+u}) e^{-i\lambda s} \right| \leq \sum_{s} \left| g_s (1 - g_{s+u}) e^{-i\lambda s} \right| = \sum_{s} |g_s| \, |(1 - g_{s+u})| = u.$$

The last equality holds because each term $|g_s| \, |(1 - g_{s+u})|$ is non-zero if and only if s is a point on the grid but $s + u$ is not, which occurs for a total number of u locations — more specifically

for $s \in \mathbb{Z}$ such that $n_1 - u \leq s \leq n_1 - 1$. In dimension $d = 2$, we *split* the problem along both dimensions.

$$\left|\sum_{\mathbf{s}\in\mathbb{Z}^2} g_{\mathbf{s}} g_{\mathbf{s}+\mathbf{u}} e^{-i\boldsymbol{\lambda}\cdot\mathbf{s}} - \Delta_n(\boldsymbol{\lambda})\right| = \left|\sum_{\mathbf{s}} g_{\mathbf{s}}(1 - g_{\mathbf{s}+\mathbf{u}}) e^{-i\boldsymbol{\lambda}\cdot\mathbf{s}}\right|$$
$$= \left|\sum_{s_1=0}^{n_1-1}\sum_{s_2=0}^{n_2-1}(1 - g_{s_1+u_1,s_2+u_2})e^{-i(\lambda_1 s_1+\lambda_2 s_2)}\right|$$
$$= \left|\sum_{s_1=0}^{n_1-1}\sum_{s_2=n_2-u_2}^{n_2-1} e^{-i(\lambda_1 s_1+\lambda_2 s_2)} + \sum_{s_2=0}^{n_2}\sum_{s_1=n_1-u_1}^{n_1-1} e^{-i(\lambda_1 s_1+\lambda_2 s_2)} - \sum_{s_2=n_2-u_2}^{n_2-1}\sum_{s_1=n_1-u_1}^{n_1-1} e^{-i(\lambda_1 s_1+\lambda_2 s_2)}\right|,$$

where we split the sum over non-zero terms, using the fact that,

$$\left\{(s_1, s_2) \in \mathbb{Z}^2 : g_{s_1,s_2}(1 - g_{s_1+u_1,s_2+u_2}) = 1\right\} =$$
$$\left\{(s_1, s_2) \in \mathbb{Z}^2 : 0 \leq s_1 < n_1\right\} \cap \left\{(s_1, s_2) \in \mathbb{Z}^2 : 0 \leq s_2 < n_2\right\} \cap$$
$$\left(\left\{(s_1, s_2) \in \mathbb{Z}^2 : s_1 \geq n_1 - u_1\right\} \cup \left\{(s_1, s_2) \in \mathbb{Z}^2 : s_2 \geq n_2 - u_2\right\}\right),$$

and that

$$\sum_{A\cup B} = \sum_{A} + \sum_{B} - \sum_{A\cap B}.$$

Then by applying the triangle inequality we obtain,

$$\left|\sum_{s} g_{\mathbf{s}} g_{\mathbf{s}+\mathbf{u}} e^{-i\boldsymbol{\lambda}\cdot\mathbf{s}} - \Delta_n(\boldsymbol{\lambda})\right|$$
$$\leq \left|\sum_{s_1=0}^{n_1-1}\sum_{s_2=n_2-u_2}^{n_2-1} e^{-i(\lambda_1 s_1+\lambda_2 s_2)}\right| + \left|\sum_{s_2=0}^{n_2-1}\sum_{s_1=n_1-u_1}^{n_1-1} e^{-i(\lambda_1 s_1+\lambda_2 s_2)}\right| + \left|\sum_{s_2=n_2-u_2}^{n_2-1}\sum_{s_1=n_1-u_1}^{n_1} e^{-i(\lambda_1 s_1+\lambda_2 s_2)}\right|$$
$$= \left|\sum_{s_2=n_2-u_2}^{n_2-1} e^{-i\lambda_2 s_2}\sum_{s_1=0}^{n_1-1} e^{-i\lambda_1 s_1}\right| + \left|\sum_{s_1=n_1-u_1}^{n_1-1} e^{-i\lambda_1 s_1}\sum_{s_2=0}^{n_2-1} e^{-i\lambda_2 s_2}\right| + \left|\sum_{s_2=n_2-u_2}^{n_2-1}\sum_{s_1=n_1-u_1}^{n_1} e^{-i(\lambda_1 s_1+\lambda_2 s_2)}\right|$$
$$= \left|\Delta_{n_1}(\lambda_1)\sum_{s_2=n_2-u_2}^{n_2-1} e^{-i\lambda_2 s_2}\right| + \left|\Delta_{n_2}(\lambda_2)\sum_{s_1=n_1-u_1}^{n_1-1} e^{-i\lambda_1 s_1}\right| + \left|\sum_{s_2=n_2-u_2}^{n_2-1}\sum_{s_1=n_1-u_1}^{n_1} e^{-i(\lambda_1 s_1+\lambda_2 s_2)}\right|$$
$$\leq |\Delta_{n_1}(\lambda_1)|\sum_{s_2=n_2-u_2}^{n_2-1}\left|e^{-i\lambda_2 s_2}\right| + |\Delta_{n_2}(\lambda_2)|\sum_{s_1=n_1-u_1}^{n_1-1}\left|e^{-i\lambda_1 s_1}\right| + \sum_{s_2=n_2-u_2}^{n_2-1}\sum_{s_1=n_1-u_1}^{n_1}\left|e^{-i(\lambda_1 s_1+\lambda_2 s_2)}\right|$$
$$= u_2|\Delta_{n_1}(\lambda_1)| + u_1|\Delta_{n_2}(\lambda_2)| + u_1u_2 = u_1\left|\Delta_{\mathbf{n}}^{(1)}(\boldsymbol{\lambda})\right| + u_2\left|\Delta_{\mathbf{n}}^{(2)}(\boldsymbol{\lambda})\right| + u_1u_2.$$

We now prove the result for any dimensionality $d \geq 1$ by induction on d.

- We already proved the result for the case $d = 1$.
- Assume the property holds up to a given $d \geq 1$. Let $\mathbf{u} \in \mathbb{N}^{d+1}$. Given any $\mathbf{v} \in \mathbb{Z}^{d+1}$, we denote $\mathbf{v}^{(d+1)} \in \mathbb{Z}^d$ the vector with components $v_1, \ldots, v_d$. We will make use of this

notation for several vectors in the rest of the proof. We observe that,

$$\begin{aligned}&\left\{\mathbf{s}\in\mathbb{Z}^{d+1}:g_{\mathbf{s}}(1-g_{\mathbf{s}+\mathbf{u}})=1\right\}=\\&\left\{\mathbf{s}\in\mathbb{Z}^{d+1}:g_{\mathbf{s}}=1\right\}\cap\left(\left\{\mathbf{s}\in\mathbb{Z}^{d+1}:g_{s_{d+1}+u_{d+1}}=0\right\}\cup\left\{\mathbf{s}\in\mathbb{Z}^{d+1}:g_{\mathbf{s}^{(d+1)}+\mathbf{u}^{(d+1)}}=0\right\}\right)=\\&\left(\left\{\mathbf{s}\in\mathbb{Z}^{d+1}:g_{\mathbf{s}}=1\right\}\cap\left\{\mathbf{s}\in\mathbb{Z}^{d+1}:g_{s_{d+1}+u_{d+1}}=0\right\}\right)\cup\\&\left(\left\{\mathbf{s}\in\mathbb{Z}^{d+1}:g_{\mathbf{s}}=1\right\}\cap\left\{\mathbf{s}\in\mathbb{Z}^{d+1}:g_{\mathbf{s}^{(d+1)}+\mathbf{u}^{(d+1)}}=0\right\}\right).\end{aligned}$$

Let

$$\begin{aligned}A&=\left\{\mathbf{s}\in\mathbb{Z}^{d+1}:g_{\mathbf{s}}(1-g_{\mathbf{s}+\mathbf{u}})=1\right\},\\B&=\left\{\mathbf{s}\in\mathbb{Z}^{d+1}:g_{\mathbf{s}}=1\right\}\cap\left\{\mathbf{s}\in\mathbb{Z}^{d+1}:g_{s_{d+1}+u_{d+1}}=0\right\},\\C&=\left\{\mathbf{s}\in\mathbb{Z}^{d+1}:g_{\mathbf{s}}=1\right\}\cap\left\{\mathbf{s}\in\mathbb{Z}^{d+1}:g_{\mathbf{s}^{(d+1)}+\mathbf{u}^{(d+1)}}=0\right\}.\end{aligned}$$

The idea here is that we *split* the problem between the last dimension (set B) and the d first dimensions taken altogether (set C). We then have, since $A=B\cup C$,

$$\sum_{\mathbf{s}\in A}e^{i\mathbf{s}\cdot\boldsymbol{\lambda}}=\sum_{\mathbf{s}\in B}e^{i\mathbf{s}\cdot\boldsymbol{\lambda}}+\sum_{\mathbf{s}\in C}e^{i\mathbf{s}\cdot\boldsymbol{\lambda}}-\sum_{\mathbf{s}\in B\cap C}e^{i\mathbf{s}\cdot\boldsymbol{\lambda}},$$

and by the triangle inequality,

$$\left|\sum_{\mathbf{s}\in A}e^{i\mathbf{s}\cdot\boldsymbol{\lambda}}\right|\le\left|\sum_{\mathbf{s}\in B}e^{i\mathbf{s}\cdot\boldsymbol{\lambda}}\right|+\left|\sum_{\mathbf{s}\in C}e^{i\mathbf{s}\cdot\boldsymbol{\lambda}}\right|+\left|\sum_{\mathbf{s}\in B\cap C}e^{i\mathbf{s}\cdot\boldsymbol{\lambda}}\right|.$$

We consider each term separately. Firstly,

$$\begin{aligned}\left|\sum_{\mathbf{s}\in B}e^{i\mathbf{s}\cdot\boldsymbol{\lambda}}\right|&=\left|\sum_{s_1=0}^{n_1-1}\cdots\sum_{s_d=0}^{n_d-1}\sum_{s_{d+1}=n_{d+1}-u_{d+1}}^{n_{d+1}-1}e^{i\sum_{j=1}^{d+1}s_j\lambda_j}\right|\\&=\left|\left(\sum_{s_{d+1}=n_{d+1}-u_{d+1}}^{n_{d+1}-1}e^{is_{d+1}\lambda_{d+1}}\right)\left(\sum_{s_1=0}^{n_1-1}\cdots\sum_{s_d=0}^{n_d-1}e^{i\sum_{j=1}^{d}s_j\lambda_j}\right)\right|\\&=\left|\sum_{s_{d+1}=n_{d+1}-u_{d+1}}^{n_{d+1}-1}e^{is_{d+1}\lambda_{d+1}}\right|\left|\sum_{s_1=0}^{n_1-1}\cdots\sum_{s_d=0}^{n_d-1}e^{i\sum_{j=1}^{d}s_j\lambda_j}\right|\\&\le u_{d+1}\left|\Delta_{\mathbf{n}}^{(d+1)}(\boldsymbol{\lambda})\right|.\end{aligned}$$

Secondly, using the fact that the property holds up to dimensionality d,

$$\begin{aligned}
\left|\sum_{\mathbf{s}\in C} e^{i\mathbf{s}\cdot\boldsymbol{\lambda}}\right| &= \left|\sum_{\mathbf{s}\in\mathbb{Z}^{d+1}} \mathbb{1}_C(\mathbf{s}) e^{i\mathbf{s}\cdot\boldsymbol{\lambda}}\right| \\
&= \left|\sum_{\mathbf{s}\in\mathbb{Z}^{d+1}} g_{\mathbf{s}}(1-g_{\mathbf{s}^{(d+1)}+\mathbf{u}^{(d+1)}}) e^{i\sum_{j=1}^{d+1} s_j\lambda_j}\right| \\
&= \left|\left(\sum_{s_{d+1}=0}^{n_{d+1}} e^{is_{d+1}\lambda_{d+1}}\right)\left(\sum_{\mathbf{s}\in\mathbb{Z}^{d}} g_{\mathbf{s}}(1-g_{\mathbf{s}+\mathbf{u}^{(d+1)}}) e^{i\sum_{j=1}^{d} s_j\lambda_j}\right)\right| \\
&\le \left|\sum_{s_{d+1}=0}^{n_{d+1}-1} e^{is_{d+1}\lambda_{d+1}}\right| \left(\sum_{j=1}^{d} u_j \left|\Delta_{\mathbf{n}^{(d+1)}}^{(j)}(\boldsymbol{\lambda})\right| + \sum_{\substack{j,k=1\\k>j}}^{d} u_j u_k \left|\Delta_{\mathbf{n}^{(d+1)}}^{(j,k)}(\boldsymbol{\lambda})\right| + \ldots + u_1\cdots u_d\right) \\
&= \sum_{j=1}^{d} u_j \left|\Delta_{\mathbf{n}}^{(j)}(\boldsymbol{\lambda})\right| + \sum_{\substack{j,k=1\\k>j}}^{d} u_j u_k \left|\Delta_{\mathbf{n}}^{(j,k)}(\boldsymbol{\lambda})\right| + \ldots + u_1\cdots u_d \left|\Delta_{\mathbf{n}}^{(1,\ldots,d)}(\boldsymbol{\lambda})\right|,
\end{aligned}$$

where in the last equality we used the fact that $\left|\sum_{s_{d+1}=0}^{n_{d+1}-1} e^{is_{d+1}\lambda_{d+1}}\right|\left|\Delta_{\mathbf{n}^{(d+1)}}^{(j)}(\boldsymbol{\lambda})\right| = \left|\Delta_{\mathbf{n}}^{(j)}(\boldsymbol{\lambda})\right|$. Thirdly, again using the fact that the property holds up to dimensionality d,

$$\begin{aligned}
\left|\sum_{\mathbf{s}\in B\cap C} e^{i\mathbf{s}\cdot\boldsymbol{\lambda}}\right| &\le \left|\sum_{s_{d+1}=n_{d+1}-u_{d+1}}^{n_{d+1}-1} e^{is_{d+1}\lambda_{d+1}}\right| \\
&\quad\times\left(\sum_{j=1}^{d} u_j \left|\Delta_{\mathbf{n}}^{(j)}(\boldsymbol{\lambda})\right| + \sum_{\substack{j,k=1\\k>j}}^{d} u_j u_k \left|\Delta_{\mathbf{n}^{(d+1)}}^{(j,k)}(\boldsymbol{\lambda})\right| + \ldots + u_1\cdots u_d\right) \\
&\le u_{d+1}\left(\sum_{j=1}^{d} u_j \left|\Delta_{\mathbf{n}^{(d+1)}}^{(j)}(\boldsymbol{\lambda})\right| + \sum_{\substack{j,k=1\\k>j}}^{d} u_j u_k \left|\Delta_{\mathbf{n}^{(d+1)}}^{(j,k)}(\boldsymbol{\lambda})\right| + \ldots + u_1\cdots u_d\right) \\
&= u_{d+1}\left(\sum_{j=1}^{d} u_j \left|\Delta_{\mathbf{n}}^{(j,d+1)}(\boldsymbol{\lambda})\right| + \sum_{\substack{j,k=1\\k>j}}^{d} u_j u_k \left|\Delta_{\mathbf{n}}^{(j,k,d+1)}(\boldsymbol{\lambda})\right| + \ldots + u_1\cdots u_d\right) \\
&= \sum_{j=1}^{d} u_j u_{d+1} \left|\Delta_{\mathbf{n}}^{(j,d+1)}(\boldsymbol{\lambda})\right| + \sum_{\substack{j,k=1\\k>j}}^{d} u_j u_k u_{d+1} \left|\Delta_{\mathbf{n}}^{(j,k,d+1)}(\boldsymbol{\lambda})\right| + \ldots + u_1\cdots u_{d+1}.
\end{aligned}$$

Substituting these expressions into (18), we obtain,

$$\left|\sum_{\mathbf{s}\in\mathbb{Z}^{d+1}} g_{\mathbf{s}} g_{\mathbf{s}+\mathbf{u}} e^{i\mathbf{s}\cdot\boldsymbol{\lambda}} - \Delta_{\mathbf{n}}(\boldsymbol{\lambda})\right| \le \sum_{j=1}^{d+1} u_j \left|\Delta_{\mathbf{n}}^{(j)}(\boldsymbol{\lambda})\right| + \sum_{\substack{j,k=1\\k>j}}^{d+1} u_j u_k \left|\Delta_{\mathbf{n}}^{(j,k)}(\boldsymbol{\lambda})\right| + \ldots + u_1\cdots u_{d+1},$$

which is exactly the desired property for dimensionality $d+1$.

By induction, we conclude that the property holds for any dimensionality d. □

As an example, in dimension $d = 3$, the inequality takes the following form,

$$\left|\sum_{\mathbf{s}\in\mathbb{Z}^3} g_{\mathbf{s}} g_{\mathbf{s}+\mathbf{u}} e^{i\mathbf{s}\cdot\boldsymbol{\lambda}} - \Delta_{\mathbf{n}}(\boldsymbol{\lambda})\right| \leq u_1 \left|\Delta_{\mathbf{n}}^{(1)}(\boldsymbol{\lambda})\right| + u_2 \left|\Delta_{\mathbf{n}}^{(2)}(\boldsymbol{\lambda})\right| + u_3 \left|\Delta_{\mathbf{n}}^{(3)}(\boldsymbol{\lambda})\right|$$
$$+ u_2 u_3 \left|\Delta_{\mathbf{n}}^{(2,3)}(\boldsymbol{\lambda})\right| + u_1 u_3 \left|\Delta_{\mathbf{n}}^{(1,3)}(\boldsymbol{\lambda})\right| + u_1 u_2 \left|\Delta_{\mathbf{n}}^{(1,2)}(\boldsymbol{\lambda})\right|$$
$$+ u_1 u_2 u_3.$$

We now use this result to approximate the L-th order cumulant of the multi-dimensional DFT.

Lemma E (L-th order cumulants of the DFT). Suppose Assumption 3 holds. For an integer $L \geq 2$, and $\boldsymbol{\omega}_1, \ldots, \boldsymbol{\omega}_L \in \mathbb{R}^d$, we have,

$$\operatorname{cum}_L\left\{\widetilde{J}_{\mathbf{n}}(\boldsymbol{\omega}_1), \ldots, \widetilde{J}_{\mathbf{n}}(\boldsymbol{\omega}_L)\right\} = \Delta_{\mathbf{n}}\left(\sum_{j=1}^{L} \boldsymbol{\omega}_j\right) f_L(\boldsymbol{\omega}_1, \ldots, \boldsymbol{\omega}_{L-1}) + \mathcal{O}\left(\Lambda\left(\sum_{j=1}^{L} \boldsymbol{\omega}_j\right)\right),$$

where f_L is the L-th cumulant spectral density and where we have defined,

$$\Lambda(\boldsymbol{\lambda}) = \sum_{j=1}^{d} \left|\Delta_{\mathbf{n}}^{(j)}(\boldsymbol{\lambda})\right| + \sum_{\substack{j,k=1 \\ k>j}}^{d} \left|\Delta_{\mathbf{n}}^{(j,k)}(\boldsymbol{\lambda})\right| + \ldots + 1, \tag{24}$$

and where the $\mathcal{O}(\cdot)$ does not depend on $\boldsymbol{\omega}_1, \ldots, \boldsymbol{\omega}_L$.

Proof. By properties of cumulants, see Lemma B, direct calculations give,

$$\operatorname{cum}_L\left\{\widetilde{J}_{\mathbf{n}}(\boldsymbol{\omega}_1), \ldots, \widetilde{J}_{\mathbf{n}}(\boldsymbol{\omega}_L)\right\} = \sum_{\mathbf{s}_1,\ldots,\mathbf{s}_L\in\mathbb{Z}^d} \operatorname{cum}(X_{\mathbf{s}_1}, \ldots, X_{\mathbf{s}_L}) g_{\mathbf{s}_1} \ldots g_{\mathbf{s}_L} e^{-i\sum_{j=1}^{L} \boldsymbol{\omega}_j\cdot\mathbf{s}_j}$$
$$= \sum_{\mathbf{s}_1} \sum_{\mathbf{u}_1,\ldots,\mathbf{u}_{L-1}} c_L(\mathbf{u}_1, \ldots, \mathbf{u}_{L-1}) g_{\mathbf{s}_1} g_{\mathbf{s}_1+\mathbf{u}_1} \cdots g_{\mathbf{s}_1+\mathbf{u}_{L-1}} e^{-i\sum_{j=1}^{L-1} \boldsymbol{\omega}_j\cdot\mathbf{u}_j} e^{-i\sum_{j=1}^{L} \boldsymbol{\omega}_j\cdot\mathbf{s}_1}$$
$$= \sum_{\mathbf{u}_1,\ldots,\mathbf{u}_{L-1}} c_L(\mathbf{u}_1, \ldots, \mathbf{u}_{L-1}) e^{-i\sum_{j=1}^{L-1} \boldsymbol{\omega}_j\cdot\mathbf{u}_j} \sum_{\mathbf{s}_1} g_{\mathbf{s}_1} g_{\mathbf{s}_1+\mathbf{u}_1} \cdots g_{\mathbf{s}_1+\mathbf{u}_{L-1}} e^{-i\sum_{j=1}^{L} \boldsymbol{\omega}_j\cdot\mathbf{s}_1}. \tag{25}$$

Suppose for convenience that $\mathbf{u}_1, \ldots, \mathbf{u}_{L-1}$ all have non-negative components. The general case can be treated similarly, please see our comment on this at the end of this proof. Additionally, denote $\tilde{\mathbf{u}} \in \mathbb{N}^d$ as the vector defined by

$$\tilde{u}_j = \max\{\mathbf{u}_k \cdot \mathbf{e}_j : k = 1, \ldots, L-1\}, \tag{26}$$

where $\mathbf{e}_j, j = 1, \ldots, d$ denotes the d-vector with all components set to zero except for the j-th component which is set to 1, such that $\mathbf{u}_k \cdot \mathbf{e}_j$ is the j-th component of $\mathbf{u}_k$. The right-most term of (25) can be approximated using the fact that, for $\boldsymbol{\lambda} \in \mathbb{R}^d$,

$$\left|\sum_{\mathbf{s}_1\in\mathbb{Z}^d} g_{\mathbf{s}_1} g_{\mathbf{s}_1+\mathbf{u}_1} \cdots g_{\mathbf{s}_1+\mathbf{u}_{L-1}} e^{-i\boldsymbol{\lambda}\cdot\mathbf{s}_1} - \Delta_{\mathbf{n}}(\boldsymbol{\lambda})\right| = \left|\sum_{\mathbf{s}_1} g_{\mathbf{s}_1}\left(g_{\mathbf{s}_1+\mathbf{u}_1} \cdots g_{\mathbf{s}_1+\mathbf{u}_{L-1}} - 1\right) e^{-i\boldsymbol{\lambda}\cdot\mathbf{s}_1}\right|$$
$$= \left|\sum_{\mathbf{s}_1} g_{\mathbf{s}_1}\left(g_{\mathbf{s}_1+\tilde{\mathbf{u}}} - 1\right) e^{-i\boldsymbol{\lambda}\cdot\mathbf{s}_1}\right|,$$

due to assuming that the grid is fully observed and setting $g_{\mathbf{s}} = 1$ on the grid and 0 otherwise. For instance, in the case $L = 3$, we have for $\mathbf{s}_1 \in \mathbb{Z}^d$, $g_{\mathbf{s}_1} g_{\mathbf{s}_1+\mathbf{u}_1} g_{\mathbf{s}_1+\mathbf{u}_2} = 1 \iff \mathbf{s}_1 \in \mathcal{J}_{\mathbf{n}}$ and $\mathbf{s}_1+\mathbf{u}_1 \in \mathcal{J}_{\mathbf{n}}$ and $\mathbf{s}_1 + \mathbf{u}_2 \in \mathcal{J}_{\mathbf{n}} \iff \mathbf{s}_1 \in \mathcal{J}_{\mathbf{n}}$ and $\mathbf{s}_1 + \tilde{\mathbf{u}} \in \mathcal{J}_{\mathbf{n}} \iff g_{\mathbf{s}_1} g_{\mathbf{s}_1+\tilde{\mathbf{u}}} = 1$.

According to Lemma D, we therefore have,

$$\left| \sum_{\mathbf{s}\in\mathbb{Z}^d} g_{\mathbf{s}} g_{\mathbf{s}+\tilde{\mathbf{u}}} e^{i\mathbf{s}\cdot\boldsymbol{\lambda}} - \Delta_{\mathbf{n}}(\boldsymbol{\lambda}) \right| \leq \sum_{j=1}^{d} \widetilde{u}_j \left| \Delta_{\mathbf{n}}^{(j)}(\boldsymbol{\lambda}) \right| + \sum_{\substack{j,k=1\\k>j}}^{d} \widetilde{u}_j \widetilde{u}_k \left| \Delta_{\mathbf{n}}^{(j,k)}(\boldsymbol{\lambda}) \right| + \ldots + \widetilde{u}_1 \cdots \widetilde{u}_d.$$

We use the inequality $\widetilde{u}_1 \ldots \widetilde{u}_d \leq (\max_{i=1,\ldots,d} \widetilde{u}_i)^d \leq \widetilde{u}_1^d + \ldots + \widetilde{u}_d^d$ (by definition of $\tilde{\mathbf{u}}$ its components are non-negative) and obtain,

$$\left| \sum_{\mathbf{s}\in\mathbb{Z}^d} g_{\mathbf{s}} g_{\mathbf{s}+\tilde{\mathbf{u}}} e^{i\mathbf{s}\cdot\boldsymbol{\lambda}} - \Delta_{\mathbf{n}}(\boldsymbol{\lambda}) \right| \leq \left(\widetilde{u}_1^d + \ldots + \widetilde{u}_d^d\right) \left(\sum_{j=1}^{d} \left| \Delta_{\mathbf{n}}^{(j)}(\boldsymbol{\lambda}) \right| + \sum_{\substack{j,k=1\\k>j}}^{d} \left| \Delta_{\mathbf{n}}^{(j,k)}(\boldsymbol{\lambda}) \right| + \ldots + 1 \right).$$

Now given our definition of $\tilde{\mathbf{u}} = \begin{pmatrix}\tilde{u}_1 & \ldots & \tilde{u}_d\end{pmatrix}^T$, see (26), we have $\widetilde{u}_1^d + \ldots + \widetilde{u}_d^d \leq \|\mathbf{u}_1\|_1^d + \ldots + \|\mathbf{u}_{L-1}\|_1^d$, and therefore,

$$\left| \sum_{s} g_{\mathbf{s}} g_{\mathbf{s}+\tilde{\mathbf{u}}} e^{-i\boldsymbol{\lambda}\cdot\mathbf{s}} - \Delta_n(\boldsymbol{\lambda}) \right| \leq \left(\|\mathbf{u}_1\|_1^d + \ldots + \|\mathbf{u}_{L-1}\|_1^d\right) \left(\sum_{j=1}^{d} \left| \Delta_{\mathbf{n}}^{(j)}(\boldsymbol{\lambda}) \right| + \sum_{\substack{j,k=1\\k>j}}^{d} \left| \Delta_{\mathbf{n}}^{(j,k)}(\boldsymbol{\lambda}) \right| + \ldots + 1 \right) \tag{27}$$

Finally, going back to (25), we write

$$\sum_{\mathbf{s}_1} g_{\mathbf{s}_1} g_{\mathbf{s}_1+\mathbf{u}_1} \cdots g_{\mathbf{s}_1+\mathbf{u}_{L-1}} e^{-i\sum_{j=1}^{L} \boldsymbol{\omega}_j\cdot\mathbf{s}_1} = \Delta_{\mathbf{n}} \left(\sum_{j=1}^{L} \boldsymbol{\omega}_j \cdot \mathbf{s}_1 \right) + \mathcal{E} \left(\sum_{j=1}^{L} \boldsymbol{\omega}_j \cdot \mathbf{s}_1 \right),$$

with

$$\mathcal{E}(\boldsymbol{\lambda}) = \sum_{\mathbf{s}_1} g_{\mathbf{s}_1} g_{\mathbf{s}_1+\mathbf{u}_1} \cdots g_{\mathbf{s}_1+\mathbf{u}_{L-1}} e^{-i\boldsymbol{\lambda}} - \Delta_{\mathbf{n}}(\boldsymbol{\lambda}),$$

where for simplicity we do not denote explicitly the dependence of $\mathcal{E}(\cdot)$ on $\mathbf{u}_1, \ldots, \mathbf{u}_{L-1}$. We then use the upper-bound (27) we derived for $|\mathcal{E}(\boldsymbol{\lambda})|$, and Assumption 3 on the summability of cumulants to obtain,

$$\left| \sum_{\mathbf{u}_1,\ldots,\mathbf{u}_{L-1}} c_L(\mathbf{u}_1, \ldots, \mathbf{u}_{L-1}) e^{-i\boldsymbol{\lambda}\cdot\mathbf{u}_j} \mathcal{E}(\boldsymbol{\lambda}) \right| = \mathcal{O} \left(\sum_{j=1}^{d} \left| \Delta_{\mathbf{n}}^{(j)}(\boldsymbol{\lambda}) \right| + \sum_{\substack{j,k=1\\k>j}}^{d} \left| \Delta_{\mathbf{n}}^{(j,k)}(\boldsymbol{\lambda}) \right| + \ldots + 1 \right).$$

This concludes the proof. We now comment on how to adapt the proof to the case where $\mathbf{u}_1, \ldots, \mathbf{u}_{L-1}$ are not restricted to having non-negative components. This is achieved by replacing (26) with,

$$\begin{aligned}\tilde{u}_j^+ &= \max\{0, \max\{\mathbf{u}_k \cdot \mathbf{e}_j : k = 1, \ldots, L-1\}\}\\ \tilde{u}_j^- &= \max\{0, \max\{-\mathbf{u}_k \cdot \mathbf{e}_j : k = 1, \ldots, L-1\}\}.\end{aligned}$$

This is because when allowing for negative components, we have to treat both boundaries of the domain along each dimension $j = 1, \cdots, d$. This is accounted for in the final formula in the $\mathcal{O}(\cdot)$.

□

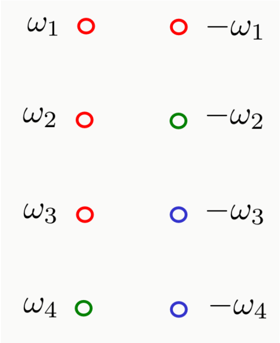

Fig. 7. Example of an indecomposable partition of a 4×2 table that is used in expressing the 4-th order cumulants of the periodogram at frequencies $\boldsymbol{\omega}_1, \boldsymbol{\omega}_2, \boldsymbol{\omega}_3, \boldsymbol{\omega}_4$ in terms of cumulants of the DFT at frequencies $\boldsymbol{\omega}_1, -\boldsymbol{\omega}_1, \boldsymbol{\omega}_2, -\boldsymbol{\omega}_2, \boldsymbol{\omega}_3, -\boldsymbol{\omega}_3, \boldsymbol{\omega}_4, -\boldsymbol{\omega}_4$. The chosen indecomposable partition has 3 sets, indicated by the colors red, green and blue.

In the proof of Proposition A of this Supplementary Material, when expressing the cumulant of order L of the periodogram evaluated at Fourier frequencies $\boldsymbol{\omega}_1 \ldots, \boldsymbol{\omega}_L \in \Omega_{\mathbf{n}}$ in terms of cumulants of the DFT (which we studied in Lemma E of this Supplementary Material), we will need to understand the order of terms of the form

$$\sum_{\boldsymbol{\omega}_1, \ldots, \boldsymbol{\omega}_L \in \Omega_{\mathbf{n}}} \prod_{\nu_r \in \nu} \Delta_{\mathbf{n}} \left(\sum_{j \in \nu_r} \boldsymbol{\omega}_j \right), \tag{28}$$

where ν is an indecomposable partition of the $L \times 2$ table given in (22) and where we set $\boldsymbol{\omega}_{k+L} = -\boldsymbol{\omega}_k, \quad k = 1, \ldots, L$ (see Figure 7 and compare to the $L \times 2$ table (22)). While the $\Delta_{\mathbf{n}}(\cdot)$ function can take value $|\mathbf{n}|$, this only occurs under linear constraints on the $\boldsymbol{\omega}_1, \ldots, \boldsymbol{\omega}_L$. For example, in the case $L = 4$ and for the partition of the $L \times 2$ table represented in Figure 7, we get the following set of linear constraints on the Fourier frequencies,

$$\begin{cases} \boldsymbol{\omega}_1 - \boldsymbol{\omega}_1 + \boldsymbol{\omega}_2 + \boldsymbol{\omega}_3 \equiv 0 \ [2\pi] \\ \boldsymbol{\omega}_4 - \boldsymbol{\omega}_2 \equiv 0 \ [2\pi] \\ -\boldsymbol{\omega}_4 - \boldsymbol{\omega}_3 \equiv 0 \ [2\pi] \end{cases}, \tag{29}$$

two of which are linearly independent. The following lemma makes this property explicit.

Lemma F. Let $\nu = (\nu_1, \ldots, \nu_p)$ be an indecomposable partition of the $L \times 2$ table, where L is a positive integer. The following system of linear equations in $(\boldsymbol{\omega}_1, \ldots, \boldsymbol{\omega}_L)$

$$(\mathcal{S}_\nu) \begin{cases} \sum_{j \in \nu_1} \boldsymbol{\omega}_j & \equiv 0 \ [2\pi] \\ & \vdots \\ \sum_{j \in \nu_p} \boldsymbol{\omega}_j & \equiv 0 \ [2\pi] \end{cases} \tag{30}$$

imposes $p - 1$ linear constraints on the $(\boldsymbol{\omega}_1, \ldots, \boldsymbol{\omega}_L)$.

Proof. We remind the reader that we have defined $\boldsymbol{\omega}_{k+L} \equiv -\boldsymbol{\omega}_k$ for $k = 1, \ldots, L$. The proof is done by induction on the number of sets p in the partition.

- In the case $p = 1$, the partition consists of a unique set, and each $\boldsymbol{\omega}_j$ in the summation is cancelled out by $\boldsymbol{\omega}_{L+j}$. Hence $\sum_{j\in\nu_1} \boldsymbol{\omega}_j \equiv 0 \ [2\pi] \iff 0 \equiv 0 \ [2\pi]$, so that there are no linear constraints and the property holds for $p = 1$.

- Suppose the property holds up to a given positive integer p. We want to show that it also holds for any partition with $p + 1$ sets. Therefore, let $\nu = (\nu_1, \dots, \nu_{p+1})$ be an indecomposable partition of the $L\times 2$ table with cardinality $p+1$. Without loss of generality, we assume that the ordering of the sets is such that ν_1 communicates with ν_2, i.e. there exists $k \in \{1, \dots, L\}$ such that $k \in \nu_1$ and $L + k \in \nu_2$ (or the reverse case, but again we can treat either of these two cases without loss of generality), i.e. the k-th row has one element that belongs to ν_1 and the other one to ν_2, since in the $L \times 2$ table k and $L + k$ are on the same row.

  We then observe that we can rewrite the system

$$(\mathcal{S}_\nu) \begin{cases} \sum_{j\in\nu_1} \boldsymbol{\omega}_j & \equiv 0 \ [2\pi] \\ & \vdots \\ \sum_{j\in\nu_p+1} \boldsymbol{\omega}_j & \equiv 0 \ [2\pi] \end{cases}, \tag{31}$$

  as

$$(\mathcal{S}_\nu) \begin{cases} \sum_{j\in\nu_1} \boldsymbol{\omega}_j & \equiv 0 \ [2\pi] \\ \sum_{j\in\nu_1\cup\nu_2} \boldsymbol{\omega}_j & \equiv 0 \ [2\pi] \\ \sum_{j\in\nu_3} \boldsymbol{\omega}_j & \equiv 0 \ [2\pi] \\ & \vdots \\ \sum_{j\in\nu_p} \boldsymbol{\omega}_j & \equiv 0 \ [2\pi] \end{cases}, \tag{32}$$

  where the second equation in (32) is obtained by summing the first two equations in (31), using the fact that $\nu_1 \cap \nu_2 = 0$, by definition of a partition. Based on this partition ν, we define a new partition $\widetilde{\nu} = (\nu_1 \cup \nu_2, \nu_3, \dots, \nu_{p+1})$. The set $\widetilde{\nu}$ is clearly a partition of the $L \times 2$ table, and it has p sets. Additionally, one can verify that this new partition $\widetilde{\nu}$ is also indecomposable. The solution space to $\mathcal{S}_\nu$ is therefore the intersection between the solution spaces to $\mathcal{S}_{\widetilde{\nu}}$ and $\sum_{j\in\nu_1} \boldsymbol{\omega}_j \equiv 0 \ [2\pi]$.

  By assumption, $\mathcal{S}_{\widetilde{\nu}}$ enforces $p-1$ linear constraints on $(\boldsymbol{\omega}_1, \dots, \boldsymbol{\omega}_L)$. It therefore suffices to show that $\sum_{j\in\nu_1} \boldsymbol{\omega}_j \equiv 0 \ [2\pi]$ and the system $\mathcal{S}_{\widetilde{\nu}}$ are linearly independent. Or, equivalently, that there exists a set of values of $(\boldsymbol{\omega}_1, \dots, \boldsymbol{\omega}_L)$ that is a solution of $\mathcal{S}_{\widetilde{\nu}}$ but such that $\sum_{j\in\nu_1} \boldsymbol{\omega}_j \not\equiv 0 \ [2\pi]$. Such a set of values is obtained by setting all components equal to zero modulo 2π, except for the k-th and $L + k$-th components, where k was defined earlier in this proof as the row on which the sets ν_1 and ν_2 communicate. More precisely we set $\boldsymbol{\omega}_k = a$ and $\boldsymbol{\omega}_{L+k} = -a$ where a is chosen such that $a \not\equiv 0 \ [2\pi]$. Hence the number of linear constraints enforced by $\mathcal{S}_\nu$ on $(\boldsymbol{\omega}_1, \dots, \boldsymbol{\omega}_L)$ is $p - 1 + 1 = (p + 1) - 1$, so that the property also holds for any partition with $p + 1$ sets.

By induction, since we proved the result for the partition of cardinality $p = 1$, we can conclude that the property holds for any indecomposable partition. □

We can now proceed to determine an upper-bound for the higher-order cumulants of linear functionals of the periodogram, following the proof of Brillinger (2001, Theorem 5.10.1).

Proposition A. Let L be a positive integer. We have,

$$\mathrm{cum}_L\left\{|\mathbf{n}|^{-1}\sum_{\boldsymbol{\omega}\in\Omega_{\mathbf{n}}} w(\boldsymbol{\omega})I_{\mathbf{n}}(\boldsymbol{\omega})\right\} = \mathcal{O}\left(|\mathbf{n}|^{1-L}\right). \tag{33}$$

Proof. Using the properties of cumulants given in Lemma B from this Supplementary Material, we have

$$\mathrm{cum}_L\left\{|\mathbf{n}|^{-1}\sum_{\boldsymbol{\omega}\in\Omega_{\mathbf{n}}} w(\boldsymbol{\omega})I_{\mathbf{n}}(\boldsymbol{\omega})\right\} = \\ |\mathbf{n}_k|^{-L}\sum_{\boldsymbol{\omega}_1,\dots,\boldsymbol{\omega}_L\in\Omega_{\mathbf{n}_k}} w_k(\boldsymbol{\omega}_1)\dots w_k(\boldsymbol{\omega}_L)\mathrm{cum}\left[I_{\mathbf{n}_k}(\boldsymbol{\omega}_1),\dots,I_{\mathbf{n}_k}(\boldsymbol{\omega}_L)\right].$$

According to Lemma C of this Supplementary material, we obtain,

$$\begin{aligned}\mathrm{cum}\left[I_{\mathbf{n}_k}(\boldsymbol{\omega}_1),\dots,I_{\mathbf{n}_k}(\boldsymbol{\omega}_L)\right] &= \mathrm{cum}\left[J_{\mathbf{n}_k}(\boldsymbol{\omega}_1)J_{\mathbf{n}_k}(-\boldsymbol{\omega}_1),\dots,J_{\mathbf{n}_k}(\boldsymbol{\omega}_L)J_{\mathbf{n}_k}(-\boldsymbol{\omega}_L)\right]\\ &= \sum_{\nu}\mathrm{cum}\left[J_{\mathbf{n}_k}(\boldsymbol{\omega}_j):j\in\nu_1\right]\dots\mathrm{cum}\left[J_{\mathbf{n}_k}(\boldsymbol{\omega}_j):j\in\nu_p\right],\end{aligned} \tag{34}$$

where the summation is over indecomposable partitions $\nu=(\nu_1,\dots,\nu_p)$ of the $L\times 2$ table (22), and where we define $\boldsymbol{\omega}_{j+L}\equiv-\boldsymbol{\omega}_j, j=1,\dots,L$. Hence, reminding the reader that we write $\widetilde{J}_{\mathbf{n}}(\boldsymbol{\omega})=|\mathbf{n}|^{\frac{1}{2}}J_{\mathbf{n}}(\boldsymbol{\omega})$,

$$\begin{aligned}&\mathrm{cum}_L\left\{|\mathbf{n}|^{-1}\sum_{\boldsymbol{\omega}\in\Omega_{\mathbf{n}}} w(\boldsymbol{\omega})I_{\mathbf{n}}(\boldsymbol{\omega})\right\} =\\ &|\mathbf{n}_k|^{-L}\sum_{\boldsymbol{\omega}_1,\dots,\boldsymbol{\omega}_L\in\Omega_{\mathbf{n}_k}} w_k(\boldsymbol{\omega}_1)\dots w_k(\boldsymbol{\omega}_L)\sum_{\nu}\mathrm{cum}\left[J_{\mathbf{n}_k}(\boldsymbol{\omega}_j):j\in\nu_1\right]\dots\mathrm{cum}\left[J_{\mathbf{n}_k}(\boldsymbol{\omega}_j):j\in\nu_p\right]\\ &= |\mathbf{n}_k|^{-2L}\sum_{\boldsymbol{\omega}_1,\dots,\boldsymbol{\omega}_L\in\Omega_{\mathbf{n}_k}} w_k(\boldsymbol{\omega}_1)\dots w_k(\boldsymbol{\omega}_L)\sum_{\nu}\mathrm{cum}\left[\widetilde{J}_{\mathbf{n}_k}(\boldsymbol{\omega}_j):j\in\nu_1\right]\dots\mathrm{cum}\left[\widetilde{J}_{\mathbf{n}_k}(\boldsymbol{\omega}_j):j\in\nu_p\right].\\ &= |\mathbf{n}_k|^{-2L}\sum_{\nu}\sum_{\boldsymbol{\omega}_1,\dots,\boldsymbol{\omega}_L\in\Omega_{\mathbf{n}_k}} w_k(\boldsymbol{\omega}_1)\dots w_k(\boldsymbol{\omega}_L)\prod_{r=1}^{p}\mathrm{cum}\left[\widetilde{J}_{\mathbf{n}_k}(\boldsymbol{\omega}_j):j\in\nu_p\right].\end{aligned} \tag{35}$$

We now make use of Lemma E in which we obtained an expression for the terms

$$\mathrm{cum}\left[\widetilde{J}_{\mathbf{n}_k}(\boldsymbol{\omega}_j):j\in\nu_p\right],\quad r=1,\dots,p,$$

which appear in the product in (35). This leads us to,

$$\begin{aligned}&\mathrm{cum}_L\left\{|\mathbf{n}|^{-1}\sum_{\boldsymbol{\omega}\in\Omega_{\mathbf{n}}} w(\boldsymbol{\omega})I_{\mathbf{n}}(\boldsymbol{\omega})\right\} =\\ &|\mathbf{n}_k|^{-2L}\sum_{\nu}\sum_{\boldsymbol{\omega}_1,\dots,\boldsymbol{\omega}_L\in\Omega_{\mathbf{n}_k}} w_k(\boldsymbol{\omega}_1)\dots w_k(\boldsymbol{\omega}_L)\\ &\times\prod_{r=1}^{p}\left\{f_{m_r+1}(\boldsymbol{\omega}_l:l\in\nu_r)\Delta_{\mathbf{n}}\left(\sum_{l\in\nu_r}\boldsymbol{\omega}_j\right)+\mathcal{O}\left(\sum_{j=1}^{d}\left|\Delta_{\mathbf{n}}^{(j)}\left(\sum_{l\in\nu_r}\boldsymbol{\omega}_j\right)\right|+\sum_{\substack{j,k=1\\k>j}}^{d}\left|\Delta_{\mathbf{n}}^{(j,k)}\left(\sum_{l\in\nu_r}\boldsymbol{\omega}_j\right)\right|+\dots+1\right)\right\},\end{aligned}$$

where p is the cardinality of the partition ν, and for each set ν_r, $r = 1, \cdots, p$, of the partition, m_r is the the cardinality of the set ν_r. Additionally, $f_k(\cdots)$ is the k-th order cumulant spectral density. Note that the slight abuse of notation $f_{m_r+1}(\boldsymbol{\omega}_l : l \in \nu_r)$ makes sense since the cumulant spectral densities are symmetric, due to the symmetry of the cumulants themselves.

Now to determine the order of this term for a given indecomposable partition ν, we introduce some additional notation, and follow the reasoning found in Brillinger (2001) for the analysis of time series. For $r = 1, \ldots, p$, let $q_r \in \{0, \ldots, d\}$ and $l^{(r)} = l_1^{(r)}, \ldots, l_{q_r}^{(r)} \in \{1, \ldots, d\}$. Expanding the previous expression for that given partition ν will lead to a sum of terms of the form,

$$\left\{\prod_{j=1}^{d} n_j^{-2L}\right\} \sum_{\boldsymbol{\omega}_1,\ldots,\boldsymbol{\omega}_L \in \Omega_{\mathbf{n}}} \left\{\prod_{r=1}^{p} \left| \Delta_{\mathbf{n}}^{(l_1^{(r)},\ldots,l_{q_r}^{(r)})} \left(\sum_{j\in\nu_r} \boldsymbol{\omega}_j\right)\right|\right\}, \tag{36}$$

ignoring multiplicative constants and the $w_k(\cdot)$ terms for simplicity, as the latter are upper-bounded in absolute value by assumption.

Now for a given $r = 1, \ldots, p$, $\Delta_{\mathbf{n}}^{(l_1^{(r)},\ldots,l_{q_r}^{(r)})} \left(\sum_{j\in\nu_r} \boldsymbol{\omega}_j\right)$ will be zero (since the $\boldsymbol{\omega}_j$'s are Fourier frequencies) unless $\sum_{j\in\nu_r} \omega_{j,k} \equiv 0\ [2\pi]$, $\forall k \in \overline{l^{(r)}}$, where $\overline{l^{(r)}}$ denotes the complementary of $l^{(r)}$ within the set $\{1, \ldots, d\}$. In the latter case, $\Delta_{\mathbf{n}}^{(l_1^{(r)},\ldots,l_{q_r}^{(r)})} \left(\sum_{j\in\nu_r} \boldsymbol{\omega}_j\right)$ will take value $\prod_{j\in\overline{l^{(r)}}} n_j$. For each dimension $j = 1, \ldots, d$, denote $\mathcal{S}_j$ the system of linear equations expressing the constraints on the j-th dimension between $(\boldsymbol{\omega}_1, \ldots, \boldsymbol{\omega}_L)$ due to $\prod_{r=1}^{p} \Delta_{\mathbf{n}}^{(l_1^{(r)},\ldots,l_{q_r}^{(r)})} \left(\sum_{j\in\nu_r} \boldsymbol{\omega}_j\right)$. We also define $\kappa_j = \sum_{r=1}^{p} \mathbb{1}_{j\in\overline{l^{(r)}}}$ for each dimension $j = 1, \cdots, d$, and note that $\mathcal{S}_j$ is a system of κ_j linear equations, $0 \le \kappa_j \le p$. Then (36) becomes,

$$\begin{aligned} &\left\{\prod_{j=1}^{d} n_j^{-2L}\right\} \sum_{\boldsymbol{\omega}_1,\ldots,\boldsymbol{\omega}_L \in \Omega_{\mathbf{n}}} \left\{\prod_{j=1}^{d} n_j^{\kappa_j}\right\} \left\{\prod_{j=1}^{d} \mathbb{1}_{(\omega_{1,j},\ldots,\omega_{L,j})\in\mathcal{S}_j}\right\} \\ &= \left\{\prod_{j=1}^{d} n_j^{-2L} n_j^{\kappa_j}\right\} \sum_{\boldsymbol{\omega}_1,\ldots,\boldsymbol{\omega}_L \in \Omega_{\mathbf{n}}} \prod_{j=1}^{d} \mathbb{1}_{(\omega_{1,j},\ldots,\omega_{L,j})\in\mathcal{S}_j} \end{aligned} \tag{37}$$

where we make a slight abuse of notation by confounding $\mathcal{S}_j$ and its solution set. Finally, (37) becomes, with $\#\mathcal{S}_j$ the cardinality of $S_j \cap \Omega_{n_j}$,

$$\prod_{j=1}^{d} n_j^{-2L} n_j^{\kappa_j} \#\mathcal{S}_j.$$

However, we have $\#\mathcal{S}_j \le n_j^{L-\kappa_j+1}$, by generalization of Lemma F of this Supplementary Material, according to which $\mathcal{S}_j$ imposes at least $\kappa_j - 1$ independent constraints. Thus the term of interest is at most of order

$$\prod_{j=1}^{d} n_j^{-2L} n_j^{\kappa_j} n_j^{L-\kappa_j+1} = \prod_{j=1}^{d} n_j^{1-L} = \left(\prod_{j=1}^{d} n_j\right)^{1-L} = |\mathbf{n}|^{1-L},$$

which concludes the proof. □

Proof of Proposition 3

Proof. (a) *Asymptotic normality.* We first consider the case of a grid growing to infinity in all directions, i.e. $\Omega_{\mathbf{n}} = \Omega_{\mathbf{n}}^{(1)}$. Under the considered set of assumptions, i.e. Assumption 3, the variance of

$$|\mathbf{n}|^{-1} \sum_{\boldsymbol{\omega} \in \Omega_{\mathbf{n}}} w_k(\boldsymbol{\omega}) I_{\mathbf{n}}(\boldsymbol{\omega}),$$

is $\Theta\left(|\mathbf{n}|^{-1}\right)$. In order to establish asymptotic normality we therefore wish to show that the rescaled quantity $|\mathbf{n}|^{-1/2} \sum_{\boldsymbol{\omega} \in \Omega_{\mathbf{n}}} w_k(\boldsymbol{\omega}) I_{\mathbf{n}}(\boldsymbol{\omega})$ has cumulants of order 3 or greater that all converge to zero. According to Proposition A, the L-th order cumulant of $|\mathbf{n}|^{-1/2} \sum_{\boldsymbol{\omega} \in \Omega_{\mathbf{n}}} w_k(\boldsymbol{\omega}) I_{\mathbf{n}}(\boldsymbol{\omega})$ is $\mathcal{O}\left(|\mathbf{n}|^{-\frac{L}{2}+1}\right)$, which indeed converges to zero for $L \geq 3$. Thus we conclude that $|\mathbf{n}|^{-1} \sum_{\boldsymbol{\omega} \in \Omega_{\mathbf{n}}} w_k(\boldsymbol{\omega}) I_{\mathbf{n}}(\boldsymbol{\omega})$ is asymptotically normally distributed. The proof readily extends to vector-valued functions $\mathbf{w}_k(\cdot)$. In the case where one or more dimensions of the domain are bounded, $\Omega_{\mathbf{n}} = \Omega_{\mathbf{n}}^{(2)}$, and we prove the result by splitting the summation into the summation over $\Omega_{\mathbf{n}}^{(1)}$ and $\Omega_{\mathbf{n}}^{(2)} \setminus \Omega_{\mathbf{n}}^{(1)}$. Each term is treated as above, and we obtain a sum of two asymptotically normal random variables.

(b) *Asymptotic form of the variance.* For this part, it is assumed that the grid grows to infinity in all directions, which is a constraint on the observation domain. We remind the reader that in that case we choose $\Omega_{\mathbf{n}} = \Omega_{\mathbf{n}}^{(1)}$. We treat the case of scalar-valued $w_k(\cdot)$, but again the proof readily extends to vector-valued functions. We have,

$$\begin{aligned}
\operatorname{var}\left\{\frac{1}{|\mathbf{n}|} \sum_{\boldsymbol{\omega} \in \Omega_{\mathbf{n}}} w_k(\boldsymbol{\omega}) I_{\mathbf{n}}(\boldsymbol{\omega})\right\} &= \frac{1}{|\mathbf{n}|^2} \sum_{\boldsymbol{\omega}_1, \boldsymbol{\omega}_2 \in \Omega_{\mathbf{n}}} w_k(\boldsymbol{\omega}_1) w_k(\boldsymbol{\omega}_2) \operatorname{cov}\left\{I_{\mathbf{n}}(\boldsymbol{\omega}_1), I_{\mathbf{n}}(\boldsymbol{\omega}_2)\right\} \\
&= \frac{1}{|\mathbf{n}|^2} \sum_{\boldsymbol{\omega}_1, \boldsymbol{\omega}_2 \in \Omega_{\mathbf{n}}} w_k(\boldsymbol{\omega}_1) w_k(\boldsymbol{\omega}_2) \operatorname{cov}\left\{J_{\mathbf{n}}(\boldsymbol{\omega}_1) J_{\mathbf{n}}(-\boldsymbol{\omega}_1), J_{\mathbf{n}}(\boldsymbol{\omega}_2) J_{\mathbf{n}}(-\boldsymbol{\omega}_2)\right\} \\
&= \frac{1}{|\mathbf{n}|^4} \sum_{\boldsymbol{\omega}_1, \boldsymbol{\omega}_2 \in \Omega_{\mathbf{n}}} w_k(\boldsymbol{\omega}_1) w_k(\boldsymbol{\omega}_2) \operatorname{cov}\left\{\widetilde{J}_{\mathbf{n}}(\boldsymbol{\omega}_1) \widetilde{J}_{\mathbf{n}}(-\boldsymbol{\omega}_1), \widetilde{J}_{\mathbf{n}}(\boldsymbol{\omega}_2) \widetilde{J}_{\mathbf{n}}(-\boldsymbol{\omega}_2)\right\},
\end{aligned}$$

where we remind the reader that we defined $\widetilde{J}_{\mathbf{n}}(\boldsymbol{\omega}) = |\mathbf{n}|^{\frac{1}{2}} J_{\mathbf{n}}(\boldsymbol{\omega})$. Making use of Lemma C from this Supplementary Material, we have,

$$\begin{aligned}
\operatorname{cov}\left\{\widetilde{J}_{\mathbf{n}}(\boldsymbol{\omega}_1) \widetilde{J}_{\mathbf{n}}(-\boldsymbol{\omega}_1), \widetilde{J}_{\mathbf{n}}(\boldsymbol{\omega}_2) \widetilde{J}_{\mathbf{n}}(-\boldsymbol{\omega}_2)\right\} =& \operatorname{cov}\left\{\widetilde{J}_{\mathbf{n}}(\boldsymbol{\omega}_1), \widetilde{J}_{\mathbf{n}}(\boldsymbol{\omega}_2)\right\} \operatorname{cov}\left\{\widetilde{J}_{\mathbf{n}}(-\boldsymbol{\omega}_1), \widetilde{J}_{\mathbf{n}}(-\boldsymbol{\omega}_2)\right\} \\
&+ \operatorname{cov}\left\{\widetilde{J}_{\mathbf{n}}(\boldsymbol{\omega}_1), \widetilde{J}_{\mathbf{n}}(-\boldsymbol{\omega}_2)\right\} \operatorname{cov}\left\{\widetilde{J}_{\mathbf{n}}(-\boldsymbol{\omega}_1), \widetilde{J}_{\mathbf{n}}(\boldsymbol{\omega}_2)\right\} \\
&+ \operatorname{cum}_4\{\widetilde{J}_{\mathbf{n}}(\boldsymbol{\omega}_1), \widetilde{J}_{\mathbf{n}}(\boldsymbol{\omega}_2), \widetilde{J}_{\mathbf{n}}(-\boldsymbol{\omega}_1), \widetilde{J}_{\mathbf{n}}(-\boldsymbol{\omega}_2)\},
\end{aligned} \tag{38}$$

the remaining terms being zero since $\mathrm{E}\{\widetilde{J}_{\mathbf{n}}(\boldsymbol{\omega}_1)\} = \mathrm{E}\{\widetilde{J}_{\mathbf{n}}(\boldsymbol{\omega}_2)\} = 0$ as the random field is zero-mean. With Lemma E ,

$$\operatorname{cov}\left\{\widetilde{J}_{\mathbf{n}}(\boldsymbol{\omega}_1), \widetilde{J}_{\mathbf{n}}(\boldsymbol{\omega}_2)\right\} = \operatorname{cov}\left\{\widetilde{J}_{\mathbf{n}}(-\boldsymbol{\omega}_1), \widetilde{J}_{\mathbf{n}}(-\boldsymbol{\omega}_2)\right\} =$$

$$f_{X,\delta}(\boldsymbol{\omega}_1) \Delta_{\mathbf{n}}(\boldsymbol{\omega}_1 + \boldsymbol{\omega}_2) + \mathcal{O}\left(\sum_{j=1}^{d} \left|\Delta_{\mathbf{n}}^{(j)}(\boldsymbol{\omega}_1 + \boldsymbol{\omega}_2)\right| + \sum_{\substack{j,k=1 \\ k>j}}^{d} \left|\Delta_{\mathbf{n}}^{(j,k)}(\boldsymbol{\omega}_1 + \boldsymbol{\omega}_2)\right| + \ldots + 1\right),$$

as well as,

$$\operatorname{cov}\left\{\widetilde{J}_{\mathbf{n}}(\boldsymbol{\omega}_1), \widetilde{J}_{\mathbf{n}}(-\boldsymbol{\omega}_2)\right\} = \operatorname{cov}\left\{\widetilde{J}_{\mathbf{n}}(-\boldsymbol{\omega}_1), \widetilde{J}_{\mathbf{n}}(\boldsymbol{\omega}_2)\right\} =$$

$$f_{X,\delta}(\boldsymbol{\omega}_1)\Delta_{\mathbf{n}}\left(\boldsymbol{\omega}_1 - \boldsymbol{\omega}_2\right) + \mathcal{O}\left(\sum_{j=1}^{d}\left|\Delta_{\mathbf{n}}^{(j)}(\boldsymbol{\omega}_1 - \boldsymbol{\omega}_2)\right| + \sum_{\substack{j,k=1\\k>j}}^{d}\left|\Delta_{\mathbf{n}}^{(j,k)}(\boldsymbol{\omega}_1 - \boldsymbol{\omega}_2)\right| + \ldots + 1\right),$$

and,

$$\operatorname{cum}_4\{\widetilde{J}_{\mathbf{n}}(\boldsymbol{\omega}_1),\widetilde{J}_{\mathbf{n}}(\boldsymbol{\omega}_2), \widetilde{J}_{\mathbf{n}}(-\boldsymbol{\omega}_1), \widetilde{J}_{\mathbf{n}}(-\boldsymbol{\omega}_2)\}$$

$$= f_4(\boldsymbol{\omega}_1, \boldsymbol{\omega}2, -\boldsymbol{\omega}_1)\Delta_{\mathbf{n}}\left(\mathbf{0}\right) + \mathcal{O}\left(\sum_{j=1}^{d}\left|\Delta_{\mathbf{n}}^{(j)}(\mathbf{0})\right| + \sum_{\substack{j,k=1\\k>j}}^{d}\left|\Delta_{\mathbf{n}}^{(j,k)}(\mathbf{0})\right| + \ldots + 1\right).$$

With the assumption of a grid that grows to infinity in all directions, one can verify that the contribution of any term involving $\Delta_{\mathbf{n}}^{(j)}$, $\Delta_{\mathbf{n}}^{(j,k)}$ and so on, will become negligible w.r.t that of the terms involving $\Delta_{\mathbf{n}}$. We therefore limit our study to the latter terms that appear in (38).

(i) We have, reminding the reader that the function $w(\cdot)$ defined on $\mathcal{T}^d$ is extended to $\mathbb{R}^d$ by 2π-periodic extension,

$$\begin{aligned}
&\frac{1}{|\mathbf{n}|^4}\sum_{\boldsymbol{\omega}_1,\boldsymbol{\omega}_2\in\Omega_{\mathbf{n}}} w_k(\boldsymbol{\omega}_1)w_k(\boldsymbol{\omega}_2)\left[f_{X,\delta}(\boldsymbol{\omega}_1)\Delta_{\mathbf{n}}\left(\boldsymbol{\omega}_1 + \boldsymbol{\omega}_2\right)\right]\left[f_{X,\delta}(\boldsymbol{\omega}_1)\Delta_{\mathbf{n}}\left(-\boldsymbol{\omega}_1 - \boldsymbol{\omega}_2\right)\right]\\
&= \frac{1}{|\mathbf{n}|^4}\sum_{\boldsymbol{\omega}_1,\boldsymbol{\omega}_2\in\Omega_{\mathbf{n}}} w_k(\boldsymbol{\omega}_1)w_k(\boldsymbol{\omega}_2)\left(f_{X,\delta}(\boldsymbol{\omega}_1)\Delta_{\mathbf{n}}\left(\boldsymbol{\omega}_1 + \boldsymbol{\omega}_2\right)\right)^2\\
&= \frac{1}{|\mathbf{n}|^4}\sum_{\boldsymbol{\omega}_1\in\Omega_{\mathbf{n}}} w_k(\boldsymbol{\omega}_1)w_k(2\pi - \boldsymbol{\omega}_1)f_{X,\delta}(\boldsymbol{\omega}_1)^2|\mathbf{n}|^2\\
&= \frac{1}{|\mathbf{n}|^2}\sum_{\boldsymbol{\omega}_1\in\Omega_{\mathbf{n}}} w_k(\boldsymbol{\omega}_1)w_k(-\boldsymbol{\omega}_1)f_{X,\delta}(\boldsymbol{\omega}_1)^2,
\end{aligned}$$

which is asymptotically equivalent to $\frac{(2\pi)^d}{|\mathbf{n}|}\int_{\mathcal{T}^d} w(\boldsymbol{\omega})w(-\boldsymbol{\omega})f_{X,\delta}(\boldsymbol{\omega})^2 d\boldsymbol{\omega}$ by application of the Dominated Convergence Theorem.

(ii) We have,

$$\begin{aligned}
&\frac{1}{|\mathbf{n}|^4}\sum_{\boldsymbol{\omega}_1,\boldsymbol{\omega}_2\in\Omega_{\mathbf{n}}} w_k(\boldsymbol{\omega}_1)w_k(\boldsymbol{\omega}_2)\left[f_{X,\delta}(\boldsymbol{\omega}_1)\Delta_{\mathbf{n}}\left(\boldsymbol{\omega}_1 - \boldsymbol{\omega}_2\right)\right]\left[f_{X,\delta}(\boldsymbol{\omega}_1)\Delta_{\mathbf{n}}\left(-\boldsymbol{\omega}_1 + \boldsymbol{\omega}_2\right)\right]\\
&= \frac{1}{|\mathbf{n}|^4}\sum_{\boldsymbol{\omega}_1,\boldsymbol{\omega}_2\in\Omega_{\mathbf{n}}} w_k(\boldsymbol{\omega}_1)w_k(\boldsymbol{\omega}_2)\left(f_{X,\delta}(\boldsymbol{\omega}_1)\Delta_{\mathbf{n}}\left(\boldsymbol{\omega}_1 - \boldsymbol{\omega}_2\right)\right)^2\\
&= \frac{1}{|\mathbf{n}|^4}\sum_{\boldsymbol{\omega}_1\in\Omega_{\mathbf{n}}} w_k(\boldsymbol{\omega}_1)^2 f_{X,\delta}(\boldsymbol{\omega}_1)^2|\mathbf{n}|^2\\
&= \frac{1}{|\mathbf{n}|^2}\sum_{\boldsymbol{\omega}_1\in\Omega_{\mathbf{n}}} w_k(\boldsymbol{\omega}_1)^2 f_{X,\delta}(\boldsymbol{\omega}_1)^2,
\end{aligned}$$

which is asymptotically equivalent to $\frac{(2\pi)^d}{|\mathbf{n}|}\int_{\mathcal{T}^d} w(\boldsymbol{\omega})^2 f_{X,\delta}(\boldsymbol{\omega})^2 d\boldsymbol{\omega}$ again by application of the Dominated Convergence Theorem.

(iii) As for the third term,

$$\frac{1}{|\mathbf{n}|^4}\sum_{\boldsymbol{\omega}_1,\boldsymbol{\omega}_2\in\Omega_{\mathbf{n}}} w_k(\boldsymbol{\omega}_1)w_k(\boldsymbol{\omega}_2)f_4(\boldsymbol{\omega}_1,\boldsymbol{\omega}_2,-\boldsymbol{\omega}_1)\Delta_{\mathbf{n}}(\mathbf{0})$$

is asymptotically equivalent to

$$\frac{(2\pi)^d}{|\mathbf{n}|}\int_{\mathcal{T}^d}\int_{\mathcal{T}^d} w(\boldsymbol{\omega}_1)w(\boldsymbol{\omega}_2)f_{X,4,\delta}(\boldsymbol{\omega}_1,\boldsymbol{\omega}_2,-\boldsymbol{\omega}_1)d\boldsymbol{\omega}_1 d\boldsymbol{\omega}_2,$$

again by application of the Dominated Convergence Theorem, and having noted that $\Delta_{\mathbf{n}}(\mathbf{0}) = |\mathbf{n}|$.

By adding the three terms from (i), (ii) and (iii), we obtain the stated expression. This concludes the proof. □

References

Brillinger, D. R. (2001) *Time series: data analysis and theory.* SIAM.

Cramér, H. (1946) *Mathematical Methods of Statistics.* Princeton, N.J.: Princeton Univ. Press.

Grenander, U. and Szegö, G. (1958) *Toeplitz Forms and Their Applications.* Berkeley, Calif.: Univ. Calif. Press.

Guillaumin, A. P., Sykulski, A. M., Olhede, S. C., Early, J. J. and Lilly, J. M. (2017) Analysis of non-stationary modulated time series with applications to oceanographic surface flow measurements. *J. Time Ser. Anal.*, **38**, 668–710.

Horn, R. A. and Johnson, C. R. (1985) *Matrix analysis.* Cambridge, UK: Cambridge Univ. Press.

Hosoya, Y. and Taniguchi, M. (1982) A central limit theorem for stationary processes and the parameter estimation of linear processes. *Ann. Stat.*, 132–153.

Körner, T. W. (1988) *Fourier Analysis.* Cambridge, UK: Cambridge Univ. Press.

Sykulski, A. M., Olhede, S. C., Guillaumin, A. P., Lilly, J. M. and Early, J. J. (2019) The debiased Whittle likelihood. *Biometrika*, **106**, 251–266.
